# Phospholipid scramblase 1 interacts with influenza A virus NP, impairing its nuclear import and thereby suppressing virus replication

**DOI:** 10.1371/journal.ppat.1006851

**Published:** 2018-01-19

**Authors:** Weiyu Luo, Jie Zhang, Libin Liang, Guangwen Wang, Qibing Li, Pengyang Zhu, Yuan Zhou, Junping Li, Yuhui Zhao, Nan Sun, Shanyu Huang, Chenchen Zhou, Yu Chang, Pengfei Cui, Pucheng Chen, Yongping Jiang, Guohua Deng, Zhigao Bu, Chengjun Li, Li Jiang, Hualan Chen

**Affiliations:** State Key Laboratory of Veterinary Biotechnology, Harbin Veterinary Research Institute, Chinese Academy of Agricultural Sciences, Harbin, China; University of Georgia, UNITED STATES

## Abstract

Transcription and replication of the influenza A virus (IAV) genome occur in the nucleus of infected cells and are carried out by the viral ribonucleoprotein complex (vRNP). As a major component of the vRNP complex, the viral nucleoprotein (NP) mediates the nuclear import of the vRNP complex via its nuclear localization signals (NLSs). Clearly, an effective way for the host to antagonize IAV infection would be by targeting vRNP nuclear import. Here, we identified phospholipid scramblase 1 (PLSCR1) as a binding partner of NP by using a yeast two-hybrid (Y2H) screen. The interaction between NP and PLSCR1 in mammalian cells was demonstrated by using co-immunoprecipitation and pull-down assays. We found that the stable overexpression of PLSCR1 suppressed the nuclear import of NP, hindered the virus life cycle, and significantly inhibited the replication of various influenza subtypes. In contrast, siRNA knockdown or CRISPR/Cas9 knockout of PLSCR1 increased virus propagation. Further analysis indicated that the inhibitory effect of PLSCR1 on the nuclear import of NP was not caused by affecting the phosphorylation status of NP or by stimulating the interferon (IFN) pathways. Instead, PLSCR1 was found to form a trimeric complex with NP and members of the importin α family, which inhibited the incorporation of importin β, a key mediator of the classical nuclear import pathway, into the complex, thus impairing the nuclear import of NP and suppressing virus replication. Our results demonstrate that PLSCR1 negatively regulates virus replication by interacting with NP in the cytoplasm and preventing its nuclear import.

## Author summary

Influenza viral RNA is encapsidated by three polymerase proteins and the NP protein to form the vRNP complex, which is transported to the nucleus of infected cells for viral transcription and replication. The active nuclear import of the vRNP complex is mediated by the interaction between NP and importin α through the nuclear import pathway. Because the interactions between NP and the components of the nuclear import pathway are indispensable in mediating the nuclear import of the vRNP complex, the host has evolved mechanisms to antagonize influenza virus infection that target this crucial step. In this study, we identified PLSCR1 as an interacting partner of the influenza NP protein. We found that PLSCR1 negatively regulates influenza virus replication by inhibiting the nuclear import of the NP/vRNP complex. Importantly, we found that PLSCR1 did not disrupt the interaction between NP and importin α. Instead, NP, PLSCR1, and importin α formed a stable complex that blocked the interaction between importin α and importin β, thereby inhibiting the import of NP/vRNP complex through the nuclear import pathway. Our findings provide an example of a host restriction factor binding simultaneously to a nuclear import adaptor and to a cargo protein to inhibit the import of that cargo into the nucleus.

## Introduction

Influenza A virus (IAV), a single-stranded, negative-sense RNA virus with an eight-segmented genome, is the causative agent of influenza in many animal species, including humans. Inside the virion, all eight viral RNA (vRNA) segments bind to the three RNA polymerases (polymerase basic protein 2, PB2; polymerase basic protein 1, PB1; and polymerase acidic protein, PA) and are encapsidated by the nucleoprotein (NP) to form viral ribonucleoprotein (vRNP) complexes [1]. The vRNP complex is the essential functional unit for the transcription and replication of the IAV genome [2]. Electron microscopy of isolated vRNPs has shown that both ends of the vRNA interact with each other to form a circular or supercoiled structure and that the RNA polymerase interacts with both ends of the vRNA segment [2–4]. The rest of the vRNA is encapsidated by the NP protein with approximately 24 nucleotides per molecule [5].

A prominent feature of the IAV life cycle is that the transcription and replication of the viral genome occur in the nucleus of infected cells [6, 7]. During the early phase of virus infection, after completion of endocytosis and uncoating, the vRNP complex is released into the cytoplasm and is translocated to the nucleus, which is mediated by the nuclear localization signals (NLSs) of the NP protein [8]. Two amino acid sequences have been identified as NLSs for the NP protein: an unconventional NLS in the N-terminus (residues 3 to 13; NLS1) [9, 10], and a bipartite NLS (residues 198 to 216; NLS2) [11]. The unconventional NLS appears to be the major determinant for NP nuclear import [12]. NP relies on the classical nuclear import pathway to enter the nucleus of infected cells. In this pathway, importin α functions as an adaptor by recognizing NLS sequences in cargo proteins and associating with the importin β receptor [13, 14]. Through a process that involves multiple rounds of interaction between importin β and nucleoporins of the nuclear pore complex (NPC), the trimeric importin α/β-cargo complex translocates into the nucleus [15]. NP interacts with various isoforms of importin α, including importin α-1, -3, -5, and -7 [10, 16, 17]. Previous studies have shown that the nuclear import of vRNP and newly synthesized NP to the nucleus of infected cells is a crucial step in the IAV life cycle [12, 18]. In addition to the central role played by importin α/β in modulating the nuclear transport of NP, host proteins could also be involved, such as α-actinin-4, Hsp40, or MOV10, which may promote or inhibit this active process [17, 19, 20]. However, the detailed mechanism that regulates the migration of vRNP complexes and newly produced NP into the nucleus remains obscure, and the identification of the potential host factors involved is not yet complete.

Phospholipid scramblase 1 (PLSCR1) was first identified in erythrocyte membranes, where it was activated under conditions of elevated calcium, resulting in disruption of phospholipid asymmetry across the plasma membrane [21]. Its function in remodeling the distribution of plasma membrane phospholipids in mammalian cells is still controversial because increase in PLSCR1 expression and gene depletion of PLSCR1 can occur in response to calcium without affecting the transmembrane movement of phospholipids [22–24]. In addition to its unresolved role as a scramblase, PLSCR1 appears to be involved in multiple biological processes. Several studies have shown that PLSCR1 plays a critical role in cellular maturation and terminal differentiation: PLSCR1 expression is markedly increased during the terminal differentiation of the monocytic and granulocytic lineages of hematopoietic precursor cells [25–27], and gene deletion of PLSCR1 in mice was found to impair the differentiation of hematopoietic precursor cells into mature granulocytes in response to select hematopoietic growth factors [24]. Although initially identified as a transmembrane protein, PLSCR1 also contains a nonclassical NLS and can be imported into the cell nucleus [28]. The nucleus-localized PLSCR1 can directly bind to the promoter region of the inositol 1,4,5-triphosphate receptor type 1 gene (IP3R1) to enhance its expression [29, 30], and can also interact with angiogenin (ANG) in the nucleus to positively regulate rRNA transcription [31]. Another important function of PLSCR1 is as an effector of the interferon (IFN) signaling pathway. PLSCR1 interacts with Toll-like receptor 9 (TLR9) and regulates its trafficking from the endoplasmic reticulum (ER) to the endosomal compartment in plasmacytoid dendritic cells (pDCs) [32], which is an important step in IFN production in pDCs. PLSCR1 harbors an IFN-stimulated response element in its first exon [23], is induced by IFN-α, -β, and -γ [23, 33, 34], and can enhance the expression of a subset of IFN-stimulated genes (ISGs) in response to IFN-β treatment to inhibit the replication of vesicular stomatitis virus (VSV) and encephalomyocarditis virus (EMCV) [33]. PLSCR1 mediates IFN-α-induced protection against staphylococcal α-toxin [35], is a main effector of IFN-γ-mediated antiviral activity against Hepatitis C virus (HCV) [34], and can also inhibit the replication of Hepatitis B virus (HBV) and human T-cell leukemia virus type-1 (HTLV-1) [36, 37].

In the present study, we discovered that the interaction between IAV NP and cellular PLSCR1 occurs in both transfected and infected mammalian cells. Importantly, overexpression of PLSCR1 significantly suppressed IAV replication, whereas siRNA knockdown or CRISPR/Cas9 knockout of PLSCR1 expression increased the virus titer, thereby demonstrating that PLSCR1 is a host restriction factor for IAV infection. We further found that PLSCR1 inhibited NP nuclear import and caused retardation of the virus life cycle. Strikingly, PLSCR1 formed an integrative complex with NP and different members of the importin α family, which inhibited the incorporation of importin β into the complex and impaired the import of NP via the nuclear import pathway.

## Results

### Identification of cellular proteins that interact with influenza virus NP protein

**Fig 1:**
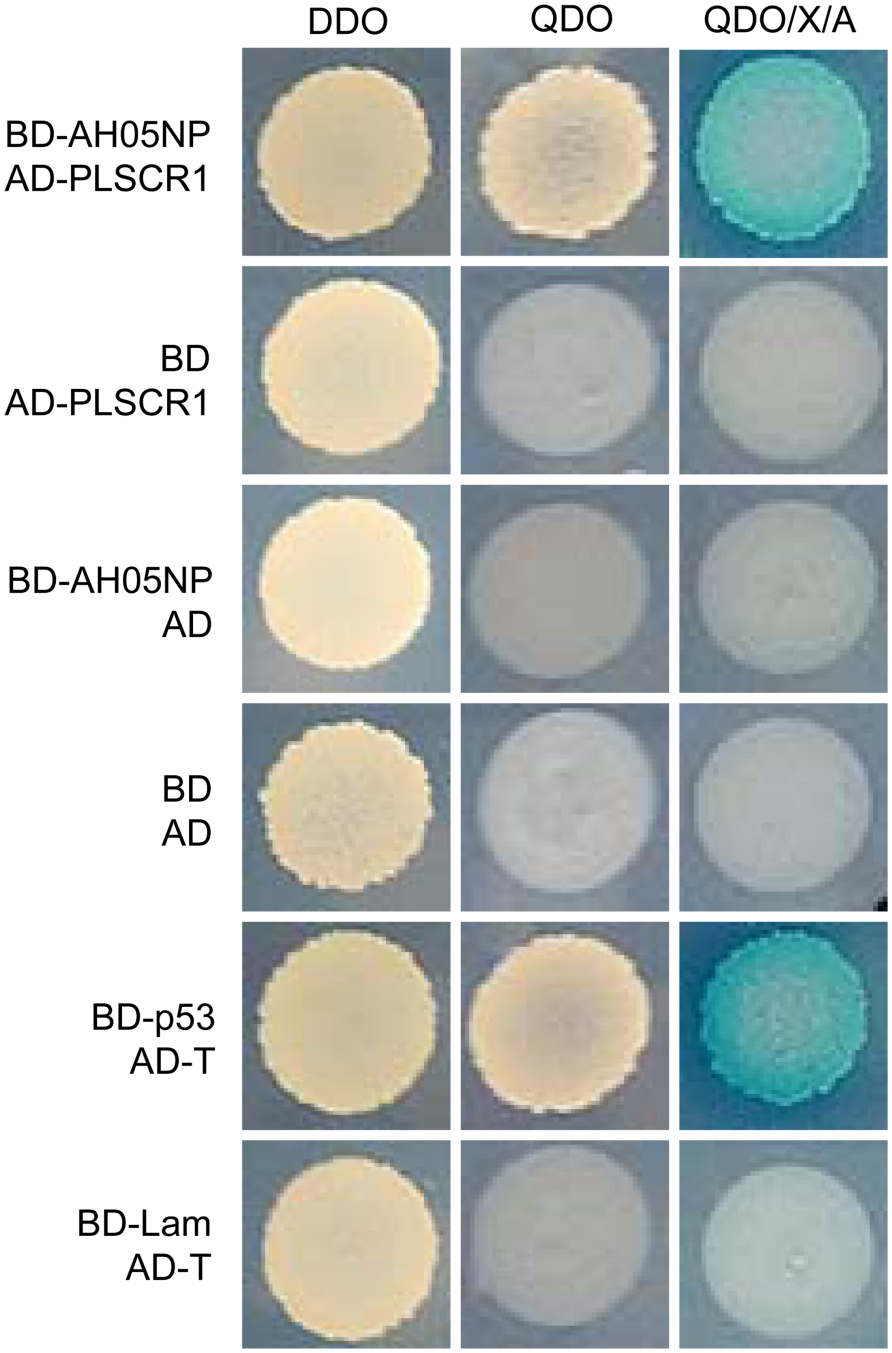
Yeast two-hybrid screen identifies PLSCR1 as an interacting partner of IAV NP. Yeast strain Y2HGold was cotransformed with the bait plasmid BD-AH05NP, containing AH05NP fused to the GAL4-binding domain (BD) in a pGBKT7 vector, together with the prey plasmid, AD-PLSCR1, which encodes PLSCR1 fused to the Gal4-activation domain (AD). Positive protein-protein interactions result in blue colonies in the QDO/X/A plates in the presence of X-a-Gal. Cotransformation of pGBKT7-53 encoding the Gal4-BD fused with murine p53 (BD-p53) and pGADT7-T encoding the Gal4-AD fused with SV40 large T-antigen (AD-T) served as a positive control. Cotransformation of pGBKT7-Lam, which encodes the Gal4-BD fused with lamin (BD-Lam) and AD-T served as a negative control. DDO, SD/−Leu/−Trp; QDO, SD/–Ade/–His/–Leu/–Trp; QDO/X/A, SD/–Ade/–His/–Leu/–Trp/X-a-Gal/AbA.

To identify host cellular proteins that interact with influenza virus NP protein, we employed the yeast two-hybrid system to screen a cDNA library generated from a mixed human cell culture (A549, HEK293T, THP-1, and U251) as described previously [38]. The full-length NP protein from A/Anhui/2/2005 (AH05, H5N1) was used as bait. Putative positive clones were obtained after selection on QDO/X/A (Ade/–His/–Leu/–Trp/X-a-Gal/AbA) plates. After growing the putative positive clones in DDO (SD/−Leu/−Trp) medium, plasmids were isolated and sequenced to identify the potential NP interactants. One specific clone from this screen was found to contain the full-length open reading frame of PLSCR1 (GenBank accession no. NM_021105). The interaction between PLSCR1 and NP was then retested by yeast co-transformation, as described in the Materials and Methods. As shown in Fig 1, PLSCR1 specifically interacted with NP in yeast.

### PLSCR1 interacts with NP

**Fig 2:**
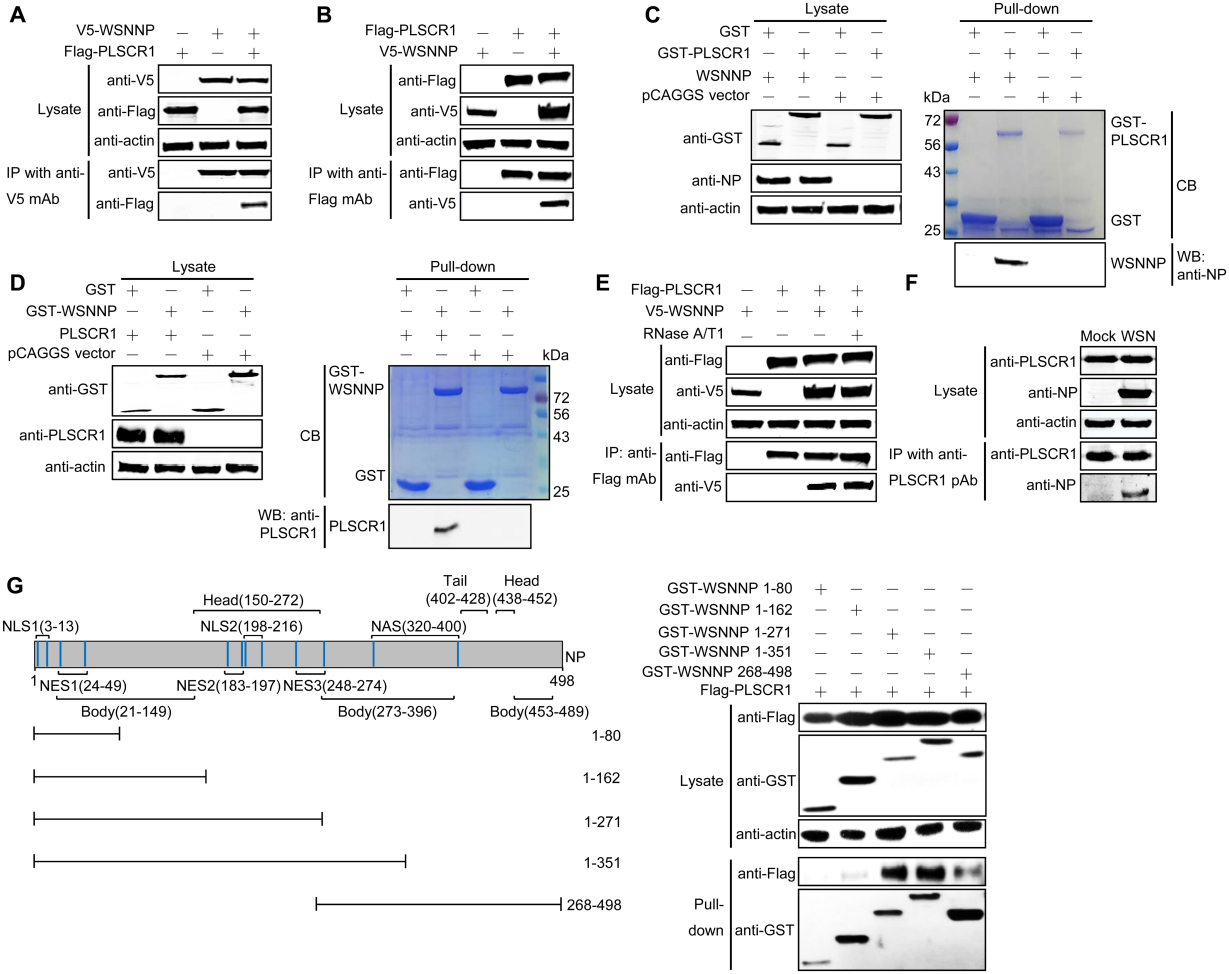
NP interacts with PLSCR1 in mammalian cells. (A, B) Co-IP assay of V5-NP and Flag-PLSCR1 in HEK293T cells. HEK293T cells were transfected individually or in combination with plasmids expressing V5-WSNNP and Flag-PLSCR1. Cell lysates were immunoprecipitated with a mouse anti-V5 mAb (A) or a mouse anti-Flag mAb (B) and were subjected to western blotting with a rabbit anti-V5 pAb or a rabbit anti-Flag pAb to reveal the presence of NP and PLSCR1, respectively. (C, D) GST pull-down assay of NP and PLSCR1. Lysates of HEK293T cells transfected with the GST or GST-PLSCR1 construct were incubated with Glutathione Sepharose 4 Fast Flow and then mixed with lysates from cells transfected with pCAGGS or pCAGGS-WSNNP (C); HEK293T cell lysates containing exogenously expressed GST or GST-WSNNP were incubated with Glutathione Sepharose 4 Fast Flow and then mixed with lysates from cells transfected with pCAGGS or pCAGGS-PLSCR1 (D). After washing away the unbound proteins, equal volumes of proteins bound to the beads and the original cell lysates (5% of the input) were examined by western blotting using a rabbit anti-NP pAb, a rabbit anti-GST pAb, or a rabbit anti-PLSCR1 pAb. GST, GST-PLSCR1, or GST-WSNNP proteins in the eluates were detected by Coomassie blue (CB) staining. (E) The NP-PLSCR1 interaction does not rely on RNA binding. HEK293T cells were transfected individually or in combination with plasmids expressing V5-WSNNP and Flag-PLSCR1. Cell lysates treated with RNase A/T1 or left untreated were immunoprecipitated with a mouse anti-Flag mAb and were subjected to western blotting with a rabbit anti-V5 pAb or a rabbit anti-Flag pAb to reveal the presence of NP and PLSCR1, respectively. (F) PLSCR1 interacts with NP during natural viral infection. Confluent A549 cells grown in 6-well plates were mock infected with PBS or infected with WSN virus at an MOI of 5. At 6 h p.i., cell lysates were immunoprecipitated with a rabbit anti-PLSCR1 pAb and were subjected to western blotting with a mouse anti-NP mAb or a rabbit anti-PLSCR1 pAb to detect NP and PLSCR1, respectively. (G) Mapping of the PLSCR1-interacting domain within NP. Schematic presentation of influenza NP showing the different domains as well as the truncation mutants made in this study is on the left side. The interaction between PLSCR1 and the NP truncation mutants is shown on the right side. Lysates of HEK293T cells were pulled down with glutathione magnetic beads. The bound proteins were subjected to western blotting with a rabbit anti-Flag pAb or a rabbit anti-GST pAb to reveal the presence of PLSCR1 and NP, respectively. NES, nuclear export signal; NAS, nuclear accumulation signal.

To further examine the PLSCR1-NP interaction, we performed co-IP experiments. HEK293T cells were transfected with V5-tagged WSN NP and Flag-tagged PLSCR1, individually or in combination. Cell lysates were immunoprecipitated with an anti-V5 mAb, followed by western blotting with rabbit pAb against V5 or the Flag tag (Fig 2A). Flag-tagged PLSCR1 was coimmunoprecipitated with V5-tagged NP of A/WSN/33 (WSN, H1N1) virus when they were coexpressed, but not in the absence of WSN NP, indicating that PLSCR1 interacts with influenza NP in mammalian cells. When a reverse co-IP experiment was performed with an anti-Flag mAb, V5-tagged WSN NP was also coimmunoprecipitated with Flag-tagged PLSCR1 (Fig 2B), further demonstrating the specificity of the NP-PLSCR1 interaction. The PLSCR1-NP interaction was also confirmed in a GST pull-down assay. WSN NP was pulled down by GST-PLSCR1, but not by GST alone (right panel, Fig 2C). Similarly, PLSCR1 was only pulled down by GST-WSNNP (right panel, Fig 2D).

A key function of influenza NP protein during the virus life cycle is to encapsidate viral RNA to form the vRNP complex in preparation for transcription, replication, and packaging [39]. To examine whether the interaction between PLSCR1 and NP is dependent on the RNA-binding activity of NP, we performed a co-IP assay with cell lysates that were first treated with 100 μl of RNase A/T1 (Fig 2E). Flag-PLSCR1 was still coimmunoprecipitated with V5-WSNNP, indicating that the interaction between PLSCR1 and NP did not rely on the RNA binding activity of NP.

We performed an additional co-IP experiment in A549 cells that were mock infected or infected with WSN virus at an MOI of 5. At 6 h post infection (p.i.), cell lysates were immunoprecipitated with a rabbit pAb against PLSCR1, followed by western blotting with a rabbit anti-PLSCR1 pAb for the detection of PLSCR1 and a mouse anti-NP mAb to reveal the presence of NP (Fig 2F). The results showed that WSN NP interacted with PLSCR1 during the natural viral infection.

We then attempted to define the region of NP that was critical for its binding with PLSCR1. We generated five truncated NP constructs (NP1-80, NP1-162, NP1-271, NP1-351, and NP268-498), which were fused to the C-terminus of GST, and then examined their interaction with PLSCR1 in HEK293T cells. We found that all five truncated versions of NP were well expressed, although there were differences in their expression levels (Fig 2G). The pull-down assay showed that NP1-271 and NP1-351 exhibited strong binding to PLSCR1. In contrast, the two short N-terminal NP mutants, NP1-80 and NP1-162, almost lost their ability to interact with PLSCR1. Further, the interaction between the C-terminal NP mutant, NP268-498, and PLSCR1 was also dramatically decreased compared with that of NP1-271 and NP1-351. These results indicate that neither the N-terminal nor the C-terminal region of NP is critical for its interaction with PLSCR1; rather, the middle region of NP is likely involved in the interaction with PLSCR1.

### PLSCR1 suppresses influenza virus replication

**Fig 3:**
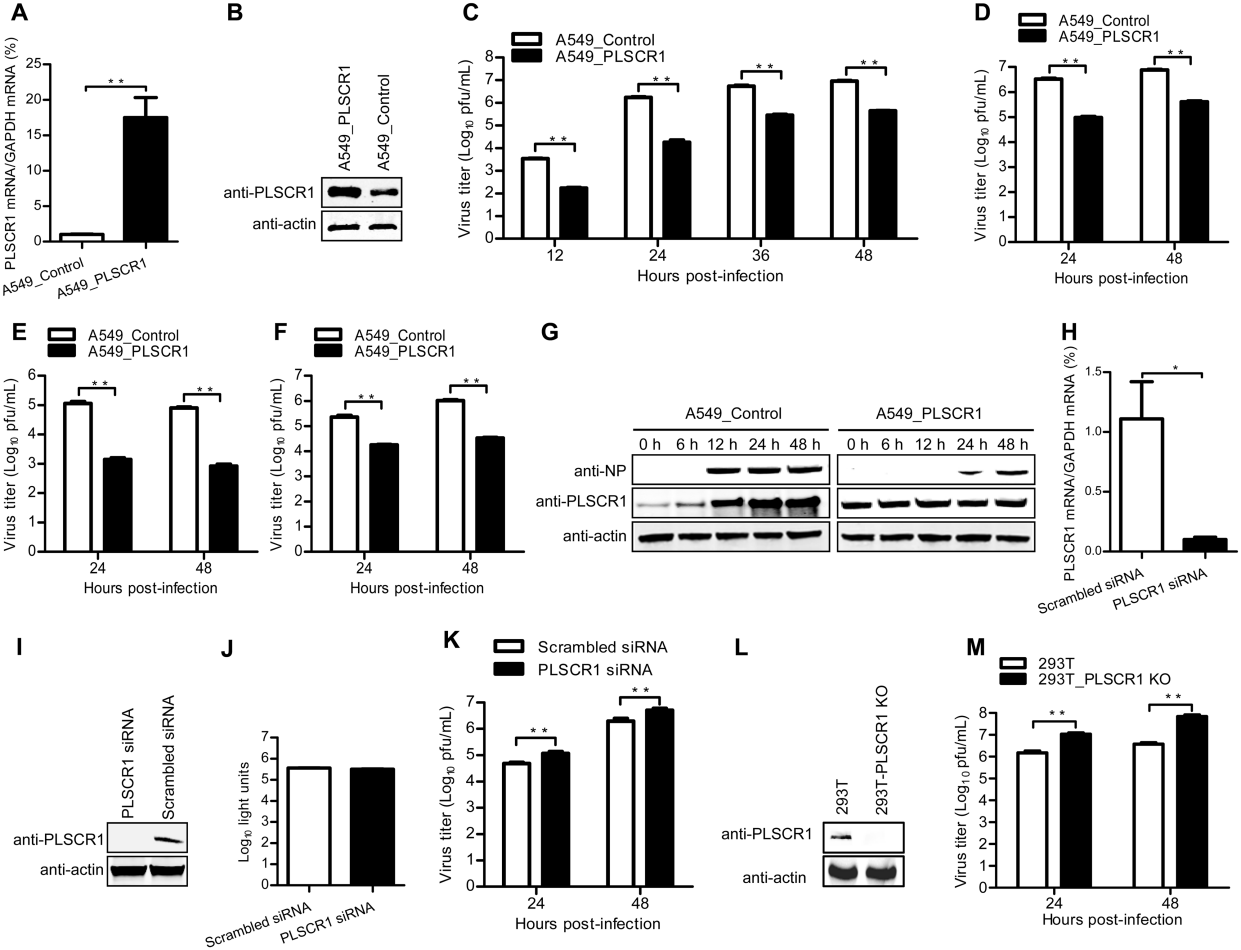
PLSCR1 negatively regulates influenza virus replication. (A, B) Establishment of an A549 cell line stably overexpressing PLSCR1. The stable overexpression of PLSCR1 was confirmed by quantitative reverse-transcription PCR (RT-qPCR) (A) and western blotting with a rabbit anti-PLSCR1 pAb (B) in comparison with the A549 control cell line transduced with an empty retrovirus. **, *P* < 0.01. (C, D, E, F) Virus replication in PLSCR1-overexpressing A549 cells. The PLSCR1-overexpressing or empty retrovirus-transduced control A549 cells were infected with WSN (H1N1) (C), AH05 (H5N1) (D), AH13 (H7N9) (E) or FZ09 (H1N1) (F) at an MOI of 0.1. Supernatants were collected at the indicated timepoints, and virus titers were determined by means of plaque assays on MDCK cells. **, *P* < 0.01. (G) Expression of PLSCR1 and NP in virus-infected cells. The PLSCR1-overexpressing or empty retrovirus-transduced control A549 cells were infected with WSN virus at an MOI of 0.1. Whole cell lysates were collected at the indicated timepoints and subjected to western blotting with a rabbit anti-PLSCR1 pAb or a rabbit anti-NP pAb. (H, I) siRNA knockdown of PLSCR1 in A549 cells. A549 cells were transfected with siRNA targeting PLSCR1 or with scrambled siRNA for 48 h. Whole cell lysates were then collected and analyzed by RT-qPCR (H) or western blotting with a rabbit anti-PLSCR1 pAb (I). *, *P* < 0.05. (J) Cell viability of siRNA-treated A549 cells was measured by using a CellTiter-Glo assay. The data are presented as means ± standard deviations (SD) for triplicate transfections. (K) Virus replication in siRNA-treated A549 cells. Cells transfected with siRNA were infected with WSN virus at an MOI of 0.1. Supernatants were collected at 24 and 48 h p.i. and titrated for infectious virus by means of plaque assays on MDCK cells. **, *P* < 0.01. (L) Generation of PLSCR1-KO HEK293T cells. PLSCR1-KO cells were generated by using the CRISPR/Cas9 system. PLSCR1 knockout was confirmed by western blotting with a rabbit anti-PLSCR1 pAb. (M) Virus replication in PLSCR1-KO HEK293T cells. PLSCR1-KO HEK293T or control cells were infected with WSN virus at an MOI of 0.1. Supernatants were collected at 24 and 48 h p.i., and virus titers were determined by means of plaque assays on MDCK cells. **, *P* < 0.01.

To study the role of the PLSCR1-NP interaction during the virus life cycle, we analyzed the effect of upregulating PLSCR1 on virus replication. We transduced A549 cells with a retrovirus encoding PLSCR1 to establish a stable PLSCR1-overexpressing cell line or with an empty retrovirus as a control cell line. As expected, PLSCR1 expression at both the mRNA and protein level was increased in PLSCR1-overexpressing cells compared with the empty retrovirus-transduced control cells (Fig 3A and 3B). The control and PLSCR1-overexpressing A549 cells were infected with WSN virus at an MOI of 0.1. Culture supernatants were collected at different timepoints after infection and titrated on MDCK cells. Strikingly, PLSCR1 overexpression led to a 20- to 100-fold decrease in virus titers at 12–48 h p.i. (Fig 3C). Similar reductions in virus titers were observed for influenza viruses AH05 (H5N1) (Fig 3D), A/Anhui/1/2013 (H7N9) (Fig 3E) and A/Fuzhou/1/2009 (H1N1) (Fig 3F) at both 24 h and 48 h p.i. In a separate experiment, we examined NP and PLSCR1 expression in WSN virus-infected cells at timepoints between 0 and 48 h p.i. (Fig 3G). In control A549 cells, viral NP expression was abundant at 12 h p.i., and remained high until 48 h. In clear contrast, less NP was detected at 24 h p.i., with somewhat more detected at 48 h p.i. in PLSCR1-overexpressing A549 cells. Moreover, the expression of PLSCR1 remained unchanged in virus-infected PLSCR1-overexpressing A549 cells, whereas in control A549 cells, the expression of PLSCR1 was upregulated at 6 h p.i., increased at 12 h p.i., and remained elevated at 48 h p.i. Together, these data indicate that endogenous expression of PLSCR1 is strongly induced by influenza virus infection, and stable overexpression of PLSCR1 significantly inhibits NP expression and virus replication.

We further analyzed the effect of PLSCR1 downregulation on IAV infection by means of small interfering RNA (siRNA)-mediated silencing. Real-time PCR and western blotting confirmed that the expression of PLSCR1 was significantly reduced in specific siRNA-treated A549 cells but not in cells treated with scrambled siRNA (Fig 3H and 3I). PLSCR1 downregulation had no major effect on cell viability as measured by a luminescent cell viability assay (Fig 3J). A549 cells treated with siRNA targeting PLSCR1 or with scrambled siRNA were infected with WSN virus. Culture supernatants were collected at 24 and 48 h p.i. and titrated on MDCK cells. As shown in Fig 3K, knockdown of PLSCR1 by specific siRNA increased the virus titer compared with that in scrambled siRNA-treated A549 cells. We further generated a PLSCR1-KO HEK293T cell line by using the CRISPR/Cas9 system. The knockout of PLSCR1 was confirmed by western blotting with a rabbit anti-PLSCR1 pAb (Fig 3L). The PLSCR1-KO HEK293T or control cells were infected with WSN virus at an MOI of 0.1, and the supernatants collected at 24 and 48 h p.i. were titrated on MDCK cells. As shown in Fig 3M, the titers of WSN virus in PLSCR1-KO HEK293T cells were dramatically increased compared with those of the control cells. Together, these data demonstrate that PLSCR1 negatively regulates IAV replication via its interaction with NP.

### PLSCR1 inhibits the nuclear import of NP and causes retardation of the virus replication cycle

**Fig 4:**
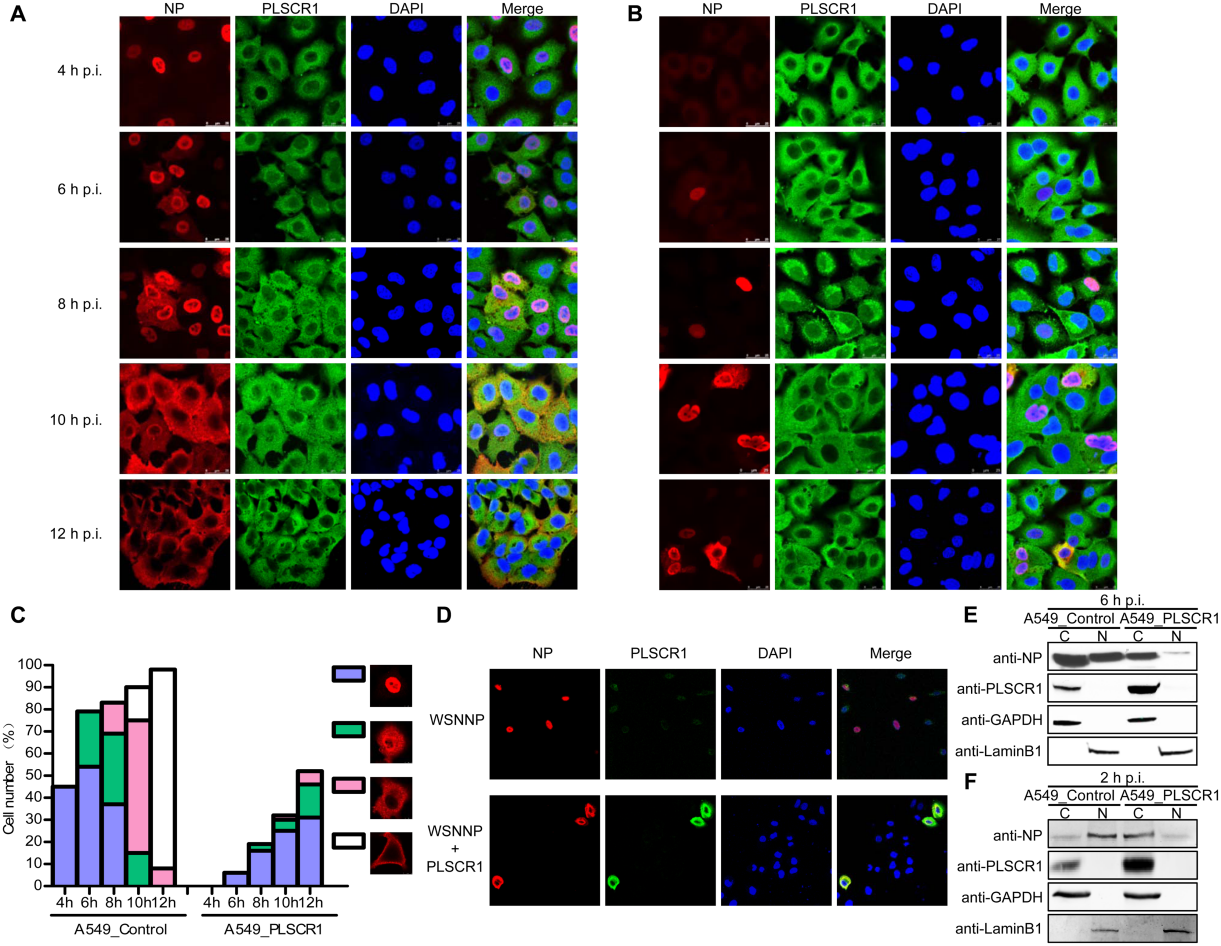
PLSCR1 inhibits the nuclear import of NP. (A, B) Empty retrovirus-transduced control A549 cells (A) or PLSCR1-overexpressing A549 cells (B) were infected with WSN virus at an MOI of 5. At 4, 6, 8, 10, and 12 h p.i., the infected cells were fixed and stained with mouse anti-NP mAb and rabbit anti-PLSCR1 pAb, followed by incubation with Alexa Fluor 488 donkey anti-rabbit IgG (H+L) (green) and Alexa Fluor 633 goat anti-mouse IgG (H+L) (red). The nuclei were stained with DAPI. (C) Quantitative analysis of NP localization in virus-infected cells. On the basis of the confocal microscopy in panels A and B, the localization of NP (indicative of vRNP) after nuclear import was categorized into four types, clear nuclear localization, simultaneous localization at the edge of the nucleus and the cytoplasm, predominant cytoplasmic localization, and close to the cytoplasmic membrane. The results shown are calculated from one hundred cells viewed under a confocal microscope with a 40X objective lens. (D). PLSCR1 inhibits the nuclear import of NP in transfected A549 cells. A549 cells were transfected with pCAGGS-WSNNP alone or were cotransfected with pCAGGS-WSNNP and pCAGGS-PLSCR1 and were assessed by confocal microscopy. NP was detected with a mouse anti-NP mAb and visualized with Alexa Fluor 633 (red). PLSCR1 was detected with a rabbit anti-PLSCR1 pAb and visualized with Alexa Fluor 488 (green). Yellow in the merged image indicates the colocalization of NP and PLSCR1. (E) PLSCR1-overexpressing or empty retrovirus-transduced control A549 cells were infected with WSN virus at an MOI of 5. At 6 h p.i., the cells were separated into nuclear (N) and cytoplasmic fractions (C). Each fraction was subjected to western blotting with a rabbit anti-NP pAb and a rabbit anti-PLSCR1 pAb for protein detection. (F) PLSCR1-overexpressing A549 cells or control A549 cells were treated with CHX to inhibit protein synthesis. The treated cells were infected with WSN virus at an MOI of 5, and were separated into nuclear and cytoplasmic fractions at 2 h p.i., followed by western blotting to detect the amount of NP in the nuclear and cytoplasmic fractions.

The cellular distribution of NP during the virus life cycle was investigated by using a time-course experiment in both PLSCR1-overexpressing and empty retrovirus-transduced control A549 cells infected with WSN virus at an MOI of 5. In the control A549 cells, NP had clearly accumulated in the nucleus of approximately 45% of cells at 4 h p.i (Fig 4A and 4C). By 6 h p.i., the percentage of cells with NP in the nucleus had increased to 54%. In addition, NP localized at both the edge of nucleus and the cytoplasm of 25% of the infected cells, an indication of vRNP export from the nucleus. At 8 h p.i., the distribution of NP was mixed, with approximately 37%, 32%, and 14% of cells showing clear nuclear localization, simultaneous localization at both the edge of the nucleus and the cytoplasm, and exclusive cytoplasmic distribution, respectively. At 10 h p.i., the newly synthesized vRNP complex was largely exported from the nucleus into the cytoplasm, as indicated by the cytoplasmic distribution of NP in 60% of the cells. NP was also primarily localized close to the cytoplasmic membrane in 15% of the cells. By 12 h p.i., the percentage of cells with NP distributed close to the cytoplasmic membrane was 90%, indicating that vRNP export was largely complete and active assembly and budding were underway. The endogenous PLSCR1 was predominantly localized in the cytoplasm of the control A549 cells throughout the observation period from 4 to 12 h p.i (Fig 4A). The colocalization of NP and PLSCR1 appeared in cells with obvious cytoplasmic distribution of NP at 8 h p.i. At 10 and 12 h p.i., a large amount of newly synthesized vRNP complex was visualized in the cytoplasm or close to the cytoplasmic membrane, where obvious colocalization of NP and PLSCR1 was observed. In comparison with the control A549 cells, the virus life cycle was significantly delayed in the PLSCR1-overexpressing A549 cells (Fig 4B and 4C). At 4 h p.i., NP did not accumulate in the nucleus of any of the visualized cells, suggesting that the nuclear import of the vRNP was inhibited by the overexpressed PLSCR1. At 6 h p.i., only approximately 6% of cells showed clear nuclear accumulation of NP. At 8 h p.i., NP had clearly accumulated in the nucleus of approximately 16% of cells, and roughly 3% of cells had NP at both the edge of the nucleus and the cytoplasm. At 10 h p.i., NP showed clear nuclear localization, simultaneous localization at both the edge of the nucleus and the cytoplasm, and exclusive cytoplasmic distribution in 25%, 5%, and 2% of cells, respectively. At 12 h p.i., the NP distribution pattern was similar to that at 10 h p.i. except that the percentages of cells showing clear nuclear localization, simultaneous localization at both the edge of the nucleus and the cytoplasm, and exclusive cytoplasmic distribution were further increased to 31%, 15%, and 6% respectively. As in the control A549 cells, PLSCR1 was almost exclusively localized in the cytoplasm of the PLSCR1-overexpressing cells (Fig 4B). In addition, co-localization of NP and PLSCR1 was detected in the cytoplasm of cells exhibiting considerable vRNP export at 10 and 12 h p.i. Taken together, these data demonstrate that overexpression of PLSCR1 significantly inhibits the nuclear import of the vRNP complex, and causes dramatic retardation of the virus life cycle.

At the early timepoints (i.e., 4 and 6 h p.i. in control cells, and 4, 6, and 8 h p.i. in PLSCR1-overexpressing cells), co-localization of NP and PLSCR1 was not observed in the cytoplasm, most likely because of the relatively low abundance of the vRNPs in the cytoplasm prior to their import into the nucleus. We attempted to determine whether the interaction between NP and PLSCR1 could directly inhibit the import of NP into the nucleus. To this end, we transfected A549 cells with a pCAGGS-WSNNP construct together with either pCAGGS-PLSCR1 or the empty vector. The localization of NP and PLSCR1 was visualized at 20 h post-transfection. As shown in Fig 4D, NP clearly accumulated in the nucleus of cells without exogenous PLSCR1 expression. In contrast, NP was predominantly retained in the cytoplasm and colocalized with PLSCR1 when PLSCR1 was substantially overexpressed. These results further confirm that PLSCR1 inhibits the import of NP into the nucleus.

We next validated the inhibitory effect of PLSCR1 on the nuclear import of NP with a cell fractionation experiment. The PLSCR1-overexpressing or empty retrovirus-transduced control A549 cells were infected with WSN virus at an MOI of 5. At 6 h p.i., the infected cells were lysed. The cytoplasmic and nuclear fractions were separated and subjected to western blotting. As shown in Fig 4E, the marker proteins GAPDH and LaminB1 were only detected in the cytoplasm and nucleus, respectively. PLSCR1 almost exclusively localized in the cytoplasm in both PLSCR1-overexpressing and control cells. A considerable amount of NP was detected in both the nucleus and the cytoplasm of the control cells. In contrast, NP was primarily detected in the cytoplasm and was only weakly detected in the nucleus of the PLSCR1-overexpressing cells.

We further investigated the inhibitory role of PLSCR1 on the nuclear import of incoming vRNPs by treating PLSCR1-overexpressing A549 cells or control A549 cells with cycloheximide (CHX) to inhibit protein synthesis. The treated cells were infected with WSN virus at an MOI of 5, and were separated into nuclear and cytoplasmic fractions at 2 h p.i., followed by western blotting to detect the NP in the nuclear and cytoplasmic fractions. We found that most of the NP was detected in the nucleus of the control A549 cells; however, in PLSCR1-overexpressing A549 cells, most of the NP was detected in the cytoplasm and NP was only weakly detected in the nucleus (Fig 4F). Since the only source of NP protein was from the incoming vRNPs under CHX treatment, this experiment demonstrates that PLSCR1 directly inhibits the nuclear import of incoming vRNPs.

Collectively, these results demonstrate that the expression of PLSCR1 suppresses the nuclear accumulation of NP/vRNP, thus inhibiting the virus life cycle.

### PLSCR1 inhibits viral transcription and replication

**Fig 5:**
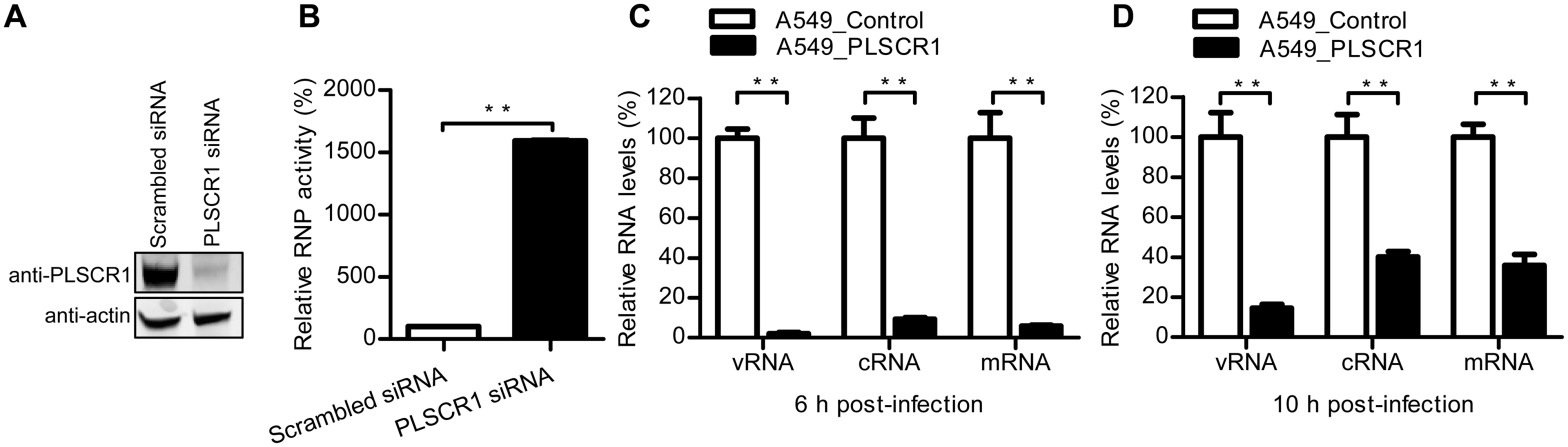
PLSCR1 inhibits influenza virus transcription and replication. (A) HEK293T cells were transfected with siRNA targeting PLSCR1 or with scrambled siRNA for 48 h. The knockdown of PLSCR1 expression was confirmed by western blotting. (B) HEK293T cells treated with siRNA were transfected with the four RNP protein expression constructs (PB2, PB1, PA and, NP) derived from WSN virus, together with pHH21-SC09NS F-Luc, which encodes NS vRNA possessing a reporter firefly luciferase gene. At 48 h post-transfection, a dual-luciferase assay was performed in which the relative firefly luciferase activity was normalized to the internal control, Renilla luciferase activity. **, *P* < 0.01. (C, D) The PLSCR1-overexpressing or empty retrovirus-transduced control A549 cells were infected with WSN virus at an MOI of 5. Total RNA was harvested at 6 h (C) and 10 h (D) p.i. NP-specific vRNA, cRNA, and mRNA were analyzed by RT-qPCR and then normalized to GAPDH mRNA. The values shown are standardized to the corresponding RNA expression level in the control A549 cells (100%). **, *P* < 0.01.

We hypothesized that viral RNA transcription and replication would be impaired due to the retention of vRNP and NP in the cytoplasm caused by PLSCR1 expression. To test this hypothesis, we transfected HEK293T cells with specific siRNA targeting PLSCR1 or with scrambled siRNA for 48 h. Western blotting analysis showed that specific siRNA treatment indeed downregulated the expression of PLSCR1 (Fig 5A). The siRNA-treated cells were then transfected with protein expression constructs of the RNP complex proteins (PB2, PB1, PA, and NP), along with a reporter plasmid containing the terminal coding and noncoding sequences from the NS segment and the luciferase gene driven by the human RNA polymerase I promoter and terminator. Forty-eight hours later, the luciferase activity of the cell lysates was measured to reveal the RNP activity. We found that the RNP activity was increased by approximately 16-fold when the expression of PLSCR1 was knocked down by specific siRNA compared with that in scrambled siRNA-treated cells (Fig 5B), indicating that the endogenous PLSCR1 inhibited the transcription and replication of the viral genome.

To further determine the steps of viral transcription and replication that were affected by PLSCR1 expression, we infected the PLSCR1-overexpressing and empty retrovirus-transduced control A549 cells with WSN virus at an MOI of 5. At 6 and 10 h p.i., vRNA, mRNA, and cRNA derived from segment 5 were measured by quantitative reverse transcription PCR (RT-qPCR). At both timepoints, the levels of all three species of viral RNA were found to be significantly decreased in the PLSCR1-overexpressing cells compared with those in the control cells (Fig 5C and 5D). Among the three species of viral RNA, the reduction in the vRNA level was the most sizeable. This finding could indicate that vRNA synthesis occurred after the synthesis of the mRNA and cRNA, and reductions in the synthesis of mRNA and cRNA would lead to an accumulative defect in the amplification of the vRNA species.

### PLSCR1 does not affect the phosphorylation status of the NP protein

**Fig 6:**
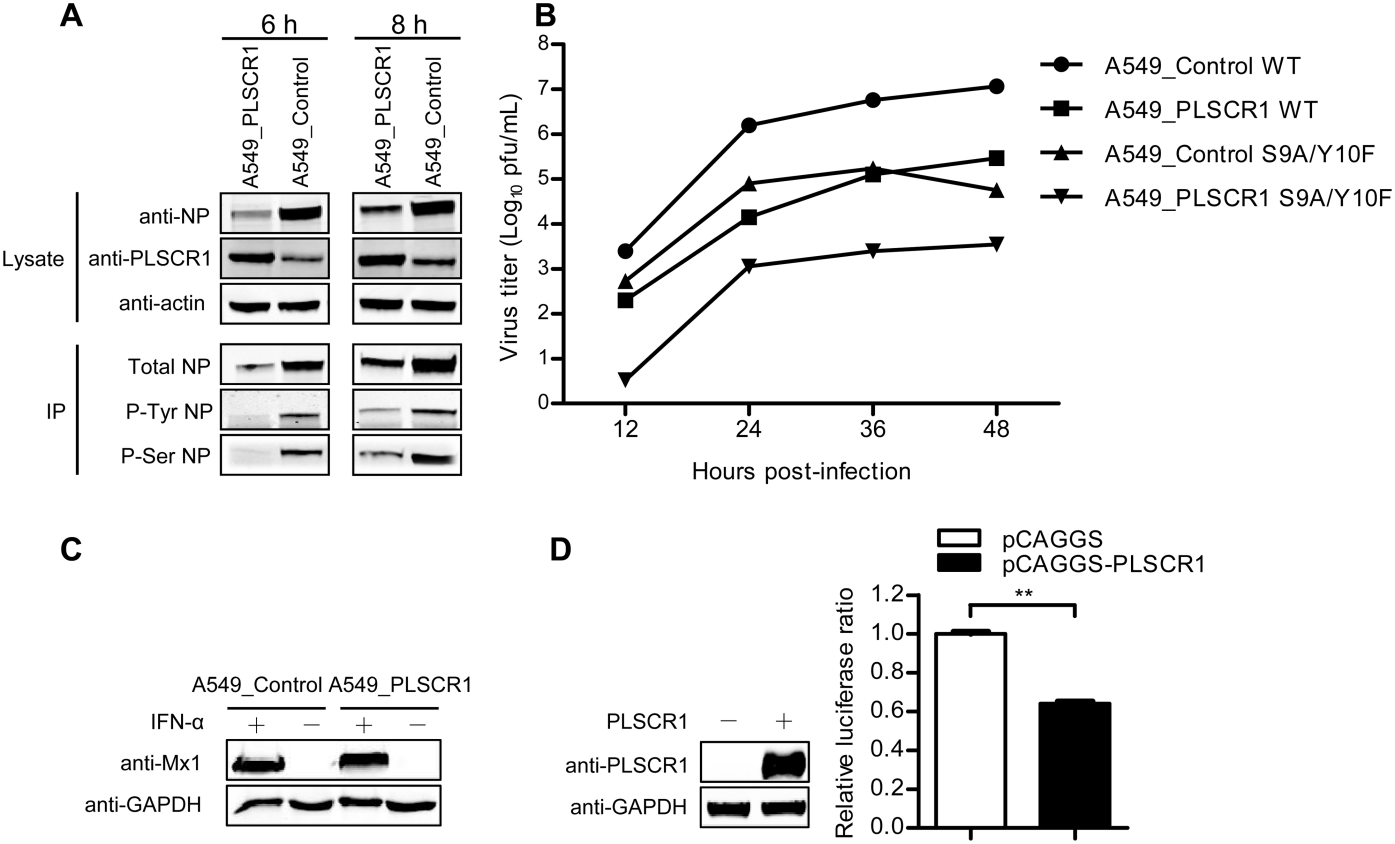
PLSCR1 does not affect the phosphorylation status of NP and does not stimulate the IFN pathways. (A) PLSCR1-overexpressing or empty retrovirus-transduced control A549 cells were infected with WSN virus at an MOI of 5. Cell lysates were processed at 6 h and 8 h p.i., immunoprecipitated with a mouse anti-NP mAb, a mouse anti-p-Ser mAb, or a mouse anti-p-Tyr mAb, followed by western blotting with a rabbit anti-NP pAb to detect the level of total NP, serine-phosphorylated NP, and tyrosine-phosphorylated NP, respectively. (B) Replication of an NP-phosphorylation mutant virus in PLSCR1-overexpressing A549 cells. PLSCR1-overexpressing or empty retrovirus-transduced control A549 cells were infected with wild-type WSN virus or the phosphorylation mutant S9A/Y10F at an MOI of 0.1. Supernatants were collected at the indicated timepoints and titrated for infectious virus by means of plaque assay on MDCK cells. (C) Expression of Mx1 protein in PLSCR1-overexpressing or control A549 cells. PLSCR1-overexpressing or empty retrovirus-transduced control A549 cells were grown in 12-well plates, and were left untreated or were treated with IFN-α for 24 h. The cell lysates were then subjected to western blotting with a rabbit anti-Mx1 pAb for the detection of Mx1 protein. GAPDH, detected by a rabbit anti-GAPDH pAb, served as a negative control. (D) Expression of the ISRE luciferase reporter gene in HEK293T cells transfected with the PLSCR1-expressing construct or empty vector. HEK293T cells were transfected with the ISRE-Luc reporter plasmid, pRL-TK control plasmid, and the pCAGGS-PLSCR1 or empty pCAGGS plasmid for 20 h. The overexpression of PLSCR1 was confirmed by western blotting with a rabbit anti-PLSCR1 pAb. The luciferase activity of the transfected cells was analyzed by using the Dual-Luciferase reporter assay. After normalization with co-transfected Renilla luciferase activity, the relative firefly luciferase activity of PLSCR1-overexpressing cells was expressed as the fold-induction of the ISRE firefly luciferase activity compared to cells transfected with empty pCAGGS vector. **, *P* < 0.01.

The nuclear import of influenza NP protein can be regulated via its phosphorylation status [9, 40]. We therefore determined whether the effect of PLSCR1 on the import of NP is achieved by modulating NP phosphorylation. We infected either PLSCR1-overexpressing or empty retrovirus-transduced control A549 cells with WSN virus at an MOI of 5. At 6 and 8 h p.i., the NP and PLSCR1 expression levels in the infected cells were determined by western blotting (Fig 6A). In PLSCR1-overexpressing cells, the level of PLSCR1 remained relatively constant between the two timepoints, whereas the expression of viral NP protein was increased at 8 h compared with that at 6 h p.i. In contrast, in control A549 cells, the increase in PLSCR1 expression was more obvious than that of NP between the two timepoints. We then performed an immunoprecipitation experiment by using an anti-NP mAb to reveal the level of total NP, an anti-p-Ser mAb to determine the level of serine-phosphorylated NP, and an anti-p-Tyr mAb to detect the level of tyrosine-phosphorylated NP. At 6 h p.i., NP was clearly serine- and tyrosine-phosphorylated in control cells, and the extent of NP phosphorylation was further increased at 8 h p.i. In PLSCR1-overexpressing cells, NP phosphorylation was barely detectable at 6 h p.i., the timepoint when the expression of total NP was only weakly detected. In contrast, a considerable amount of NP was phosphorylated at 8 h p.i. In general, NP was less phosphorylated at both timepoints in the PLSCR1-overexpressing cells compared with the control cells. However, the proportion of phosphorylated NP of the total NP, as indicated at 8 h p.i., was similar between the PLSCR1-overexpressing cells and the control cells. These data indicate that overexpression of PLSCR1 does not affect the phosphorylation status of the viral NP protein.

Two phosphorylation sites in NP, S9 and Y10, are highly conserved among all influenza A viruses [41]. Mutations that abolish these two sites have been shown to significantly reduce virus replication [40, 41]. Here, we examined the growth properties of a phosphorylation mutant, S9A/Y10F, in PLSCR1-overexpressing A549 cells. As shown in Fig 6B, we observed an additive effect of the inhibitory role of PLSCR1 overexpression and that of mutation of key NP phosphorylation sites on virus replication. The replication of wild-type WSN virus was significantly inhibited in PLSCR1-overexpressing A549 cells compared with control cells (Fig 6B). Moreover, the replication of the phosphorylation mutant S9A/Y10F was decreased further in PLSCR1-overexpressing cells than in control cells. These results indicate that regardless of the phosphorylation status of the NP residues S9 and Y10, PLSCR1 overexpression consistently reduced virus replication, implying that the inhibitory role of PLSCR1 on virus replication is not played by influencing the phosphorylation status of NP.

### The inhibitory effect of PLSCR1 on virus replication does not rely on the stimulation of IFN pathways

PLSCR1 can potentiate the antiviral activity of IFN when exogenous IFN is present [33]. We therefore investigated the possibility that PLSCR1 indirectly inhibits virus infection by stimulating the IFN pathway. To this end, we measured the protein level of Mx1, a key antiviral effector protein of the IFN pathway, in both PLSCR1-overexpressing A549 cells and control A549 cells. As shown in Fig 6C, Mx1 expression was not detectable in either PLSCR1-overexpressing cells or control cells when they were not treated with IFN-α. In contrast, IFN-α treatment efficiently induced the expression of Mx1 protein in both types of cells. However, no difference in the level of Mx1 expression was observed between PLSCR1-overexpressing cells and control cells when they were treated with IFN-α. Therefore, the overexpression of PLSCR1 did not result in observable changes in the expression of Mx1 relative to the control cells. We also determined the luciferase activity of HEK293T cells transfected with an ISRE luciferase reporter gene, together with a PLSCR1 expression construct or an empty vector (Fig 6D). We found that the overexpression of PLSCR1 did not increase the expression of the ISRE luciferase reporter gene compared with that of the PLSCR1-non-overexpressing cells. Together, these results demonstrate that the inhibitory role of PLSCR1 in influenza virus replication does not involve stimulating the IFN pathways.

### The complex formed among PLSCR1, NP and importin α inhibits importin α to form a functional nuclear import receptor complex with importin β

**Fig 7:**
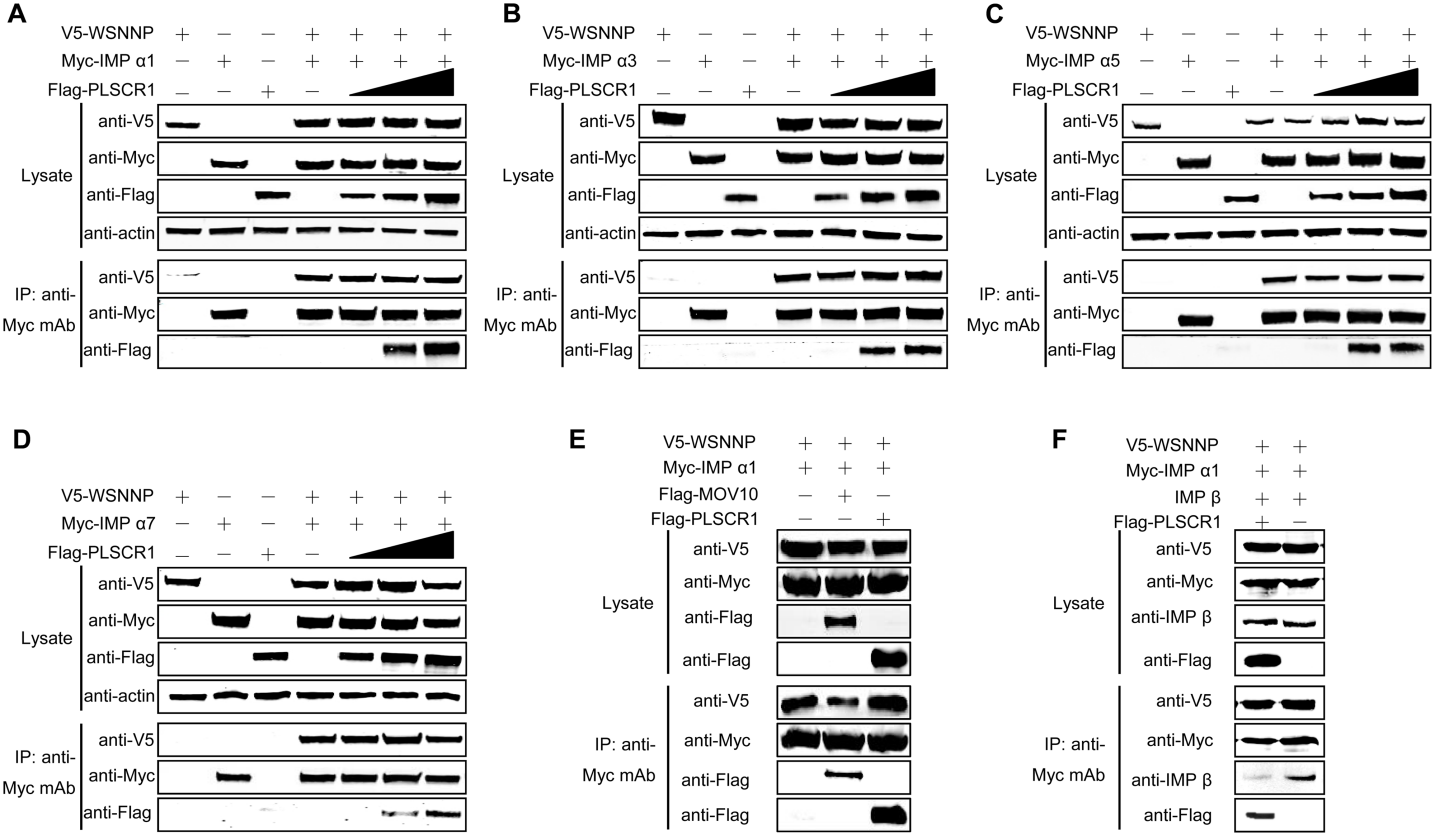
The formation of a complex comprising PLSCR1, NP, and importin α inhibits the incorporation of importin β into the complex. (A–D) PLSCR1 formed a complex with NP and different members of the importin α family: importin α1 (A), importin α3 (B), importin α5 (C), and importin α7 (D). HEK293T cells were transfected with plasmids expressing V5-WSNNP and Myc-tagged importin α proteins, together with gradual increasing amounts (0–0.6 μg) of Flag-PLSCR1. The cell lysates were immunoprecipitated with a mouse anti-Myc mAb, and the bound proteins were detected by western blotting with a rabbit anti-V5 pAb, a rabbit anti-Flag pAb, or a rabbit anti-Myc pAb to detect NP, PLSCR1, and importin α family members, respectively. (E) Validation of complex formation among NP, PLSCR1, and importin α1 by including MOV10 as a control. HEK293T cells were transfected with plasmids expressing V5-WSNNP and Myc-tagged importin α1, together with Flag-PLSCR1 or Flag-MOV10. The cell lysates were immunoprecipitated with a mouse anti-Myc mAb, and the bound proteins were detected by western blotting with a rabbit anti-V5 pAb, a rabbit anti-Flag pAb, or a rabbit anti-Myc pAb to detect NP, PLSCR1 or MOV10, and importin α1, respectively. (F) Complex formation among PLSCR1, NP, and importin α1 inhibited the incorporation of importin β into the complex. HEK293T cells were transfected with plasmids expressing V5-WSNNP, Myc-importin α1 and importin β, together with Flag-PLSCR1. The cell lysates were immunoprecipitated with a mouse anti-Myc mAb, and the bound proteins were detected by western blotting with rabbit pAb against V5, Myc or the Flag tag, or importin β.

Importin α plays an important role in the nuclear import of proteins [42]. PLSCR1 has been reported to interact with importin α [28]. We therefore attempted to determine whether the interaction between PLSCR1 and NP interferes with the complex formation between NP and importin α, thus preventing the nuclear import of NP through the classical nuclear import pathway. To this end, we transfected HEK293T cells with V5-tagged NP and Myc-tagged importin α proteins, together with gradually increasing amounts of Flag-tagged PLSCR1 construct. At 48 h post-transfection, cell lysates were immunoprecipitated with an anti-Myc mAb, followed by western blotting with rabbit pAbs against Myc, V5, or the Flag tag to detect importin α, NP, and PLSCR1, respectively. As shown in Fig 7A, V5-tagged NP was coimmunoprecipitated with Myc-tagged importin α1 when they were coexpressed, but not in the absence of Myc-tagged importin α1, indicating that NP interacts with importin α1 in mammalian cells. When increasing amounts of Flag-tagged PLSCR1 construct were co-transfected with V5-tagged NP and Myc-tagged importin α1, the expression level of PLSCR1 also gradually increased in the cell lysates. More PLSCR1 was detected in the importin α1 immunoprecipitates as the amount of transfected PLSCR1 construct increased from 0.2 to 0.6 μg. Significantly, V5-tagged NP and Flag-tagged PLSCR1 were simultaneously co-immunoprecipitated with Myc-tagged importin α1, and the amount of NP coimmunoprecipitated with importin α1 was not reduced when the amount of transfected PLSCR1 was increased. This result indicates that the presence of PLSCR1 did not affect the formation of the complex between NP and importin α1; rather, a trimeric complex of PLSCR1, NP, and importin α1 was formed. We then performed similar co-IP experiments with PLSCR1, NP, and other members of the importin α family. V5-tagged NP and Flag-tagged PLSCR1 were coimmunoprecipitated with Myc-tagged importin α3 (Fig 7B), importin α5 (Fig 7C), or importin α7 (Fig 7D), and the gradually increased expression of PLSCR1 did not reduce the interaction between NP and these members of the importin α family. To validate this finding, we included the host factor MOV10 as a control in the co-IP experiment, because MOV10 has been shown to compete with importin α to interact with NP [17]. We found that co-expression of MOV10 reduced the amount of NP coimmunoprecipitated with importin α1, indicating that MOV10 indeed inhibited the interaction between importin α and NP (Fig 7E). In contrast, the expression of PLSCR1 did not affect the binding between importin α and NP. These data clearly indicated that the NP protein bound by MOV10 was no longer bound by importin α, but the NP protein bound by PLSCR1 could still bind to importin α. Taken together, these results demonstrate that PLSCR1 forms an integrative three-subunit complex with NP and importin α.

Thus, our results demonstrated that PLSCR1 forms a complex with NP and importin α and causes cytoplasmic retention of NP. We then performed another co-IP experiment to further reveal the underlying mechanism. HEK293T cells were transfected with plasmids expressing V5-WSNNP, Myc-importin α1, importin β, or together with Flag-PLSCR1. The cell lysates were immunoprecipitated with a mouse anti-Myc mAb, and the bound proteins were detected by western blotting with rabbit pAb against V5, Myc, Flag tag, or importin β. Strikingly, PLSCR1 expression significantly reduced the amount of importin β in the immunoprecipitates (Fig 7F), indicating that the formation of the complex of PLSCR1, NP, and importin α1 blocked the access of importin β, the key mediator of the classical nuclear import pathway, to the complex, thereby inhibiting the nuclear import of NP via the classical nuclear import pathway and suppressing virus replication.

## Discussion

The vRNP complex is responsible for the transcription and replication of the influenza viral genome in the nucleus of infected cells [16, 18, 43]. In addition to the three polymerase subunits with one copy of each, most of the vRNP complex is encapsidated by the viral NP protein [44, 45]. As the most abundant protein in the virus particle, except for M1 [46], NP inevitably becomes the main target of the host defense system. In this study, we identified the host cellular protein PLSCR1 as an interacting partner of the NP protein by using yeast two-hybrid screening. We demonstrated that NP and PLSCR1 interact in both transfected and infected mammalian cells. Western blotting analysis showed that PLSCR1 did not affect the phosphorylation status of NP. Instead, we found that PLSCR1 formed a complex with NP and members of the importin α family, inhibited nuclear import of vRNP/NP, and thereby suppressed virus genome transcription and replication and negatively regulated the propagation of different influenza virus subtypes.

**Fig 8:**
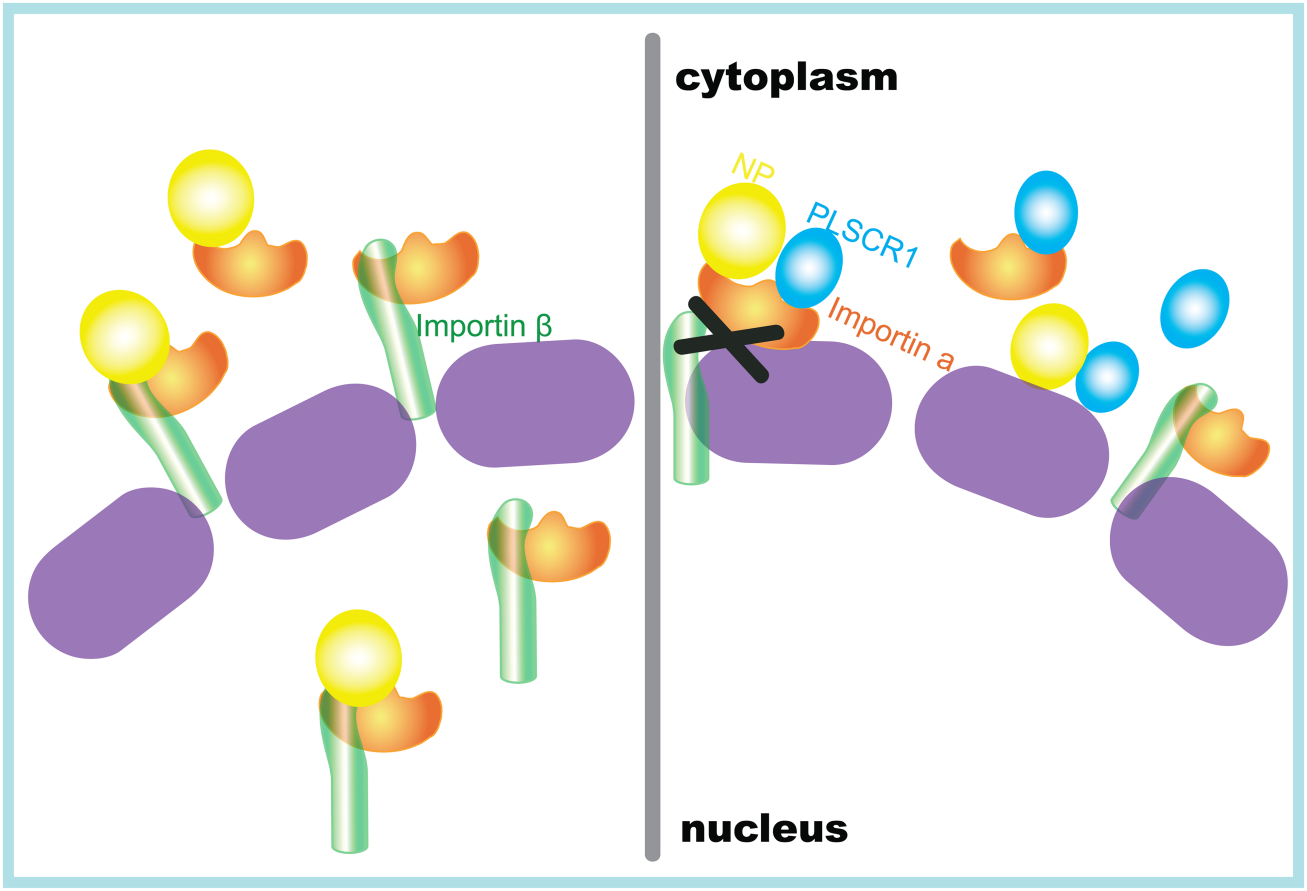
Model of PLSCR1-mediated inhibition of the nuclear import of influenza NP. In the cytoplasm, influenza virus NP, derived from newly synthesized NP or incoming vRNP, is bound by the heterodimeric import receptor, importin α/importin β, leading to its transport into the nucleus through the nuclear pore; in the presence of PLSCR1, an integrative complex among NP, PLSCR1, and importin α is formed in the cytoplasm that prevents importin α and importin β from forming a functional nuclear import receptor complex, thereby suppressing the nuclear import of NP.

The active nuclear import of the vRNP complex is mediated by an interaction between NP and importin α through the classical nuclear import pathway [10, 16, 17]. Host factors are reported to be involved in this active process. One such factor, Hsp-40, has been shown to be required for the efficient association between NP and importin α, thus promoting the nuclear localization of vRNP complex [20]. In contrast, MOV10 was found to disrupt the binding between NP and importin α, thereby causing the retention of NP in the cytoplasm and a reduction in virus replication [17]. In the present study, we found that the nuclear import of the vRNP complex was significantly retarded in virus-infected PLSCR1-overexpressing A549 cells compared with empty retrovirus-transduced control cells. Furthermore, NP clearly accumulated in the nucleus of cells that were not transfected with the PLSCR1 construct, whereas NP was predominantly retained in the cytoplasm and colocalized with PLSCR1 when PLSCR1 was significantly overexpressed by transfection. These results demonstrate that PLSCR1 inhibited the import of the NP/vRNP complex into the nucleus. Interestingly, we found that the inhibitory effect of PLSCR1 on the nuclear import of the NP/vRNP complex was not achieved by impairing the interaction between NP and importin α. Instead, NP, PLSCR1, and importin α formed a stable complex, which inhibited the interaction between importin α and importin β. Taken together, our findings favor a model in which influenza virus NP, derived from the newly synthesized NP or incoming vRNP, is bound by the heterodimeric import receptor, importin α/importin β, in the cytoplasm and is transported into the nucleus; in the presence of PLSCR1, the complex formed among NP, PLSCR1, and importin α in the cytoplasm prevents importin α from forming a functional nuclear import receptor complex with importin β, thereby suppressing the nuclear import of NP (Fig 8). We speculate that the simultaneous binding of two molecules by importin α may overload this nuclear import adaptor, or alter its structural property, thereby affecting its ability to interact with importin β.

As an IFN-inducible gene, PLSCR1 can enhance the antiviral activity of IFN by increasing the expression of IFN-stimulated genes [33]. In the presence of exogenous IFN-β, PLSCR1 has been shown to increase its antiviral activity against VSV and EMCV [33]. PLSCR1 has also been shown to inhibit HBV replication by reducing the synthesis of viral proteins, DNA replicative intermediates, and viral RNAs [36]. In addition to these indirect antiviral activities, PLSCR1 can directly bind to the Tax protein of HTLV-1 to reduce its transactivation activity by altering the subcellular distribution and homodimerization of Tax [37]. In the present study, we found that PLSCR1 directly binds to the NP protein of influenza virus and inhibits the nuclear import of NP/vRNP, thus demonstrating a new direct antiviral role for PLSCR1.

The localization of PLSCR1 is directly correlated with its function. Because it was initially identified as plasma membrane protein, its role in regulating the movement of plasma membrane phospholipids was intensively studied [22, 47, 48]. Moreover, cell surface-localized PLSCR1 can bind to the envelope proteins E1 and E2 of HCV and serve as an attachment factor for HCV entry [49]. As a result, downregulation of PLSCR1 expression inhibits HCV entry and infection. PLSCR1 can also be imported into the nucleus by the importin α/β import pathway [28], where it can bind to genomic DNA or nuclear proteins to perform different functions [29, 31, 50]. Yet, we found that PLSCR1 was localized predominantly in the cytoplasm of both PLSCR1-overexpressing and empty retrovirus-transduced control A549 cells infected with influenza viruses. The accumulation of PLSCR1 in the cytoplasm would likely enable it to efficiently participate in interactions with both importin α and viral NP, and effectively inhibit the nuclear import of vRNP/NP and virus replication.

Mutations of NP phosphorylation sites can reduce the binding affinity between NP and various members of the importin α family, resulting in the inhibition of nuclear import of NP and a reduction in virus replication [40]. In the present study, we found that the replication of influenza virus in PLSCR1-overexpressing A549 cells decreased the overall expression of viral NP protein. However, the extent of NP phosphorylation was similar between the PLSCR1-overexpressing cells and empty retrovirus-transduced control A549 cells. In addition, the replication of the NP phosphorylation mutant S9A/Y10F was further decreased in PLSCR1-overexpressing cells, thereby demonstrating an accumulative inhibitory effect on influenza virus propagation. Together, these results suggest that the effect of PLSCR1 on virus replication does not involve modulating the phosphorylation status of NP.

In summary, here we demonstrated that PLSCR1 is an interacting partner of the influenza NP protein. This interaction appears to downregulate virus replication since overexpression of PLSCR1 resulted in a significant reduction in virus titer in cell cultures of different virus subtypes, whereas siRNA knockdown or CRISPR/Cas9 knockout of PLSCR1 expression increased virus replication. Importantly, PLSCR1 inhibited the nuclear import of vRNP/NP, thus causing retardation of the virus life cycle. Moreover, we revealed that the mechanism by which PLSCR1 regulates influenza virus replication involves the formation of a complex with viral NP and importin α, which inhibits the incorporation of importin β into the complex and suppresses the nuclear import of NP. Collectively, our data suggest that PLSCR1 is an important host restriction factor against influenza virus.

## Materials and methods

### Cells and viruses

HEK293T (ATCC CRL-3216), A549 (ATCC CCL-185), and MDCK (ATCC PTA-6500) cells were cultured in DMEM (Life Technologies, Grand Island, NY) containing 10% fetal bovine serum (FBS, Sigma-Aldrich, St. Louis, MO), in F12K (Life Technologies) with 10% FBS, or in MEM (Life Technologies) containing 5% newborn calf serum (NCS; Sigma-Aldrich), respectively. All media were supplemented with 100 units/ml penicillin and 100 μg/ml streptomycin (Life Technologies). All cells were cultured at 37°C with 5% CO_2_. A/Anhui/2/2005 (AH05, H5N1) and A/Anhui/1/2013 (AH13, H7N9) were grown in 10-day-old embryonated chicken eggs. A/WSN/33 (WSN, H1N1) and A/Fuzhou/1/2009 (FZ09, H1N1) were propagated in MDCK cells cultured in MEM containing 0.3% bovine serum albumin (BSA, Sigma-Aldrich) and 0.5 μg/ml L-1-tosylamide-2-phenylmethyl chloromethyl ketone (TPCK)-treated trypsin (Worthington, Lakewood, NJ).

All experiments with H5N1 and H7N9 viruses were conducted within the enhanced animal biosafety level 3 (ABSL3+) facility in the Harbin Veterinary Research Institute (HVRI) of the Chinese Academy of Agricultural Sciences (CAAS), which is approved for such use by the Ministry of Agriculture of China and the China National Accreditation Service for Conformity Assessment.

### Yeast two-hybrid assay

The yeast two-hybrid screen for protein-protein interactions was performed by using the matchmaker yeast two-hybrid system (Clontech, Mountain View, CA) as previously described [38]. NP of AH05 (H5N1) was constructed in pGBKT7, fused to the C-terminus of the GAL4-binding domain (BD), and used as bait. cDNAs prepared from a mixed human cell culture comprising A549, HEK293T, THP-1 (ATCC TIB-202), and U251 (Type Culture Collection of the Chinese Academy of Sciences, Shanghai, China) were cloned into pGADT7, fused to the GAL4-activation domain (AD), and used as prey. The yeast strain Y2HGold was transformed with the pGBKT7-AH05NP bait by using lithium acetate, and was then mated with the Y187 strain transformed with the pGADT7-based cDNA library. Transformants were selected on plates with synthetically defined medium lacking adenine, histidine, leucine, and tryptophan (SD/–Ade/–His/–Leu/–Trp) (quadruple dropout medium, QDO). The recovered colonies were grown on QDO plates containing 5-bromo-4-chloro-3-indolyl-α-d-galactopyranoside (X-α-Gal) and aureobasidin A (AbA) (SD/–Ade/–His/–Leu/–Trp/X-a-Gal/AbA, QDO/X/A). Blue colonies were selected and cultured in medium lacking leucine and tryptophan (SD/−Leu/−Trp) (double dropout medium, DDO). Plasmids were purified and sequenced to identify the potential cellular interactants with NP. To eliminate false-positive interactions, the bait and prey plasmids were cotransformed into the Y2HGold strain. Cotransformation of pGADT7-T (AD-T) with pGBKT7-p53 (BD-p53) into Y2HGold served as a positive control, and cotransformation of AD-T with pGBKT7-Lamin (BD-Lam) served as a negative control.

### Plasmids

Human PLSCR1, importin β and the open reading frames (ORFs) of PB2, PB1, PA, and NP derived from WSN virus were cloned into the mammalian expression vector pCAGGS (a gift from Dr. Yoshihiro Kawaoka, University of Wisconsin-Madison). GST-tagged PLSCR1 and WSN NP were constructed in pCAGGS with a GST tag at the N-terminus. Plasmids pCAGGS-V5-WSNNP, pCAGGS-Flag-PLSCR1 and pCAGGS-Flag-MOV10 were generated by inserting the ORF of WSN NP, PLSCR1 and MOV10 fused with the V5 or Flag tag sequence at the N-terminus into the pCAGGS vector. Truncation mutants of GST-tagged WSN NP were generated by using a PCR approach and were cloned into the pCAGGS vector. pQCXIN-PLSCR1 was constructed by inserting the PLSCR1 ORF into the pQCXIN vector (Clontech). WSN NP S9A/Y10F, containing two mutations in the full-length NP gene, was generated by using a Fast Mutagenesis System (Transgen, Beijing, China) and was cloned into the pHH21 vector (a gift from Dr. Yoshihiro Kawaoka, University of Wisconsin-Madison). pHH21-SC09NS F-Luc, used to produce negative-sense RNA containing 176 bases of the 3’ end of the NS vRNA derived from A/Sichuan/1/2009 (SC09, H1N1), a firefly luciferase, a stop codon (TAA), and 179 bases of the 5’ end of SC09NS vRNA, was constructed by using the PCR method. The full-length ORFs of human importin α1, importin α3, importin α5, and importin α7 were cloned into pCAGGS with a Myc tag at the N-terminus. All plasmid constructs were confirmed by sequencing.

### Antibodies

Mouse anti-NP monoclonal antibody (mAb) and rabbit anti-NP polyclonal antibody (pAb) were prepared in our laboratory by using conventional methods. The following primary antibodies were purchased from commercial resources: rabbit anti-V5 pAb (AB3792, Merck Millipore, Darmstadt, Germany); rabbit anti-GAPDH pAb (10494-1-AP), rabbit anti-importin β pAb (10077-1-AP), rabbit anti-LaminB1 pAb (12987-1-AP), rabbit anti-Mx1 pAb (13750-1-AP) and rabbit anti-PLSCR1 pAb (11582-1-AP) from Proteintech (Wuhan, China); mouse anti-actin mAb (sc-47778), mouse anti-p-Ser mAb (sc-81514) and mouse anti-p-Tyr mAb (sc-508) from Santa Cruz (Dallas, TX); mouse anti-Flag mAb (F3165), mouse anti-Myc mAb (M4439), mouse anti-V5 mAb (V8012), rabbit anti-Flag pAb (F7425) and rabbit anti-Myc pAb (C3965) from Sigma-Aldrich. The secondary antibodies used in the western blotting were DyLight 800 goat anti-mouse IgG (H+L) (072-07-18-06) and DyLight 800 goat anti-rabbit IgG (H+L) (072-07-15-06), purchased from KPL (Gaithersburg, MD); the secondary antibodies used in the confocal microscopy were Alexa Fluor 488 donkey anti-rabbit IgG (H+L) (A21206) and Alexa Fluor 633 goat anti-mouse IgG (H+L) (A21050) obtained from Life Technologies.

### Co-immunoprecipitation assay

To examine the interaction of proteins in transfected cells, HEK293T cells were transfected with the indicated plasmids by using the Lipofectamine LTX and Plus Reagents (Invitrogen, Carlsbad, CA). To determine the interaction of proteins during natural viral infection, A549 cells were mock infected with PBS or infected with WSN virus at an MOI of 5. Cell lysates were prepared at 48 h post-transfection for transfected HEK293T cells or at 6 h p.i. for infected A549 cells. Briefly, the cells were washed twice with cold PBS and lysed with IP buffer (25 mM Tris-HCl pH 7.4, 150 mM NaCl, 1% NP-40, 1 mM EDTA, 5% glycerol; Pierce, Rockford, IL) containing complete protease inhibitor cocktail (Roche Diagnostics GmbH, Mannheim, Germany) for 30 min on ice and then centrifuged at 12,000 rpm at 4°C for 10 min. The supernatants were mixed with the respective primary antibodies, rocked overnight at 4°C, mixed with Protein G-Agarose beads (Roche) and rock for 6–8 h. The beads were washed four times with wash buffer (25 mM Tris-HCl pH 7.4, 150 mM NaCl, 1 mM PMSF). The bound proteins were then boiled in 2 × SDS sample buffer, separated by 12% sodium dodecyl sulfate-polyacrylamide gel electrophoresis (SDS-PAGE), and detected by western blotting.

### GST pull-down

HEK293T cells grown in 10-cm dishes were individually transfected with 10 μg of each plasmid (pCAGGS, pCAGGS-GST, pCAGGS-GST-PLSCR1, pCAGGS-NP, pCAGGS-GST-NP, or pCAGGS-PLSCR1) by using the Lipofectamine LTX and Plus Reagents. At 48 h post-transfection, cells were solubilized with 0.8 ml of IP buffer. Then, 300 μl of the cleared lysates from cells transfected with pCAGGS-GST, pCAGGS-GST-PLSCR1, or pCAGGS-GST-NP was mixed with 40 μl of Glutathione Sepharose 4 Fast Flow (GE Healthcare, Pittsburgh, PA) and rocked for 1 h at 4°C. After three washes with wash buffer, 300 μl of the cleared lysates from cells transfected with non-GST expressing constructs (i.e., pCAGGS, pCAGGS-NP, or pCAGGS-PLSCR1) was added and incubated for 2 h at 4°C. After three washes, the bound proteins were separated by SDS-PAGE. GST, GST-PLSCR1, or GST-WSNNP proteins in the eluates were detected by Coomassie blue (CB) staining, and non-GST tagged NP and PLSCR1 proteins were detected by western blotting.

### Western blotting

Protein samples fractionated by SDS-PAGE were transferred onto nitrocellulose membranes (GE Healthcare). Membranes blocked with 5% skim milk in PBST were incubated overnight at 4°C with appropriately diluted primary antibody in PBST containing 2% BSA. After incubation with DyLight 800 goat anti-mouse IgG (H+L) and DyLight 800 goat anti-rabbit IgG (H+L), blots were visualized by using an Odyssey infrared imaging system (Li-Cor BioSciences, Lincoln, NE).

### Establishment of a stable A549 cell line overexpressing PLSCR1 and virus infection

The AmphoPack-293 packaging cell line (631505, Clontech) cultured in 10-cm dishes was transfected with either retroviral construct pQCXIN-PLSCR1 or with the empty pQCXIN vector by using Lipofectamine LTX and Plus Reagents. At 48 h post-transfection, viral supernatants from the transfectants were collected and used to transduce A549 cells cultured in 6-well plates. Forty-eight hours later, the transduction was repeated to enrich for transductants. The confluent transduced cells were split and cultured in medium supplemented with 1000 μg/ml G418 for selection. The surviving cells were individually cloned in 96-well plates, propagated, and examined for PLSCR1 overexpression by quantitative reverse-transcription PCR (RT-qPCR) and western blotting. To study the effect of PLSCR1 overexpression on influenza virus replication, we used WSN (H1N1), AH05 (H5N1), AH13 (H7N9) or FZ09 (H1N1) to infect the PLSCR1-overexpressing cells or the empty retrovirus-transduced control A549 cells at an MOI of 0.1. Supernatants were collected at the indicated timepoints after infection and virus titers were determined by means of plaque assays on MDCK cells [38].

### siRNA knockdown and virus infection

siRNA targeting PLSCR1 (5’-GCGGAAGAUACUGAUUGCU-3’) or scrambled siRNA (Genepharma, Shanghai, China) at a concentration of 30 nM was transfected into A549 cells seeded in 12-well plates by using the Lipofectamine RNAiMAX transfection reagent (Invitrogen). Forty-eight hours later, the knockdown efficiency was checked by means of RT-qPCR and western blotting. To study the effect of PLSCR1 knockdown on the growth of influenza virus, the WSN virus was used to infect siRNA-treated A549 cells at an MOI of 0.1. Supernatants were collected at 24 and 48 h post-infection (p.i.), and the virus titers were determined by means of plaque assays on MDCK cells.

### Cell viability assay

Cell viability was determined by using the CellTiter-Glo kit (Promega, Madison, WI) as described previously [38]. Briefly, A549 cells seeded in opaque-walled 96-well plates were transfected with siRNA targeting PLSCR1 or with scrambled siRNA at a concentration of 30 nM. At 48 h post-transfection, 100 μl of CellTiter-Glo reagent was added directly into each well and incubated with the cells for 10 min on a shaker to induce cell lysis. The luminescence was measured with a GloMax 96 Microplate Luminometer (Promega).

### Generation of PLSCR1-KO HEK293T cells and virus infection

PLSCR1-KO HEK293T cells were established using the CRISPR/Cas9 system. The PLSCR1 gene target sequence, 5’–CAGGATATAGTGGCTACCCT– 3’ (to target exon 4), was inserted into the guide RNA (gRNA) expression cassette of the pX330 vector [51], which also contains an expression cassette of Cas9. Six micrograms of the pX330 plasmid containing the PLSCR1 target sequence was then transfected into HEK293T cells with TransIT-LT1 (Mirus, Madison, WI). The transfected cells were trypsinized 24 h later into single cells, which were diluted and inoculated into 96-well plates for colony formation. Each colony was individually propagated into 24 well-plates, and the knockout of PLSCR1 expression was confirmed by western blotting. The PLSCR1-KO HEK293T or control cells were infected with WSN virus at an MOI of 0.1. Supernatants were collected at 24 and 48 h p.i., and virus titers were determined by means of plaque assays on MDCK cells.

### Confocal microscopy

A549 cells seeded in glass-bottom dishes were transfected with the indicated plasmids by using the Lipofectamine LTX and Plus Reagents. PLSCR1-overexpressing cells or empty retrovirus-transduced control A549 cells were infected with WSN virus at an MOI of 5. At 20 h post-transfection or 4, 6, 8, 10, and 12 h p.i., cells were fixed with 4% paraformaldehyde (PFA) in PBS for 1 h, and permeabilized with 0.5% Triton X-100 in PBS for 30 min. The permeabilized cells were blocked with 5% BSA in PBS for 1 h, and then incubated with primary antibodies (mouse anti-NP mAb, 1:500; rabbit anti-PLSCR1 pAb, 1:1000) for 2 h. The cells were washed three times with PBS and incubated with the secondary antibodies (Alexa Fluor 488 donkey anti-rabbit IgG (H+L), 1:10000; and Alexa Fluor 633 goat anti-mouse IgG (H+L), 1:10000) for 1 h. After four washes, the cells were incubated with DAPI (4’,6-diamidino-2-phenylindole, Thermo Fisher Scientific, Waltham, MA) for 15 min to stain the nuclei. Images were acquired by using the Leica SP2 confocal system (Leica Microsystems, Wetzlar, Germany).

### Dual-luciferase reporter assay

HEK293T cells were treated with either siRNA specifically targeting PLSCR1 or with scrambled siRNA (30 nM) for 48 h, and were then cotransfected with the four protein expression plasmids of the RNP complex from WSN (pCAGGS-PB2, pCAGGS-PB1, pCAGGS-PA, and pCAGGS-NP; 1 μg of each), the construct pHH21-SC09NS F-Luc (0.1 μg), and an internal control pRL-TK (0.1 μg). At 48 h post-transfection, cell lysates were prepared by using the dual luciferase reporter assay system (Promega), and the luciferase activities were measured on a GloMax 96 microplate luminometer (Promega).

HEK293T cells grown in 24-well plates were transfected with the ISRE-Luc reporter plasmid (0.25 μg), pRL-TK control plasmid (0.02 μg), and the pCAGGS-PLSCR1 or empty pCAGGS plasmid (0.25 μg) for 20 h. The luciferase activity of the transfected cells was determined by using the dual-luciferase reporter assay.

### IFN-α treatment and Mx1 expression

PLSCR1-overexpressing cells or empty retrovirus-transduced control A549 cells grown in 12-well plates were left untreated or treated with 100 U/mL of IFN-α (Sigma-Aldrich) for 24 h. Cell lysates were then prepared and subjected to western blotting with a rabbit anti-Mx1 pAb to determine the expression level of Mx1 protein.

### Generation of an influenza mutant

The mutant WSN virus WSN NP S9A/Y10F, which possesses two mutations in the viral NP protein, was generated by use of reverse genetics as described previously (34). Briefly, the eight plasmids for the synthesis of viral RNA (vRNA) and the four supporting plasmids to express the PB2, PB1, PA, and NP proteins were transfected into HEK293T cells with the Lipofectamine LTX and Plus Reagents. At 48 h post-transfection, the transfection supernatant was harvested and used to infect MDCK cells to produce stock viruses. To ensure that the mutant virus contained the desired mutation, vRNA was extracted from the stock viruses using a QIAmp viral RNA mini kit (QIAGEN, Valencia, CA), reverse transcribed into cDNA with Superscript III reverse transcriptase (Invitrogen), and amplified by PCR with gene-specific primers. The complete NP segment was sequenced by using an ABI 3500xL genetic analyzer (Applied Biosystems, Carlsbad, CA).

### Growth of the mutant virus in PLSCR1-overexpressing cells and control A549 cells

The PLSCR1-overexpressing or empty retrovirus-transduced control A549 cells grown in 12-well plates were infected with wild-type WSN virus or the NP mutant, WSN NP S9A/Y10F, at an MOI of 0.1. The cells were incubated with F-12K medium containing 0.3% BSA at 37°C. Virus-containing supernatant was harvested at the indicated timepoints and was subjected to plaque assays on MDCK cells to determine the virus titer.

### Quantification of PLSCR1 mRNA and viral RNA species

To quantify the level of PLSCR1 mRNA, total RNA was extracted from PLSCR1-overexpressing A549 cells or siRNA-treated A549 cells at 48 h post-transfection by using an RNeasy kit (QIAGEN). The first-strand cDNA was generated with oligo(dT) primer using Superscript III reverse transcriptase. Real-time PCR was conducted using SYBR premix Ex Taq II (TaKaRa, Dalian, China) and 0.4 μM PLSCR1 primers according to the manufacturer’s instructions. Relative RNA quantities were determined by using the comparative cycle-threshold method, with cellular GAPDH serving as the endogenous reference and empty retrovirus-transduced A549 control cells or scrambled siRNA-treated cells serving as the control.

The PLSCR1-overexpressing or empty retrovirus-transduced control A549 cells grown in 6-well plates were infected with WSN virus at an MOI of 5. Total RNA was extracted by using an RNeasy kit at 6 h and 10 h p.i. Relative quantities of viral NP genomic RNA (vRNA), complementary RNA (cRNA) and mRNA were determined by qRT-PCR as described previously [52]. Relative RNA quantities were determined with GAPDH serving as the endogenous reference.

### Cell fractionation

The PLSCR1-overexpressing or empty retrovirus-transduced control A549 cells grown in 6-well plates were infected with WSN virus at an MOI of 5. At 6 h p.i., the cells were separated into nuclear and cytoplasmic fractions by using NE-PER Nuclear and Cytoplasmic Extraction Reagents (Pierce) according to the manufacturer’s procedure. The amount of NP and PLSCR1 in each fraction was determined by western blotting with a rabbit anti-NP pAb and a rabbit anti-PLSCR1 pAb, respectively. LaminB1 and GAPDH, nuclear and cytoplasmic fraction markers, respectively, were detected by western blotting with a rabbit anti-GAPDH pAb and a rabbit anti-LaminB1 pAb, respectively.

In another experiment, PLSCR1-overexpressing or control A549 cells grown in 6-well plates were pretreated with 50 μg/mL CHX (Sigma-Aldrich) for 1 h, and then infected with WSN virus at an MOI of 5. The virus-infected cells were maintained in culture medium containing CHX for 2 h, and were then subjected to cell fractionation and western blotting as described above.

### Statistical analysis

Unless otherwise indicated, all experiments were performed at least three times; data from representative experiments are shown. Data were statistically analyzed by using the Student’s t test. A mean difference was considered statistically significant if the *P* value was < 0.05.
