# Correction: Phospholipid scramblase 1 interacts with influenza A virus NP, impairing its nuclear import and thereby suppressing virus replication

**DOI:** 10.1371/journal.ppat.1012035

**Published:** 2024-02-23

**Authors:** Weiyu Luo, Jie Zhang, Libin Liang, Guangwen Wang, Qibing Li, Pengyang Zhu, Yuan Zhou, Junping Li, Yuhui Zhao, Nan Sun, Shanyu Huang, Chenchen Zhou, Yu Chang, Pengfei Cui, Pucheng Chen, Yongping Jiang, Guohua Deng, Zhigao Bu, Chengjun Li, Li Jiang, Hualan Chen

The following panels in Fig 2B [1] intentionally present the same experimental conditions and data as lanes 1–3 of the panels of the same names in Fig 2E, and the figure legend has been updated to declare the reuse of data:

- Lysate anti-Flag
- Lysate anti-V5
- IP with anti-Flag mAb anti-Flag
- IP with anti-Flag mAb anti-V5

In Fig 3G, an incorrect image was used for the A549_PLSCR1 anti-actin panel resulting in lanes 4 and 5 overlapping with lanes 1 and 2 of the A549_Control anti-actin panel. The A549_PLSCR1 anti-actin panel is corrected in Fig 3 with this notice.

In Fig 7C, an incorrect image was used for the Lysate anti-actin panel and the Lysate anti-V5 panel was erroneously cropped to present eight lanes instead of seven. These errors are resolved in Fig 7 provided with this notice.

In the updated versions of Figs 2, 3, and 7 provided with this notice, all western blot panels are presented with the original background without adjustment to contrast, brightness, or aspect ratio. Original unadjusted images underlying all western blot images in Figs 2–7 are provided in S1 File.

In addition, the article’s Data Availability statement is updated to state that the original underlying data to support all results in the article and Supporting Information files are available from the corresponding author.

The authors apologize for the errors in the published article.

**Fig 2:**
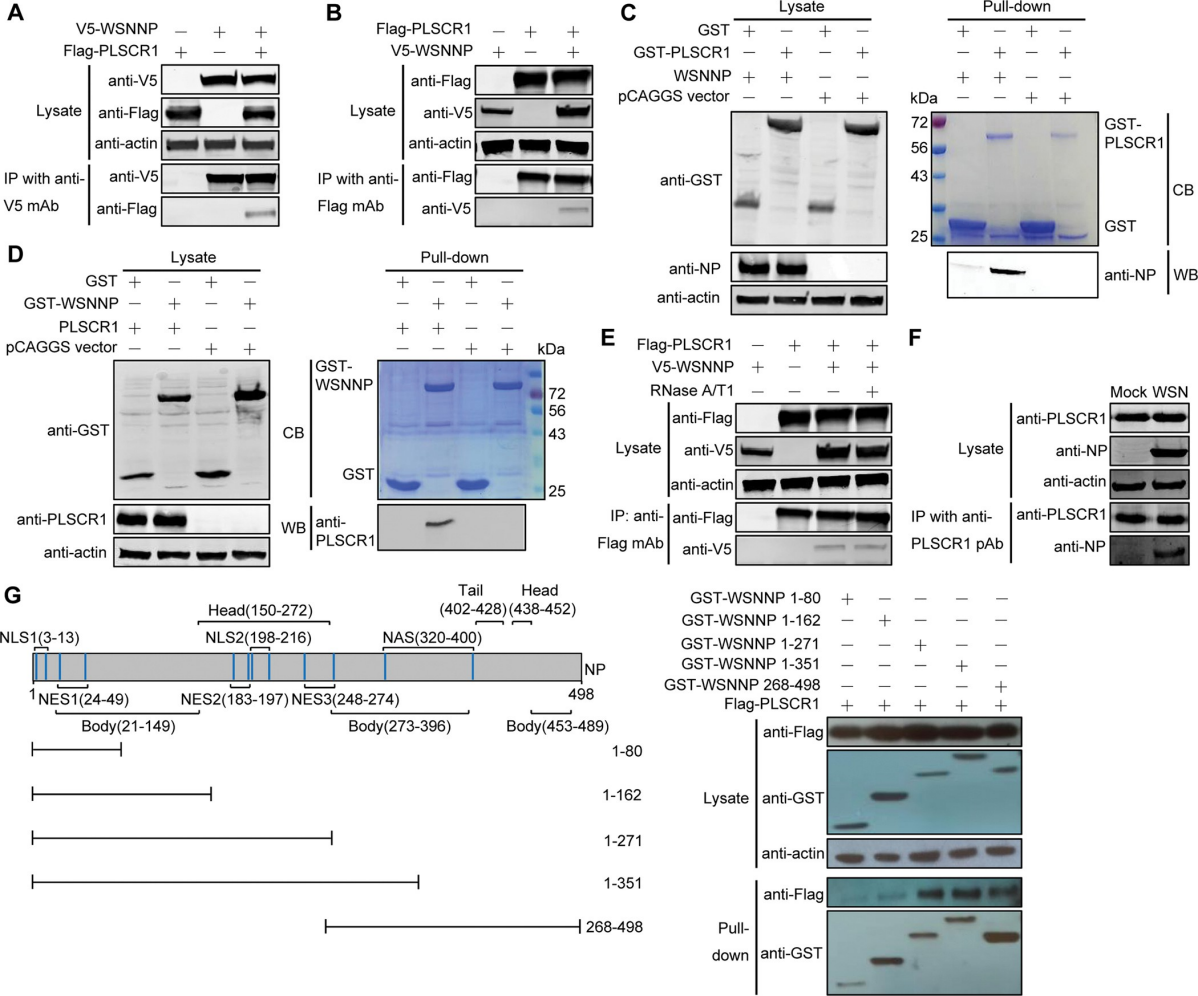
NP interacts with PLSCR1 in mammalian cells. (A, B) Co-IP assay of V5-NP and Flag-PLSCR1 in HEK293T cells. HEK293T cells were transfected individually or in combination with plasmids expressing V5-WSNNP and Flag-PLSCR1. Cell lysates were immunoprecipitated with a mouse anti-V5 mAb (A) or a mouse anti-Flag mAb (B) and were subjected to western blotting with a rabbit anti-V5 pAb or a rabbit anti-Flag pAb to reveal the presence of NP and PLSCR1, respectively. (C, D) GST pull-down assay of NP and PLSCR1. Lysates of HEK293T cells transfected with the GST or GST-PLSCR1 construct were incubated with Glutathione Sepharose 4 Fast Flow and then mixed with lysates from cells transfected with pCAGGS or pCAGGS-WSNNP (C); HEK293T cell lysates containing exogenously expressed GST or GST-WSNNP were incubated with Glutathione Sepharose 4 Fast Flow and then mixed with lysates from cells transfected with pCAGGS or pCAGGS-PLSCR1 (D). After washing away the unbound proteins, equal volumes of proteins bound to the beads and the original cell lysates (5% of the input) were examined by western blotting using a rabbit anti-NP pAb, a rabbit anti-GST pAb, or a rabbit anti-PLSCR1 pAb. GST, GST-PLSCR1, or GST-WSNNP proteins in the eluates were detected by Coomassie blue (CB) staining. (E) The NP-PLSCR1 interaction does not rely on RNA binding. HEK293T cells were transfected individually or in combination with plasmids expressing V5-WSNNP and Flag-PLSCR1. Cell lysates treated with RNase A/T1 or left untreated were immunoprecipitated with a mouse anti-Flag mAb and were subjected to western blotting with a rabbit anti-V5 pAb or a rabbit anti-Flag pAb to reveal the presence of NP and PLSCR1, respectively. (F) PLSCR1 interacts with NP during natural viral infection. Confluent A549 cells grown in 6-well plates were mock infected with PBS or infected with WSN virus at an MOI of 5. At 6 h p.i., cell lysates were immunoprecipitated with a rabbit anti-PLSCR1 pAb and were subjected to western blotting with a mouse anti-NP mAb or a rabbit anti-PLSCR1 pAb to detect NP and PLSCR1, respectively. (G) Mapping of the PLSCR1-interacting domain within NP. Schematic presentation of influenza NP showing the different domains as well as the truncation mutants made in this study is on the left side. The interaction between PLSCR1 and the NP truncation mutants is shown on the right side. Lysates of HEK293T cells were pulled down with glutathione magnetic beads. The bound proteins were subjected to western blotting with a rabbit anti-Flag pAb or a rabbit anti-GST pAb to reveal the presence of PLSCR1 and NP, respectively. NES, nuclear export signal; NAS, nuclear accumulation signal. The Lysate anti-Flag, Lysate anti-V5, IP with anti-Flag mAb anti-Flag and IP with anti-Flag mAb anti-V5 panels in Figs 2B and 2E (lanes 1–3) present the same data.

**Fig 3:**
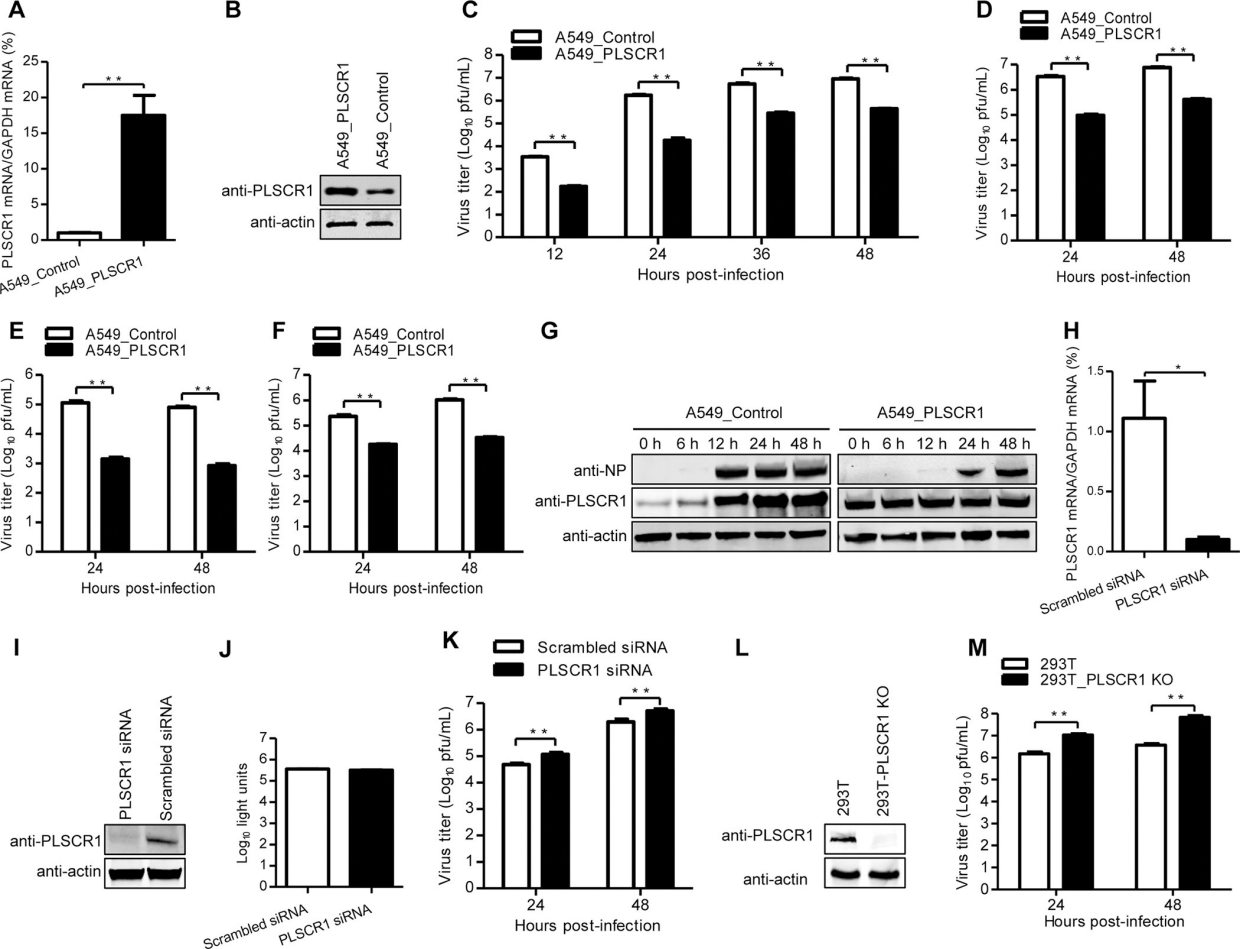
PLSCR1 negatively regulates influenza virus replication. (A, B) Establishment of an A549 cell line stably overexpressing PLSCR1. The stable overexpression of PLSCR1 was confirmed by quantitative reverse-transcription PCR (RT-qPCR) (A) and western blotting with a rabbit anti-PLSCR1 pAb (B) in comparison with the A549 control cell line transduced with an empty retrovirus. **, *P* < 0.01. (C, D, E, F) Virus replication in PLSCR1-overexpressing A549 cells. The PLSCR1-overexpressing or empty retrovirus-transduced control A549 cells were infected with WSN (H1N1) (C), AH05 (H5N1) (D), AH13 (H7N9) (E) or FZ09 (H1N1) (F) at an MOI of 0.1. Supernatants were collected at the indicated timepoints, and virus titers were determined by means of plaque assays on MDCK cells. **, *P* < 0.01. (G) Expression of PLSCR1 and NP in virus-infected cells. The PLSCR1-overexpressing or empty retrovirus-transduced control A549 cells were infected with WSN virus at an MOI of 0.1. Whole cell lysates were collected at the indicated timepoints and subjected to western blotting with a rabbit anti-PLSCR1 pAb or a rabbit anti-NP pAb. (H, I) siRNA knockdown of PLSCR1 in A549 cells. A549 cells were transfected with siRNA targeting PLSCR1 or with scrambled siRNA for 48 h. Whole cell lysates were then collected and analyzed by RT-qPCR (H) or western blotting with a rabbit anti-PLSCR1 pAb (I). *, *P* < 0.05. (J) Cell viability of siRNA-treated A549 cells was measured by using a CellTiter-Glo assay. The data are presented as means ± standard deviations (SD) for triplicate transfections. (K) Virus replication in siRNA-treated A549 cells. Cells transfected with siRNA were infected with WSN virus at an MOI of 0.1. Supernatants were collected at 24 and 48 h p.i. and titrated for infectious virus by means of plaque assays on MDCK cells. **, P < 0.01. (L) Generation of PLSCR1-KO HEK293T cells. PLSCR1-KO cells were generated by using the CRISPR/Cas9 system. PLSCR1 knockout was confirmed by western blotting with a rabbit anti-PLSCR1 pAb. (M) Virus replication in PLSCR1-KO HEK293T cells. PLSCR1-KO HEK293T or control cells were infected with WSN virus at an MOI of 0.1. Supernatants were collected at 24 and 48 h p.i., and virus titers were determined by means of plaque assays on MDCK cells. **, *P* < 0.01.

**Fig 7:**
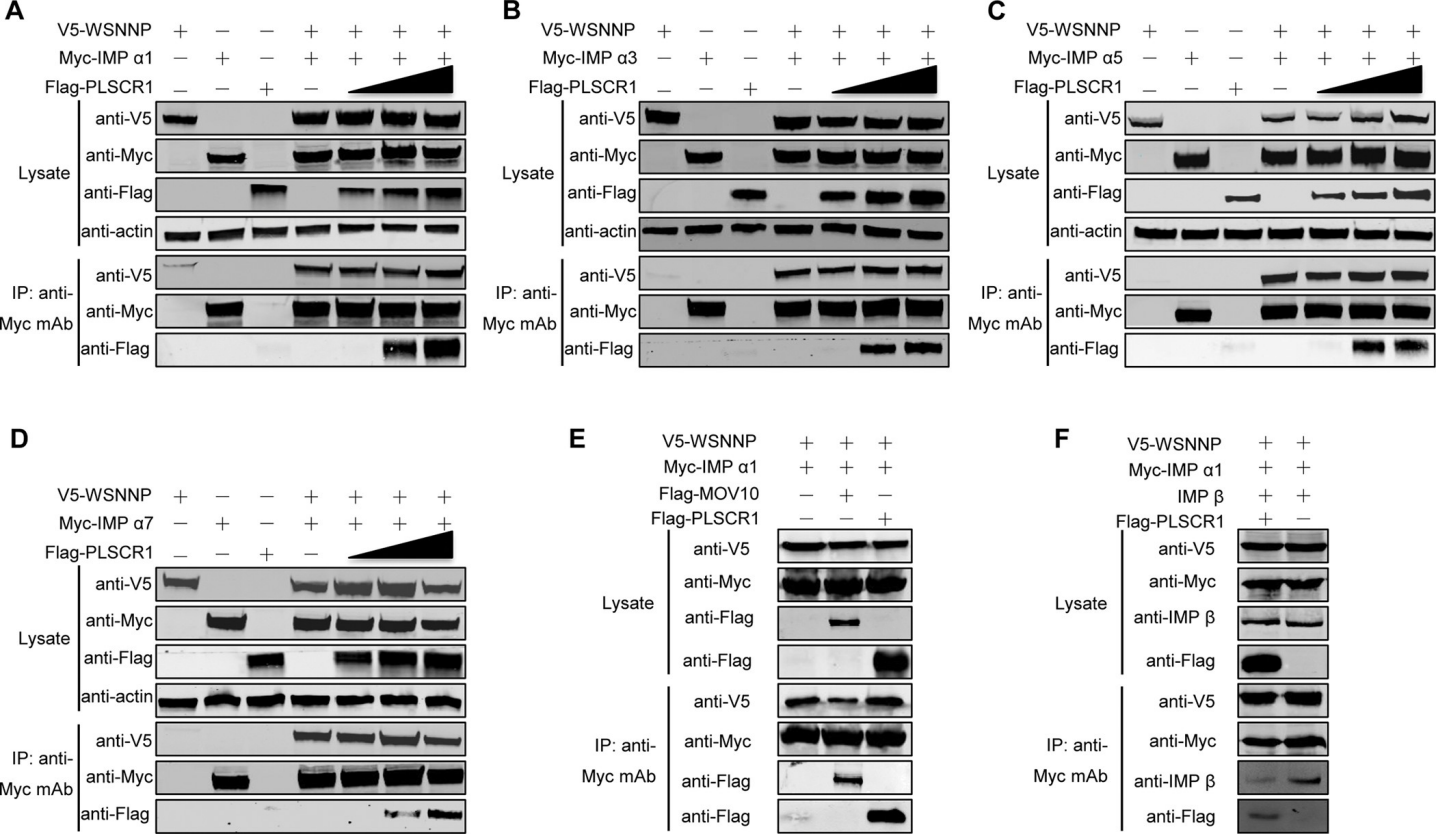
The formation of a complex comprising PLSCR1, NP, and importin α inhibits the incorporation of importin β into the complex. (A–D) PLSCR1 formed a complex with NP and different members of the importin α family: importin α1 (A), importin α3 (B), importin α5 (C), and importin α7 (D). HEK293T cells were transfected with plasmids expressing V5-WSNNP and Myc-tagged importin α proteins, together with gradual increasing amounts (0–0.6 μg) of Flag-PLSCR1. The cell lysates were immunoprecipitated with a mouse anti-Myc mAb, and the bound proteins were detected by western blotting with a rabbit anti-V5 pAb, a rabbit anti-Flag pAb, or a rabbit anti-Myc pAb to detect NP, PLSCR1, and importin α family members, respectively. (E) Validation of complex formation among NP, PLSCR1, and importin α1 by including MOV10 as a control. HEK293T cells were transfected with plasmids expressing V5-WSNNP and Myc-tagged importin α1, together with Flag-PLSCR1 or Flag-MOV10. The cell lysates were immunoprecipitated with a mouse anti-Myc mAb, and the bound proteins were detected by western blotting with a rabbit anti-V5 pAb, a rabbit anti-Flag pAb, or a rabbit anti-Myc pAb to detect NP, PLSCR1 or MOV10, and importin α1, respectively. (F) Complex formation among PLSCR1, NP, and importin α1 inhibited the incorporation of importin β into the complex. HEK293T cells were transfected with plasmids expressing V5-WSNNP, Myc-importin α1 and importin β, together with Flag-PLSCR1. The cell lysates were immunoprecipitated with a mouse anti-Myc mAb, and the bound proteins were detected by western blotting with rabbit pAb against V5, Myc or the Flag tag, or importin β.

## Supporting information

S1 File Original western blot images for Figs 2–7. (ZIP)
