# Supplementary material for: Correction: Phospholipid scramblase 1 interacts with influenza A virus NP, impairing its nuclear import and thereby suppressing virus replication
Source: PLoS Pathog. 2024 Feb 23;20(2):e1012035. doi: 10.1371/journal.ppat.1012035 (PMC10889636; doi:10.1371/journal.ppat.1012035)
Supplement: S1 File — (ZIP) [file ppat.1012035.s001.zip › S1 File. Original western blot images/Annotated Western Blot Images.pdf]

Fig 2A

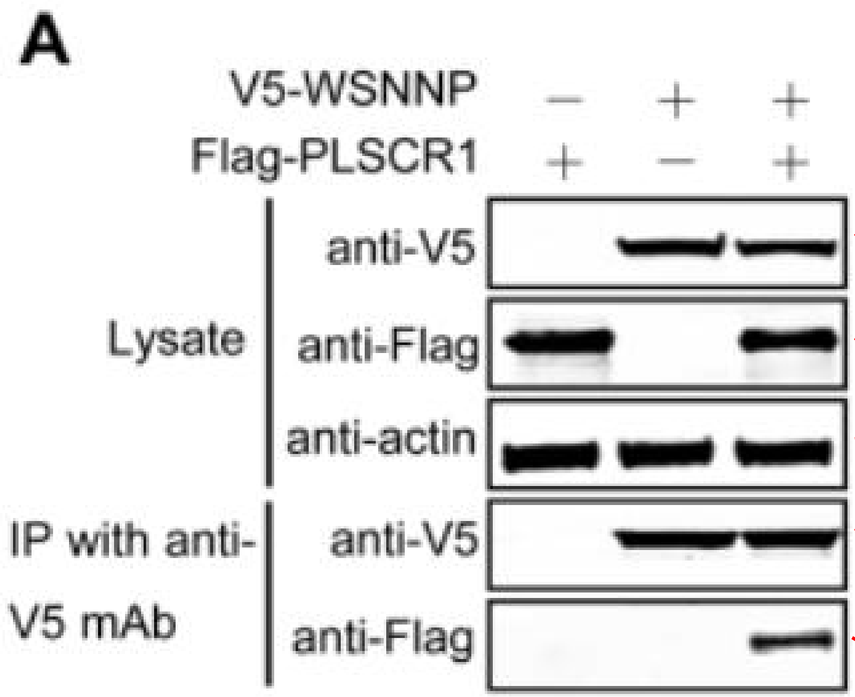
A
V5-WSNNP
Flag-PLSCR1
Lysate
anti-V5
anti-Flag
anti-actin
IP with anti-V5 mAb
anti-V5
anti-Flag

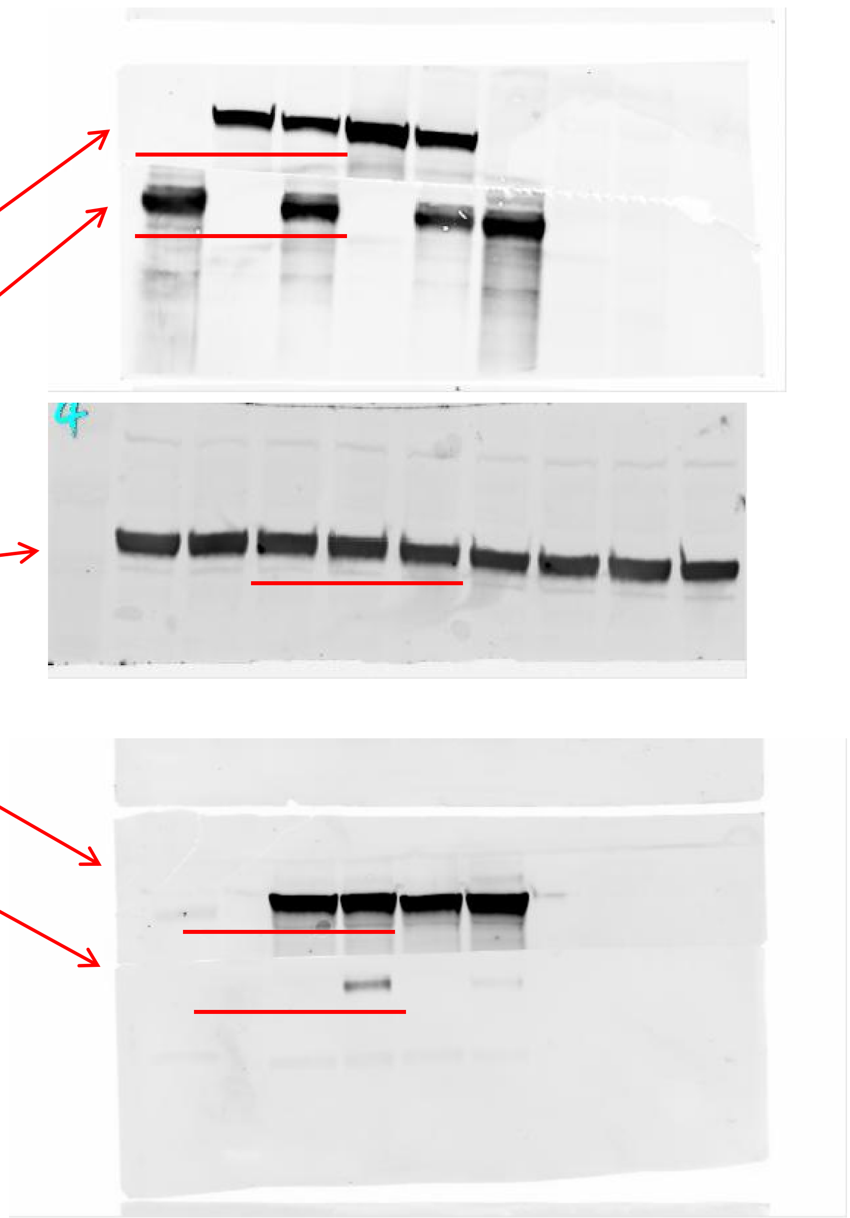

Fig 2B

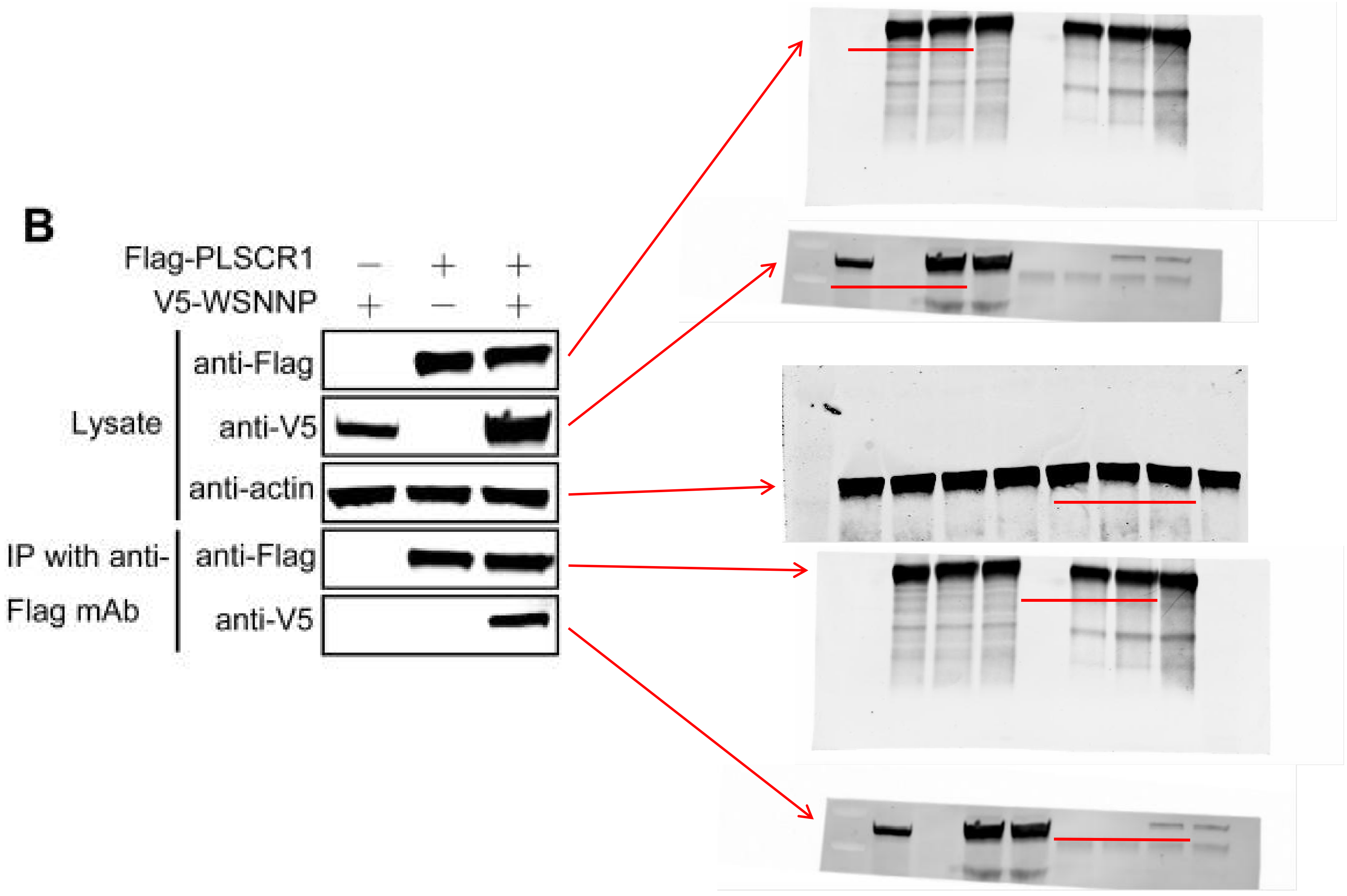
B
Flag-PLSCR1 — + +
V5-WSNNP + — +
Lysate
anti-Flag
anti-V5
anti-actin
IP with anti-Flag mAb
anti-Flag
anti-V5

Fig 2C

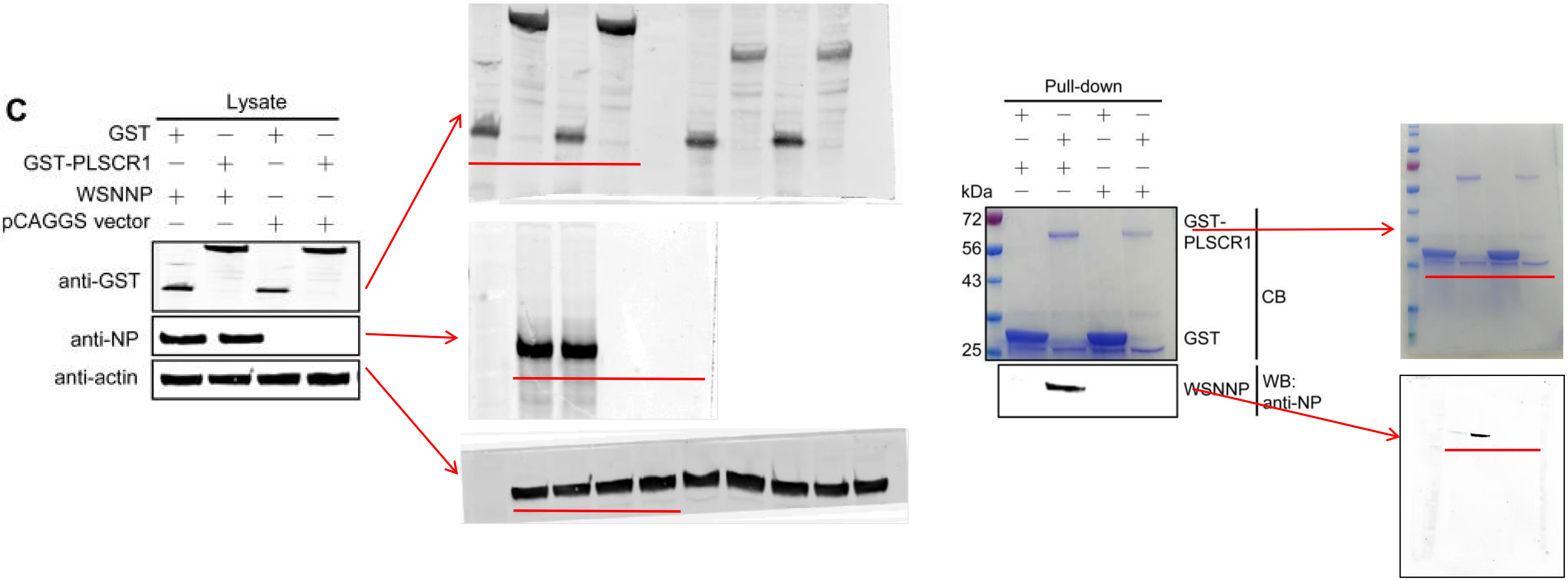
C
Lysate
GST + − + −
GST-PLSCR1 − + − +
WSNNP + + − −
pCAGGS vector − − + +
anti-GST
anti-NP
anti-actin
Pull-down
kDa
72
56
43
25
GST-
PLSCR1
CB
GST
WSNNP
WB:
anti-NP

Fig 2D

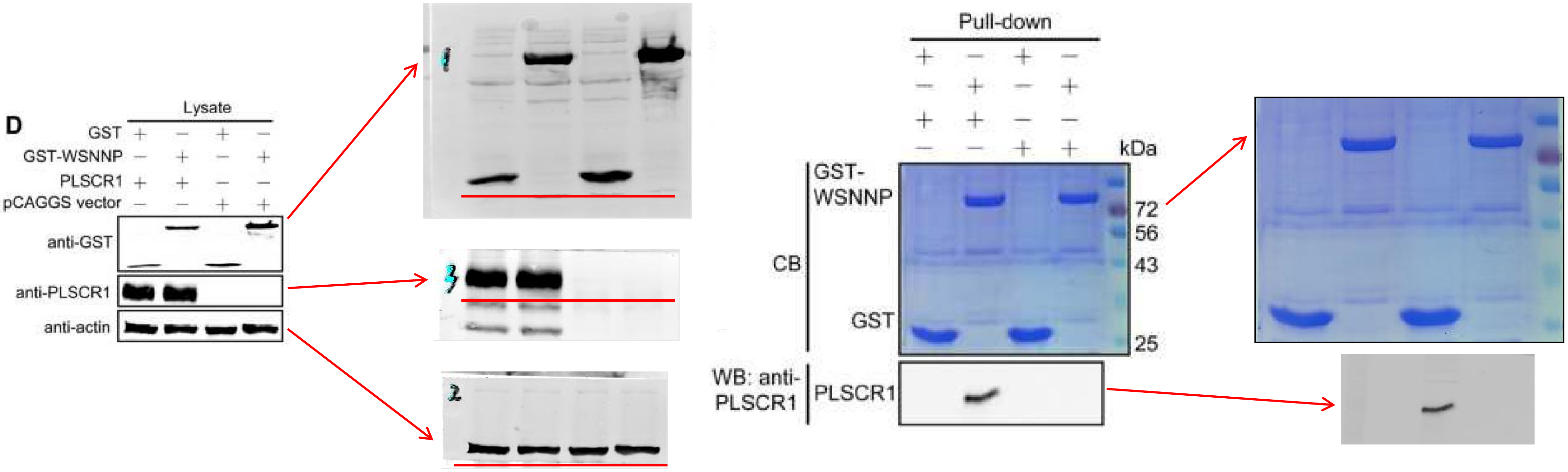
D
Lysate
GST + − + −
GST-WSNNP − + − +
PLSCR1 + + − −
pCAGGS vector − − + +
anti-GST
anti-PLSCR1
anti-actin
Pull-down
kDa
GST-
WSNNP
CB
GST
72
56
43
25
WB: anti-
PLSCR1
PLSCR1

Fig 2E

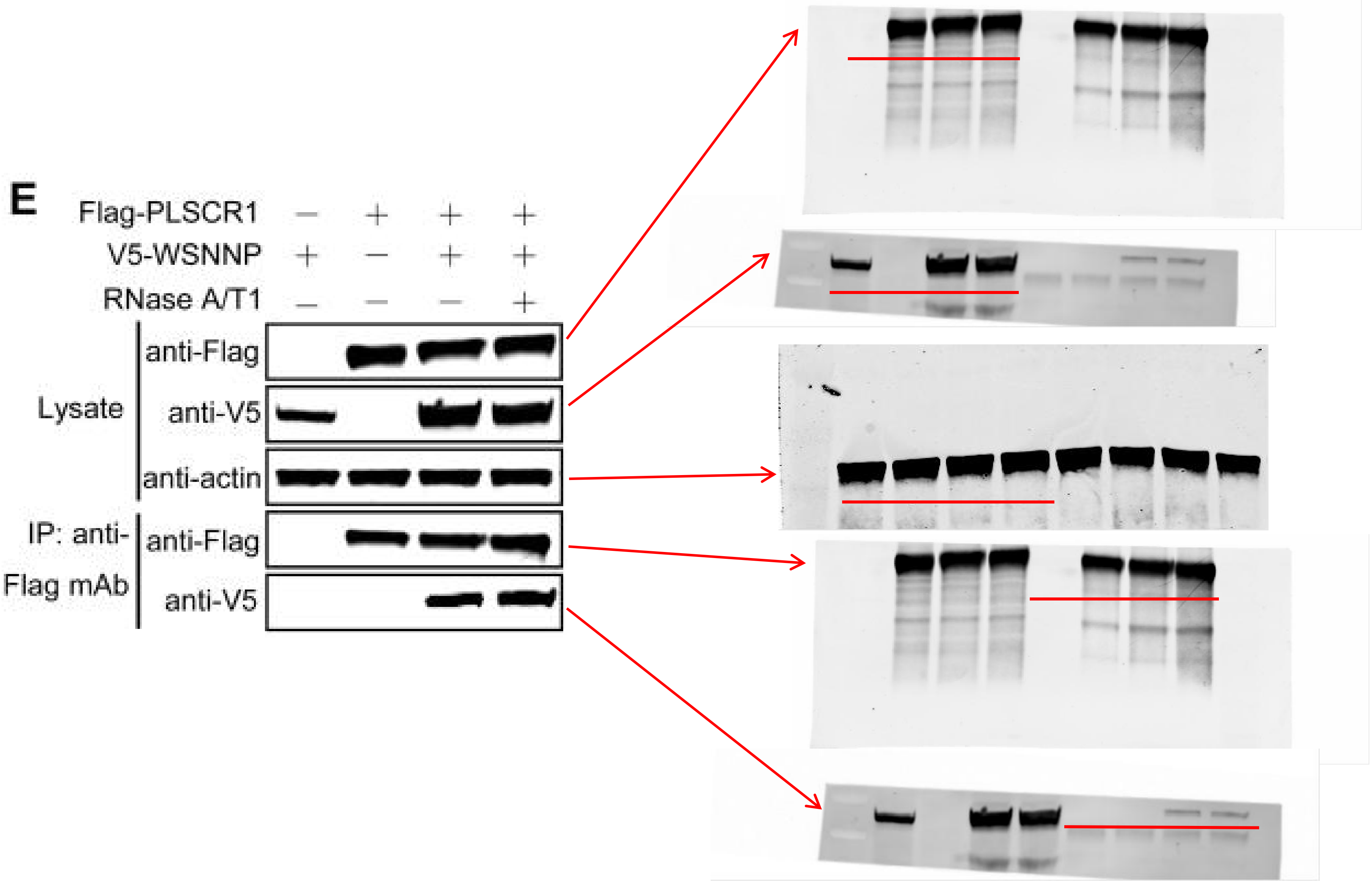
E
Flag-PLSCR1 – + + +
V5-WSNNP + – + +
RNase A/T1 – – – +
Lysate
anti-Flag
anti-V5
anti-actin
IP: anti-Flag mAb
anti-Flag
anti-V5

Fig 2F

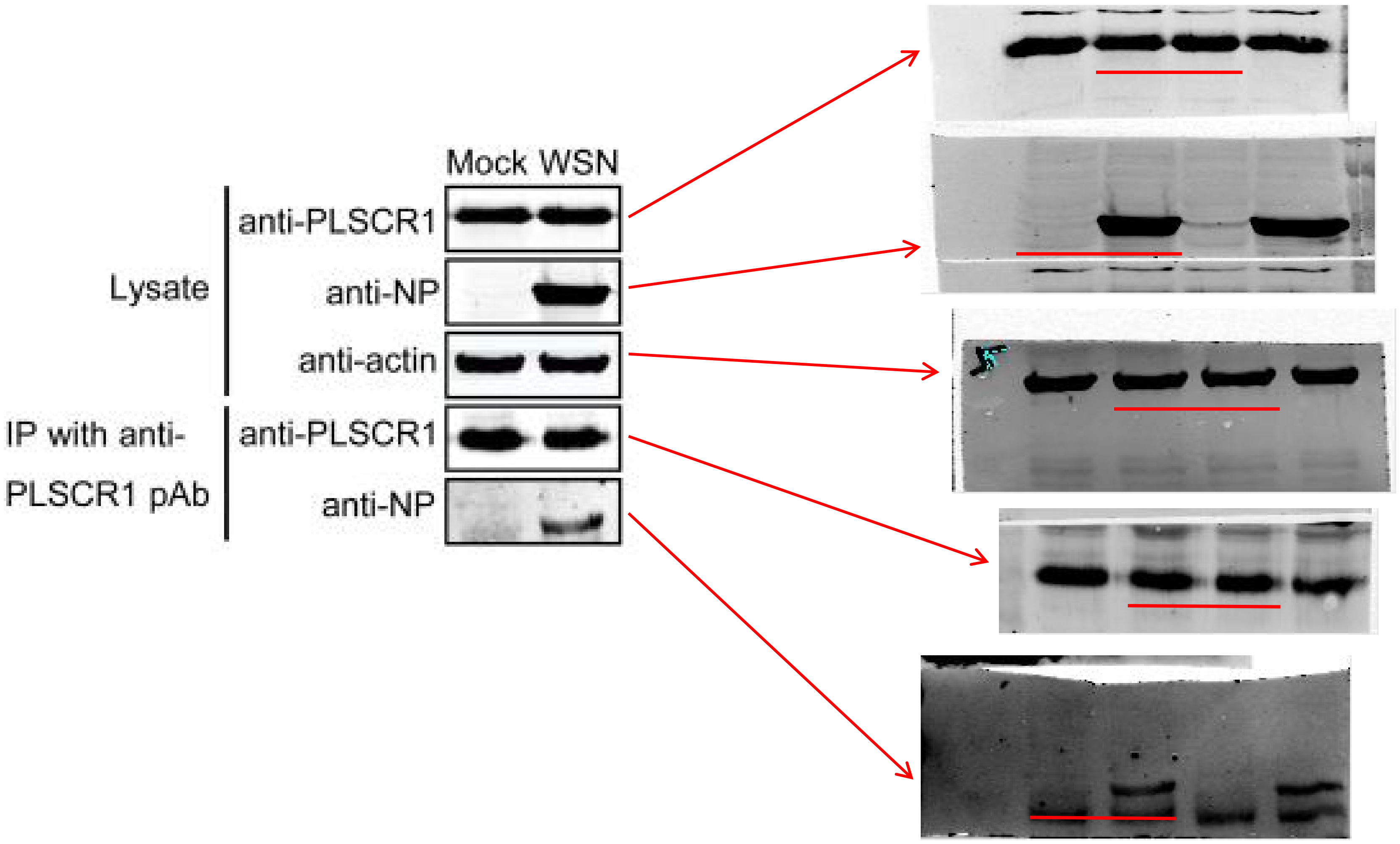
Mock WSN
Lysate
anti-PLSCR1
anti-NP
anti-actin
IP with anti-
PLSCR1 pAb
anti-PLSCR1
anti-NP

Fig 2G

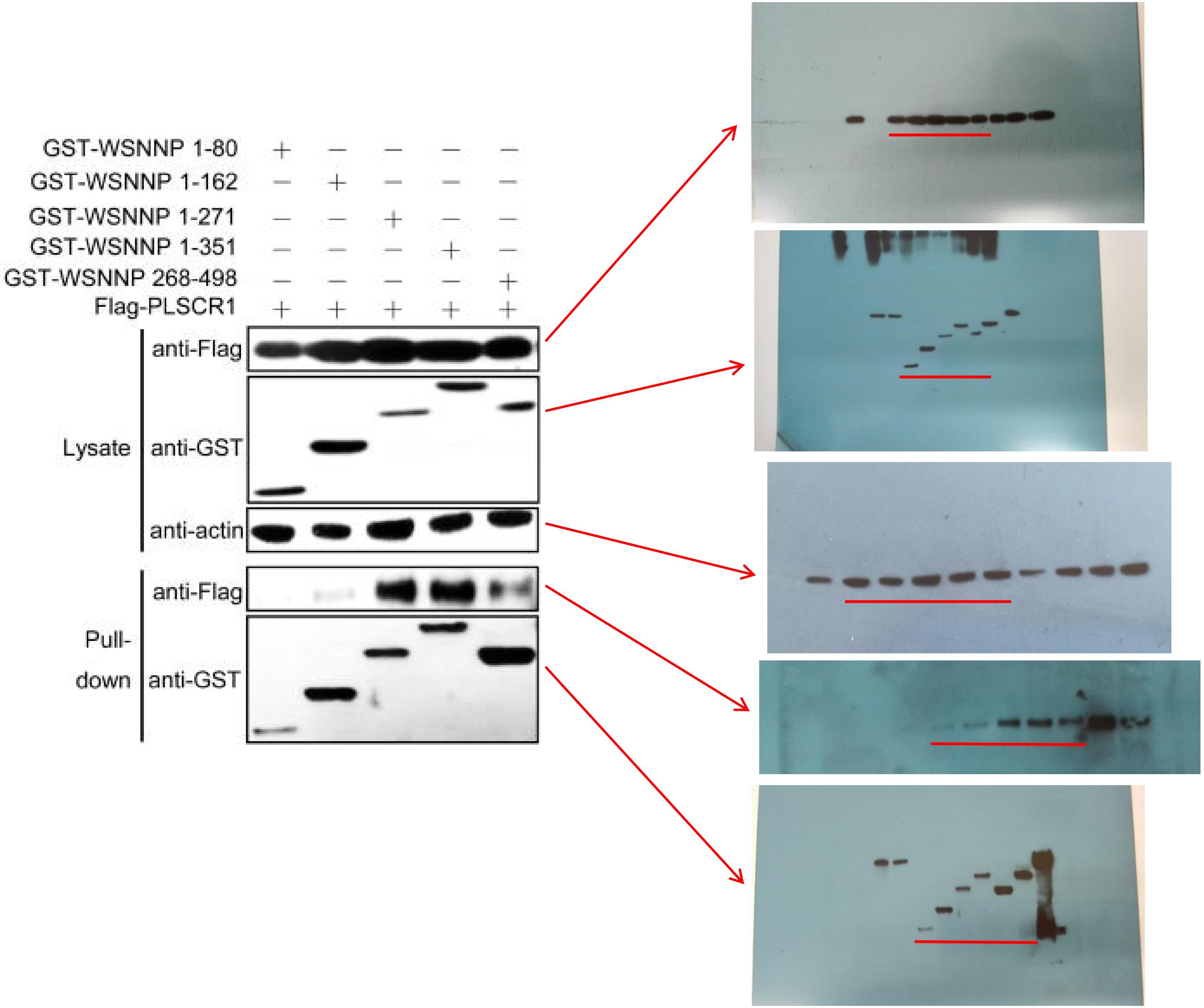
GST-WSNNP 1-80 + − − − −
GST-WSNNP 1-162 − + − − −
GST-WSNNP 1-271 − − + − −
GST-WSNNP 1-351 − − − + −
GST-WSNNP 268-498 − − − − +
Flag-PLSCR1 + + + + +
Lysate
anti-Flag
anti-GST
anti-actin
Pull-
down
anti-Flag
anti-GST

Fig 3B

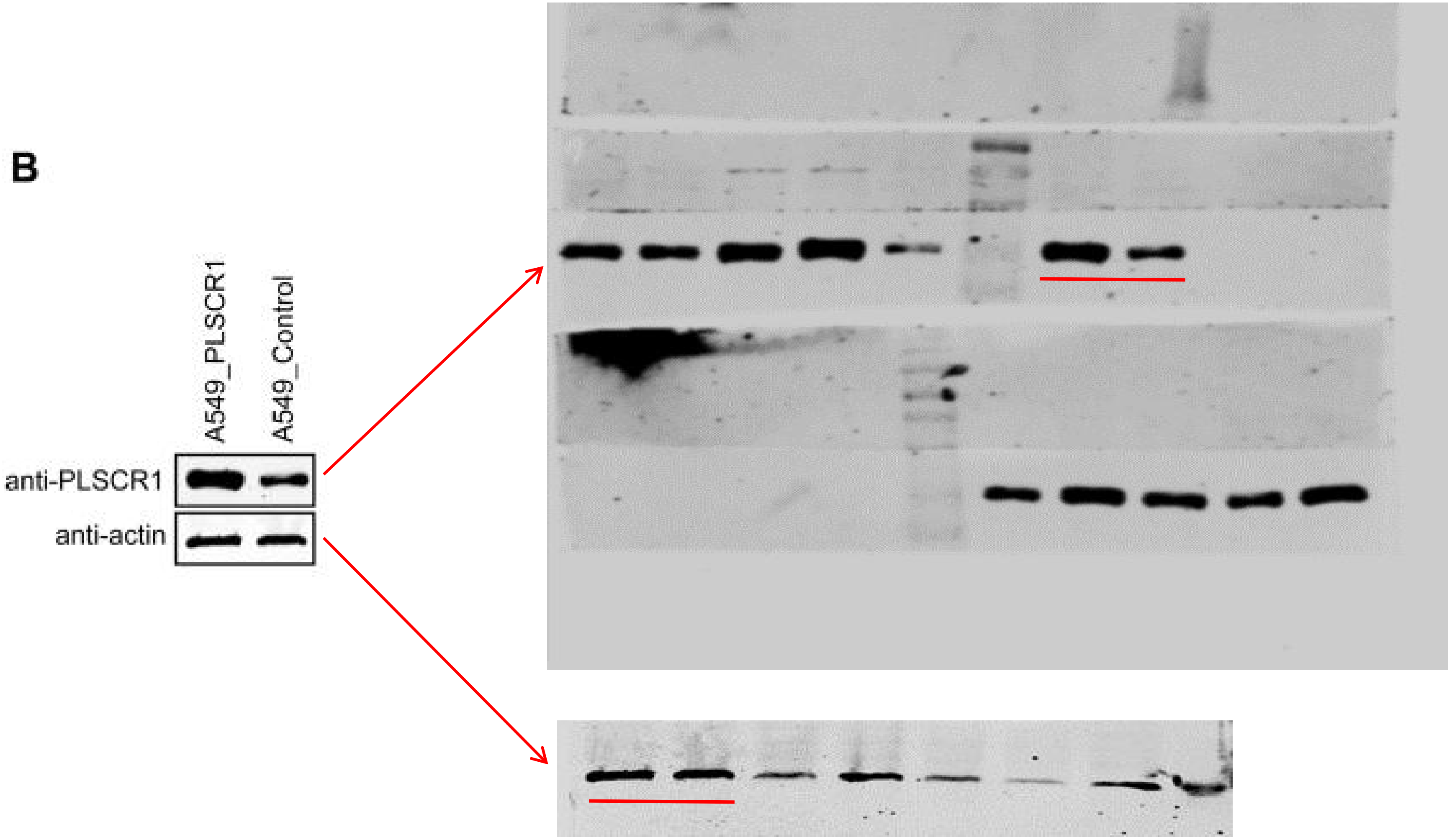

Fig 3G correction

The anti-actin image in the bottom right part of Fig 3G should be a different one. This error occurred during the assembly of the final figures. The request of correction has been submitted.

G

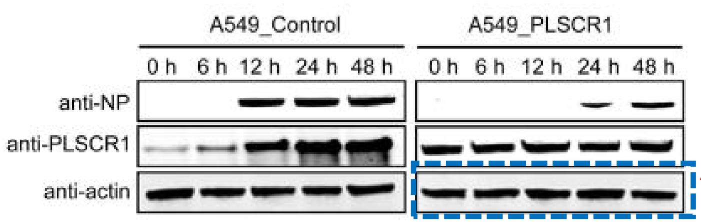

Fig 3G should be corrected into the following one.

G

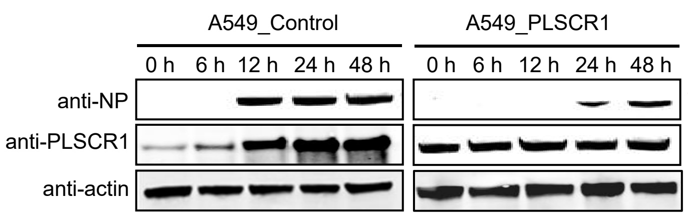

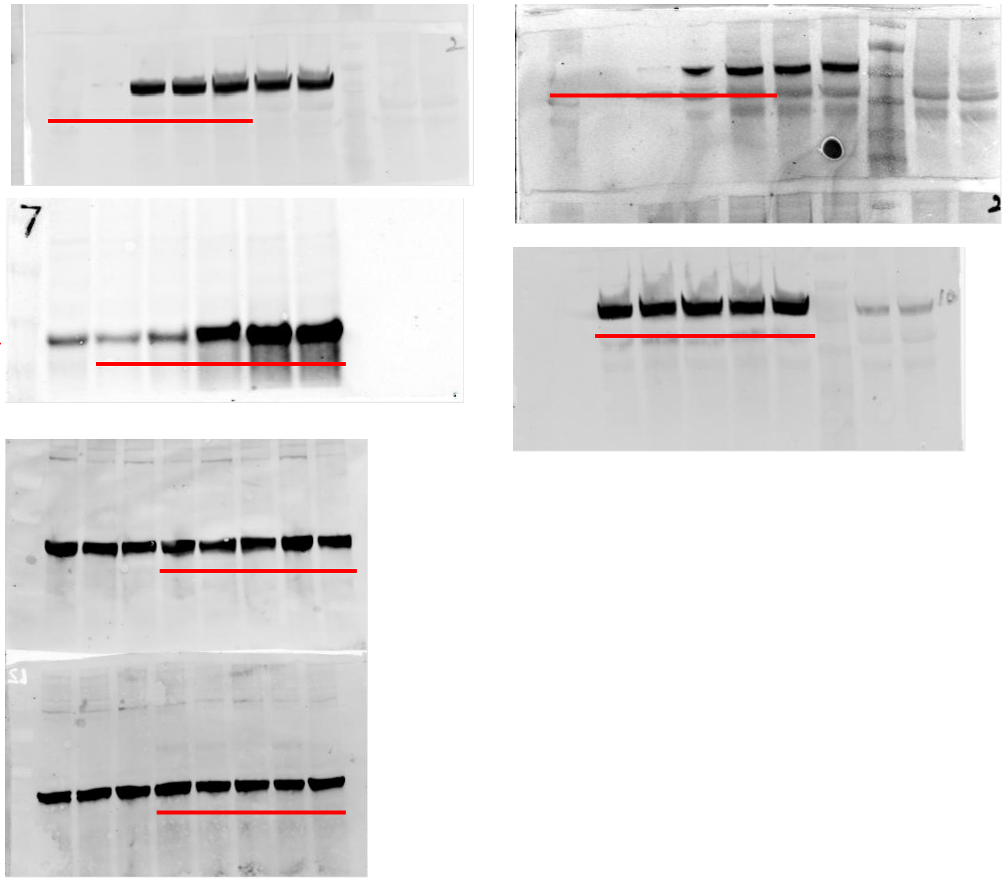

Fig 3I

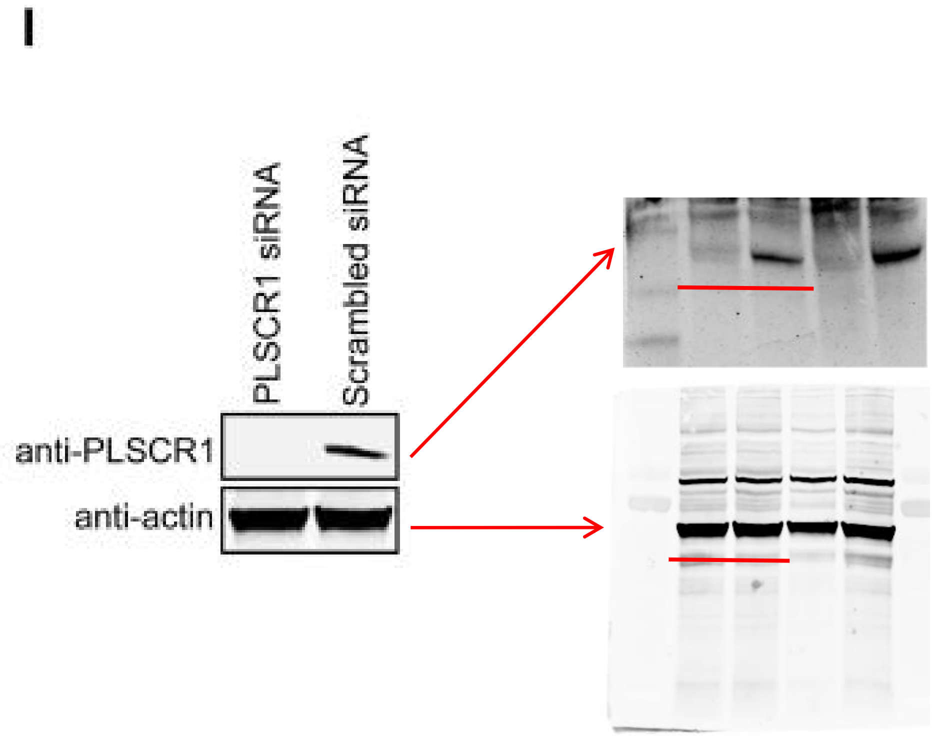

Fig 3L

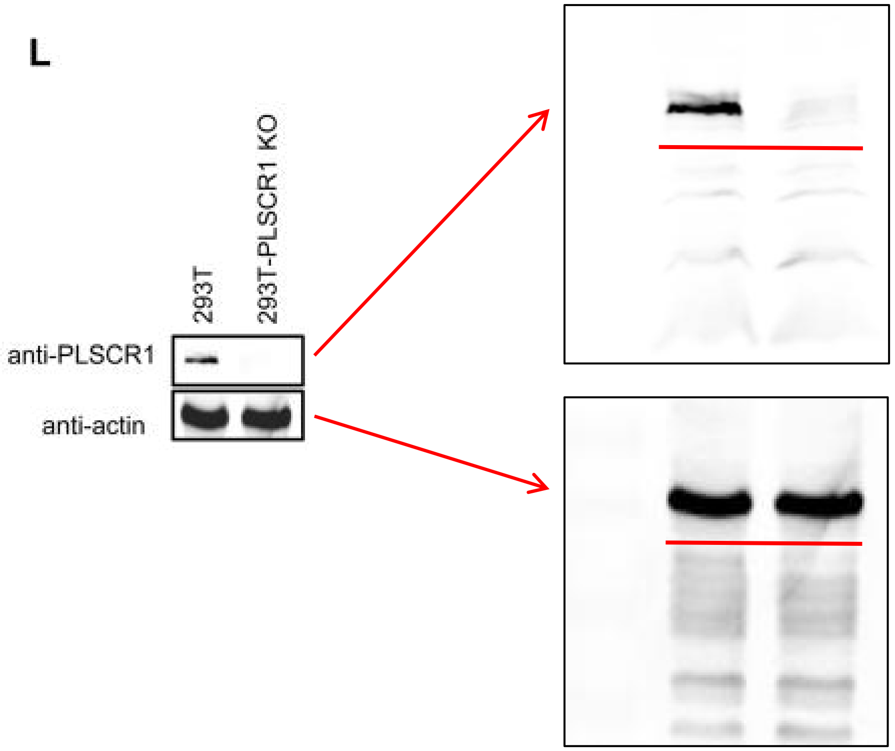

Fig 4E

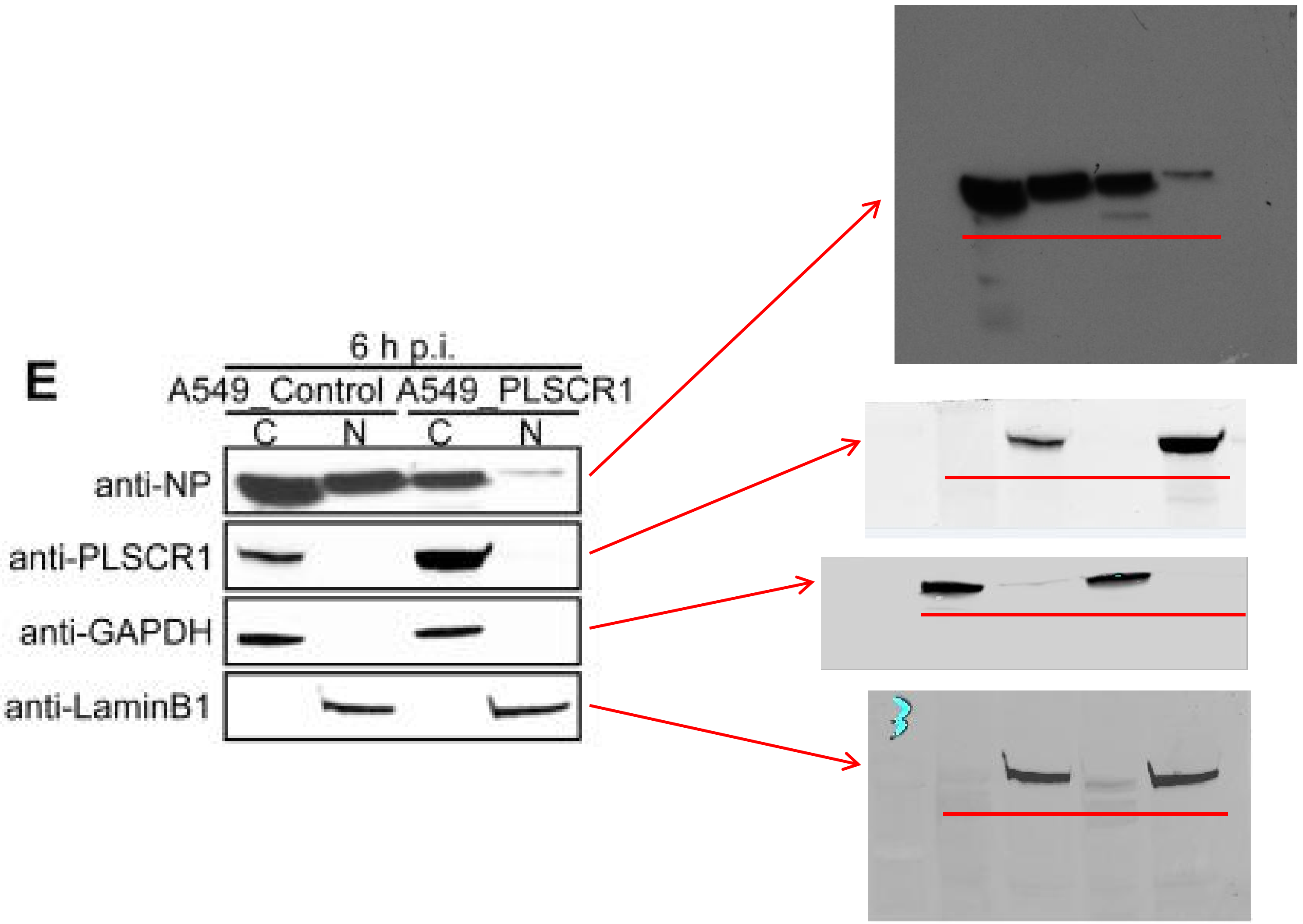
E
6 h p.i.
A549_Control
A549_PLSCR1
C
N
C
N
anti-NP
anti-PLSCR1
anti-GAPDH
anti-LaminB1

Fig 4F

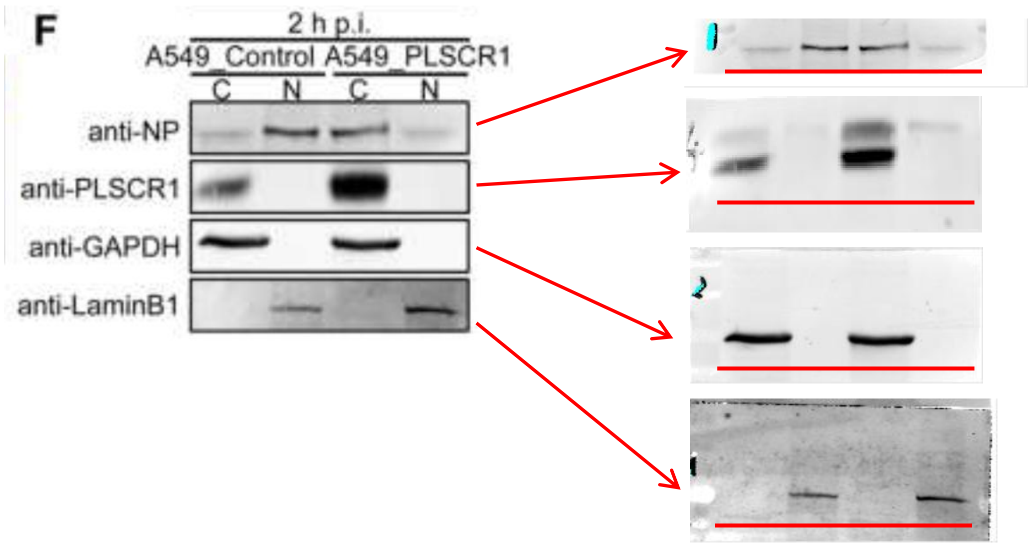
F
2 h p.i.
A549_Control A549_PLSCR1
C N C N
anti-NP
anti-PLSCR1
anti-GAPDH
anti-LaminB1

Fig 5A

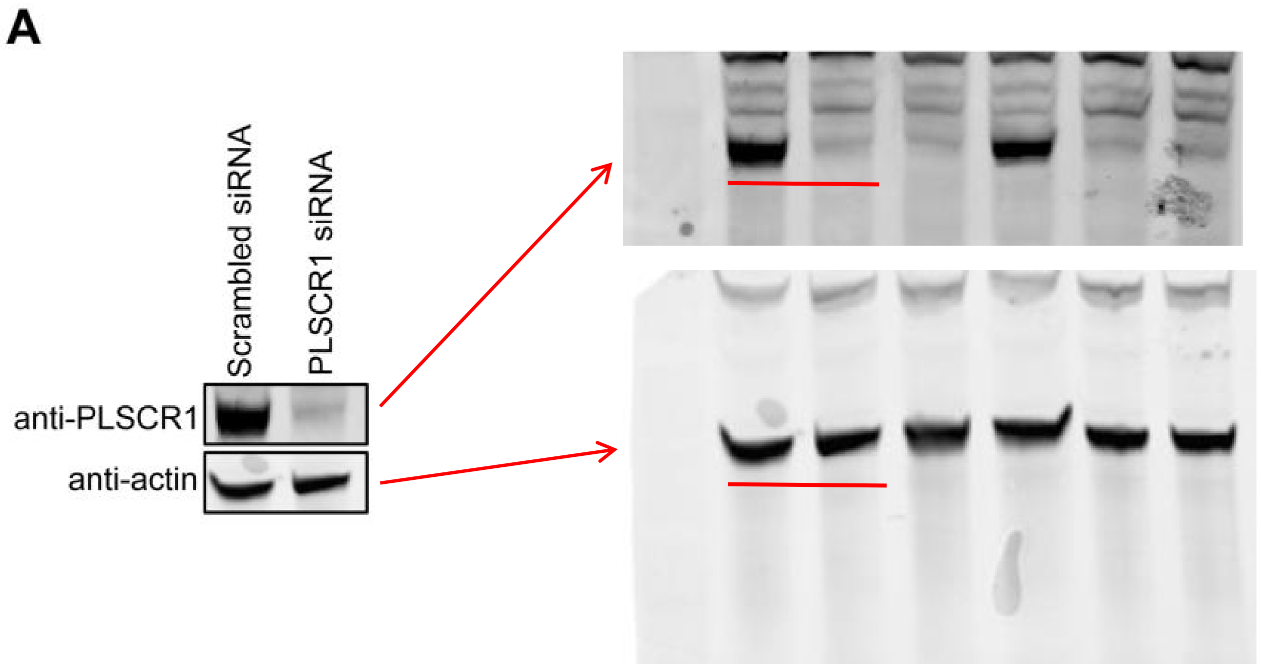

Fig 6A

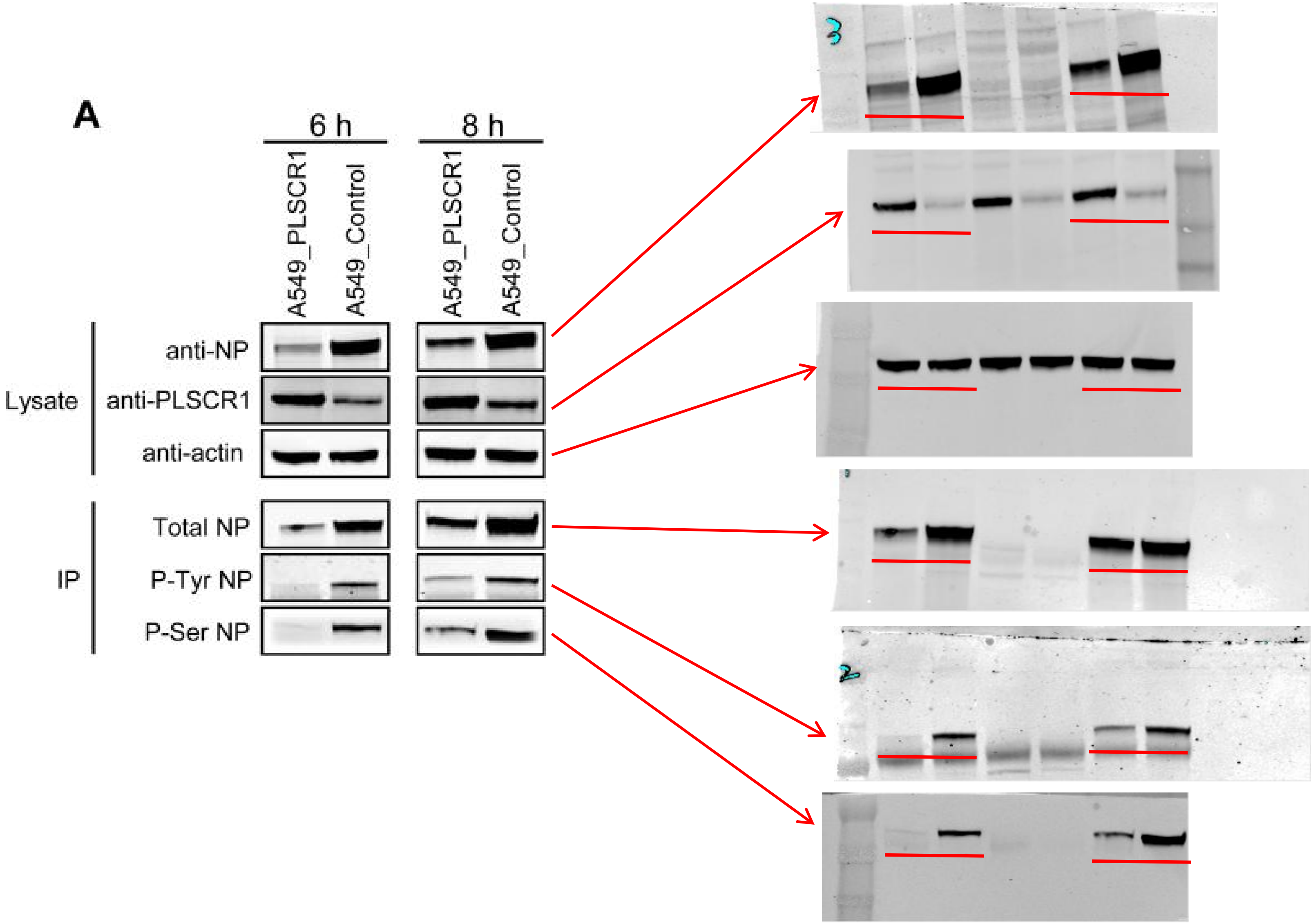
A
6 h
8 h
A549_PLSCR1
A549_Control
A549_PLSCR1
A549_Control
Lysate
anti-NP
anti-PLSCR1
anti-actin
IP
Total NP
P-Tyr NP
P-Ser NP

Fig 6C

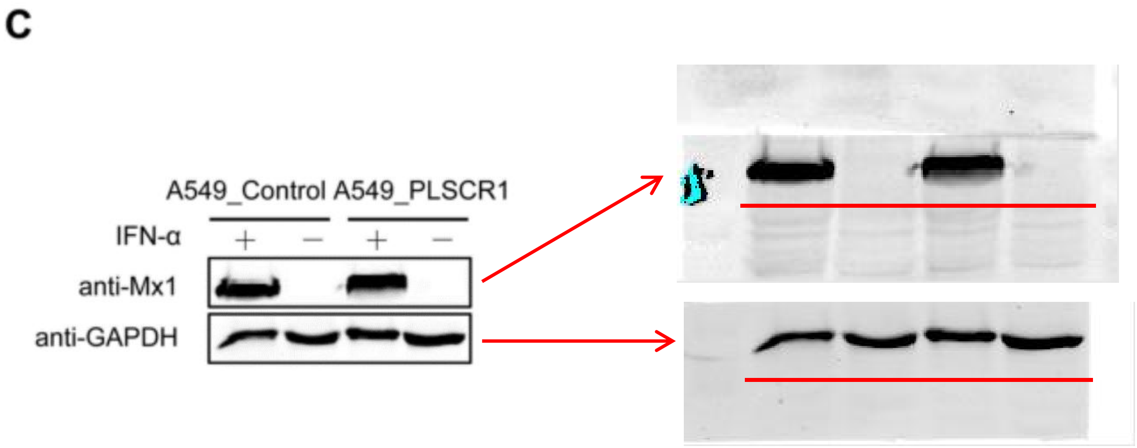

Fig 6D

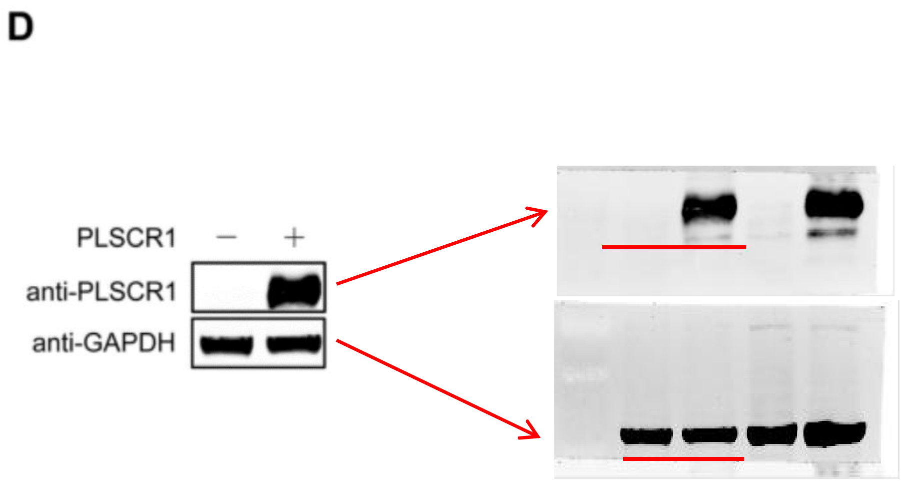

Fig 7A

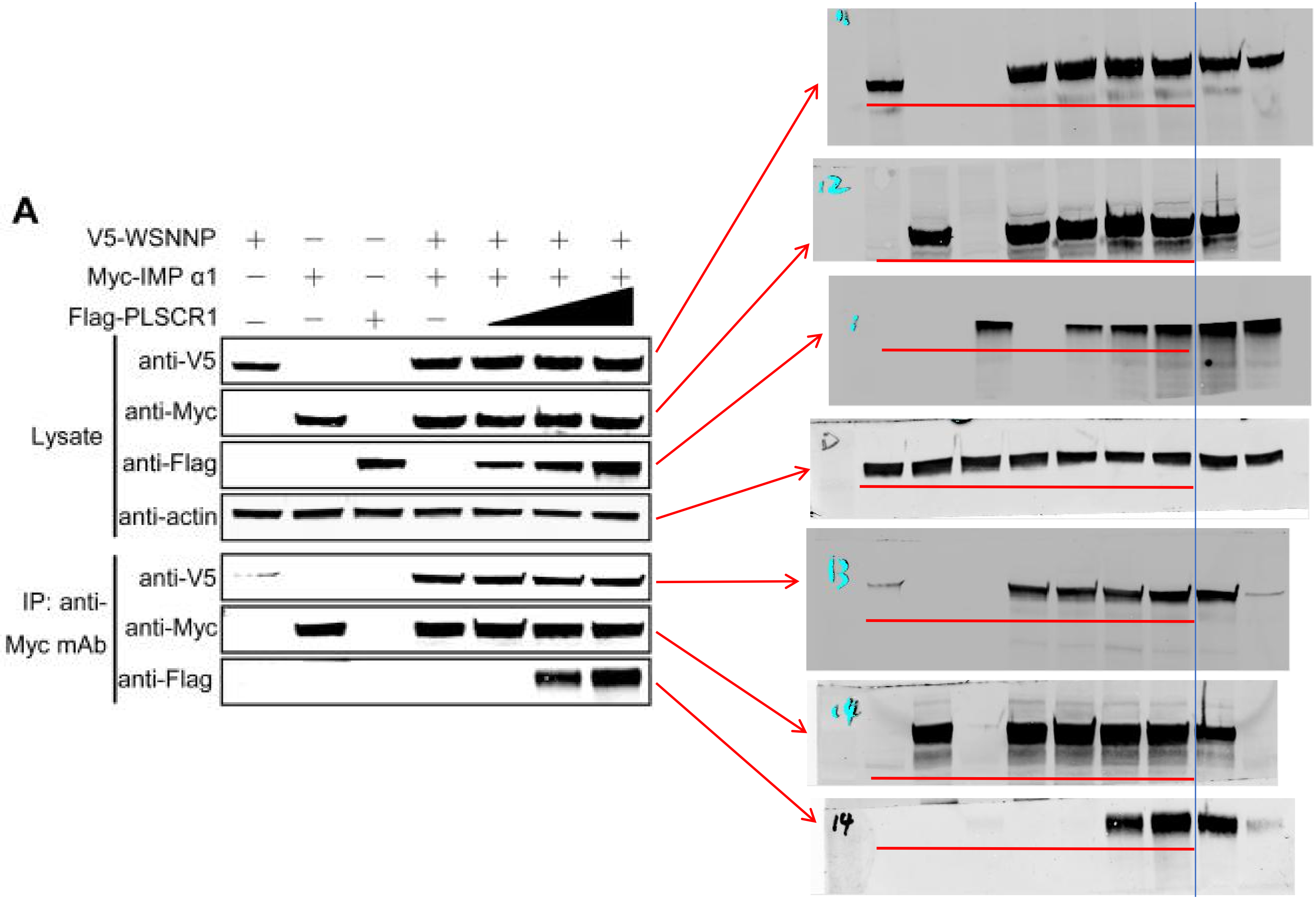
A
V5-WSNNP
Myc-IMP α1
Flag-PLSCR1
anti-V5
anti-Myc
anti-Flag
anti-actin
Lysate
IP: anti-
Myc mAb
anti-V5
anti-Myc
anti-Flag
12
1
13
14

Fig 7B

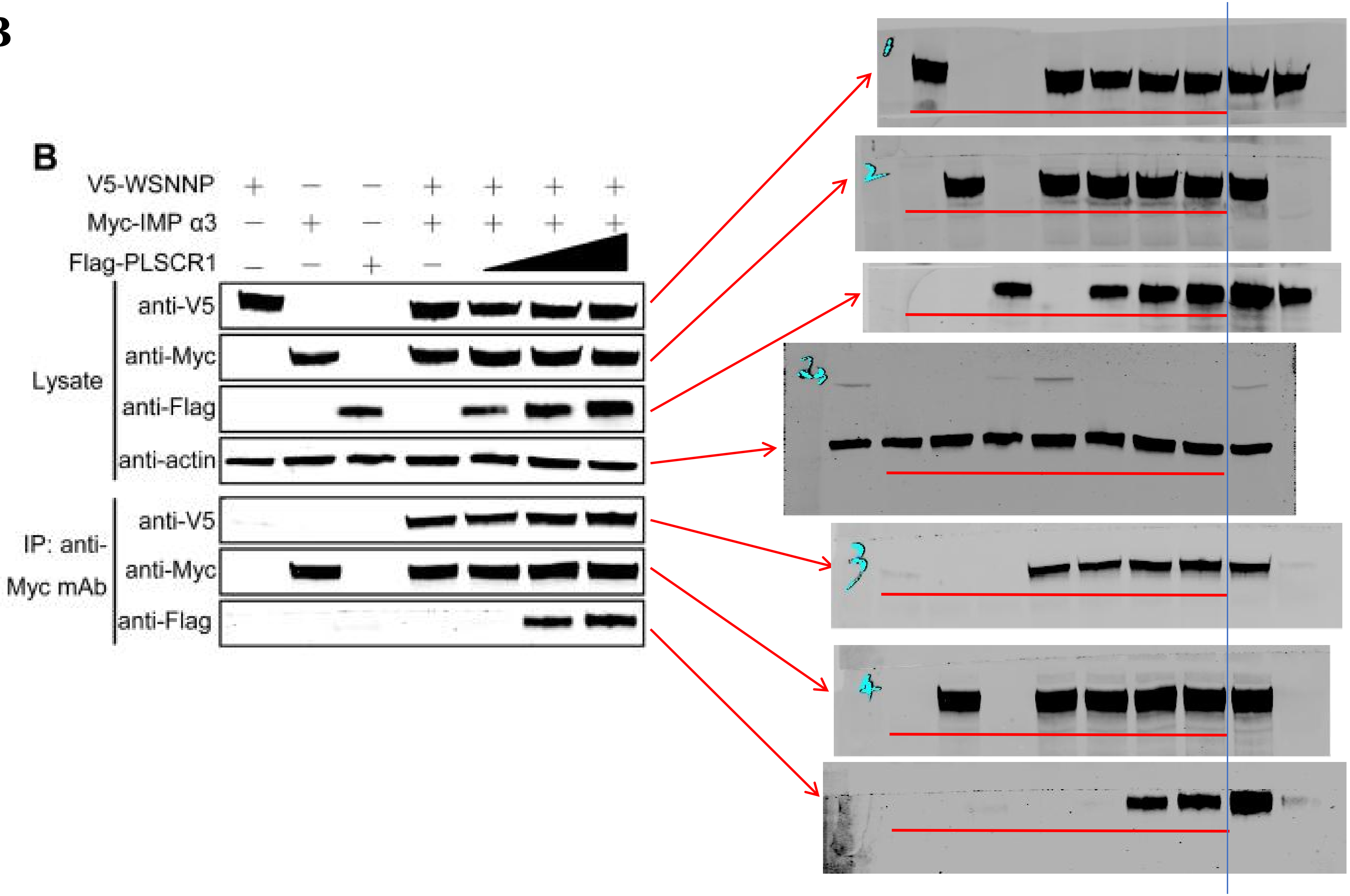
B
V5-WSNNP + − − + + + +
Myc-IMP α3 − + − + + + +
Flag-PLSCR1 − − + −
Lysate
anti-V5
anti-Myc
anti-Flag
anti-actin
IP: anti-Myc mAb
anti-V5
anti-Myc
anti-Flag

Fig 7C correction

This band is redundant and should be removed.

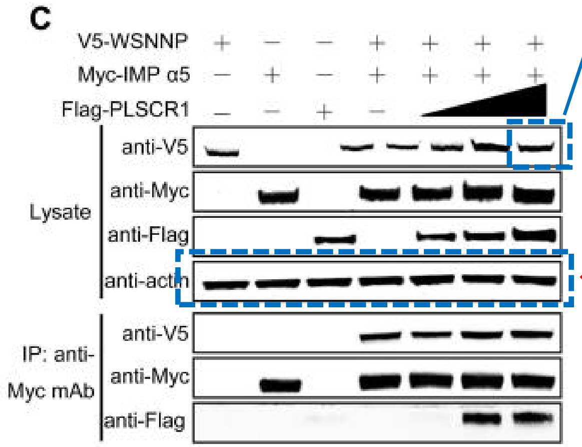

The lysate anti-actin image in Fig 7C should be a different one. This error occurred during the assembly of the final figures. The request of correction has been submitted.

Fig 7C should be corrected into the following one.

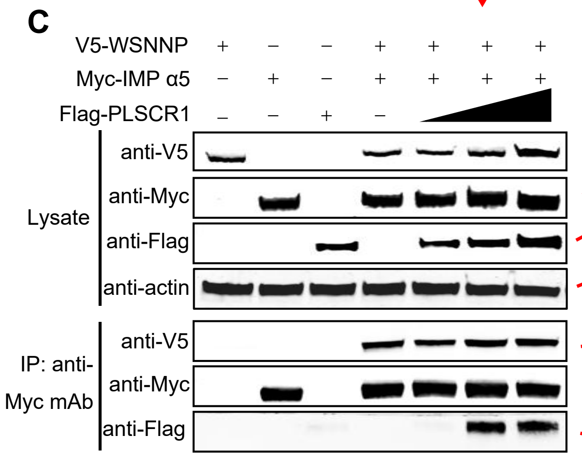

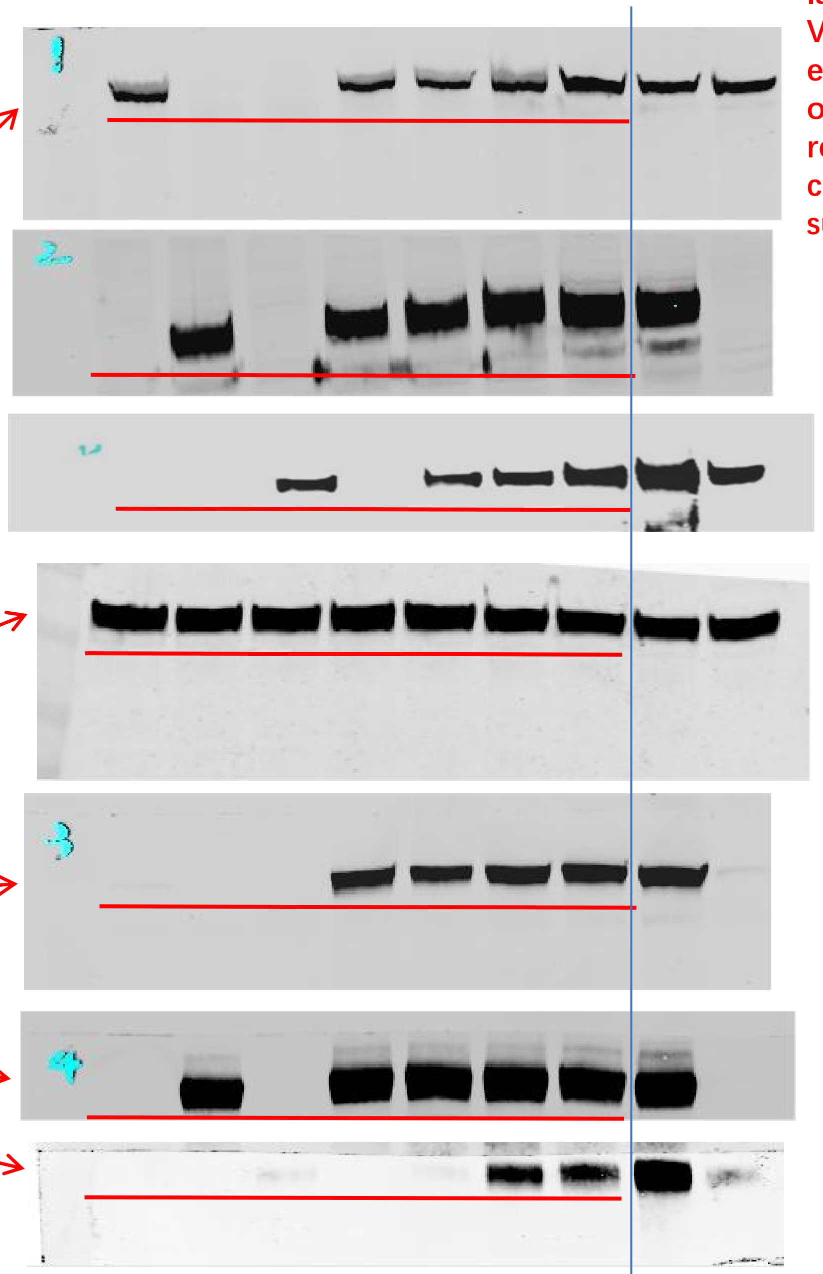

Fig 7C should contain seven instead of eight lanes in the lysate anti-V5 image, and the eighth lane in this piece of image should be removed. The request of correction has been submitted.

Fig 7D

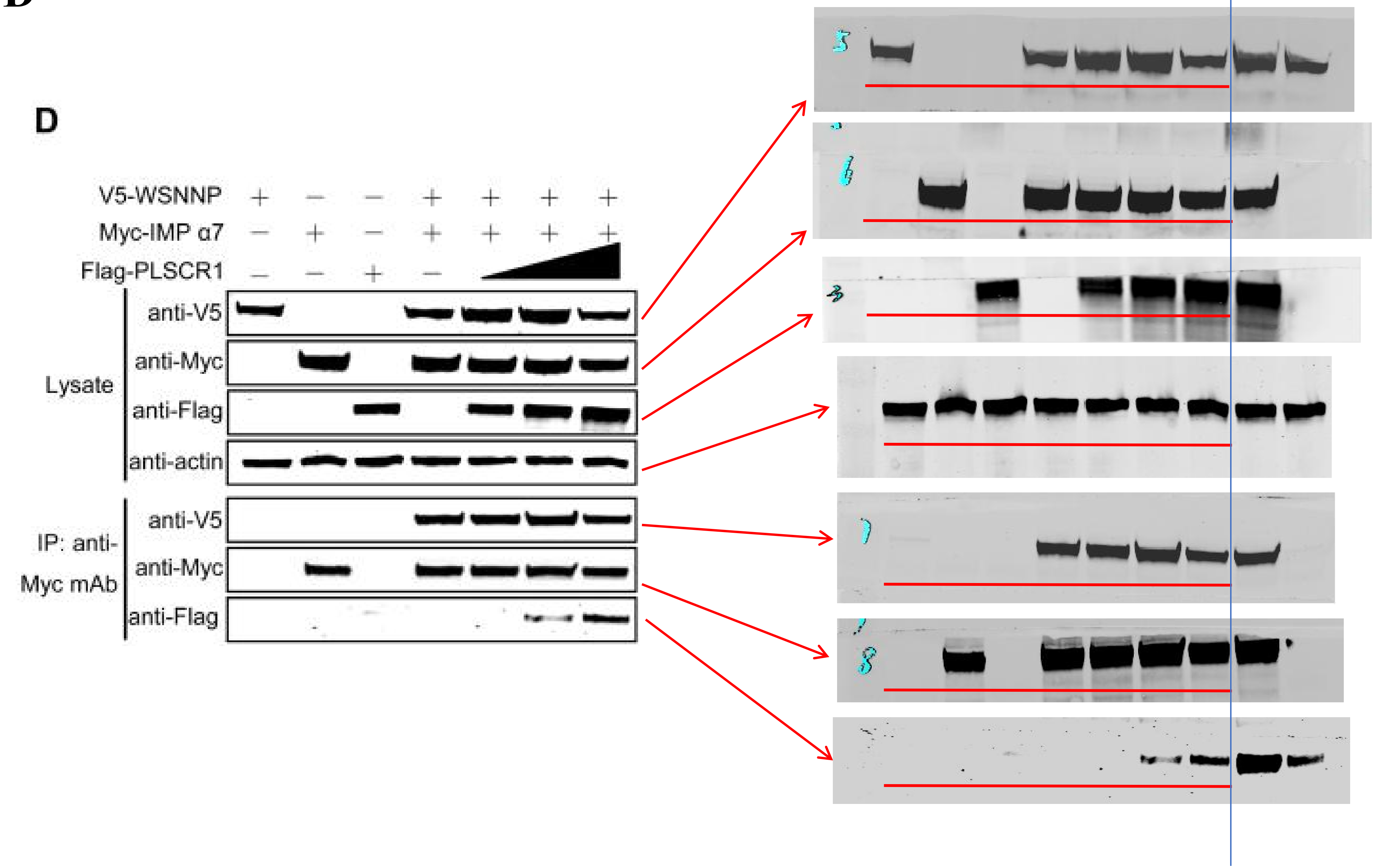
D
V5-WSNNP + − − + + + +
Myc-IMP α7 − + − + + + +
Flag-PLSCR1 − − + −
Lysate
anti-V5
anti-Myc
anti-Flag
anti-actin
IP: anti-
Myc mAb
anti-V5
anti-Myc
anti-Flag

Fig 7E

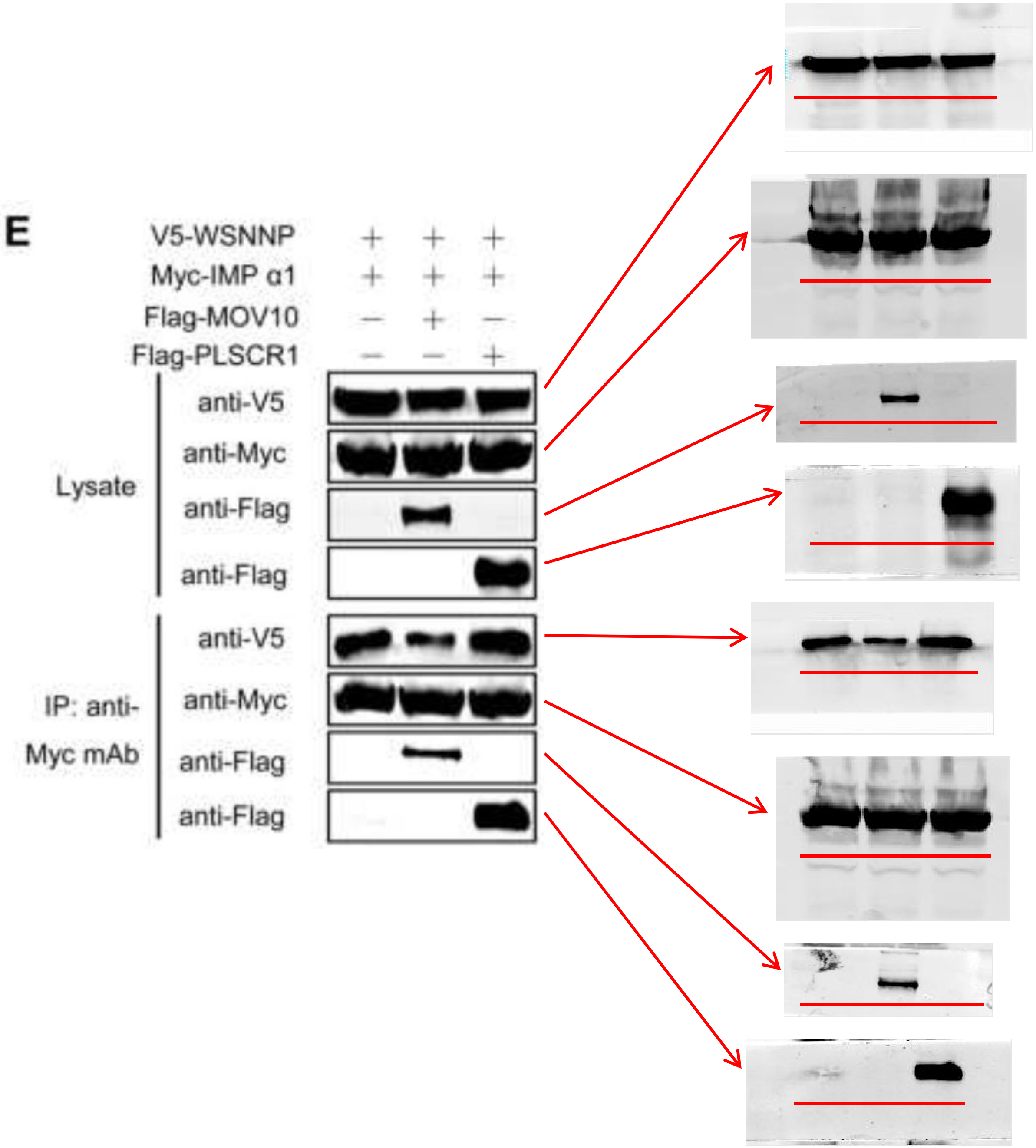
E
V5-WSNNP + + +
Myc-IMP α1 + + +
Flag-MOV10 − + −
Flag-PLSCR1 − − +
Lysate
anti-V5
anti-Myc
anti-Flag
anti-Flag
IP: anti-Myc mAb
anti-V5
anti-Myc
anti-Flag
anti-Flag

Fig 7F

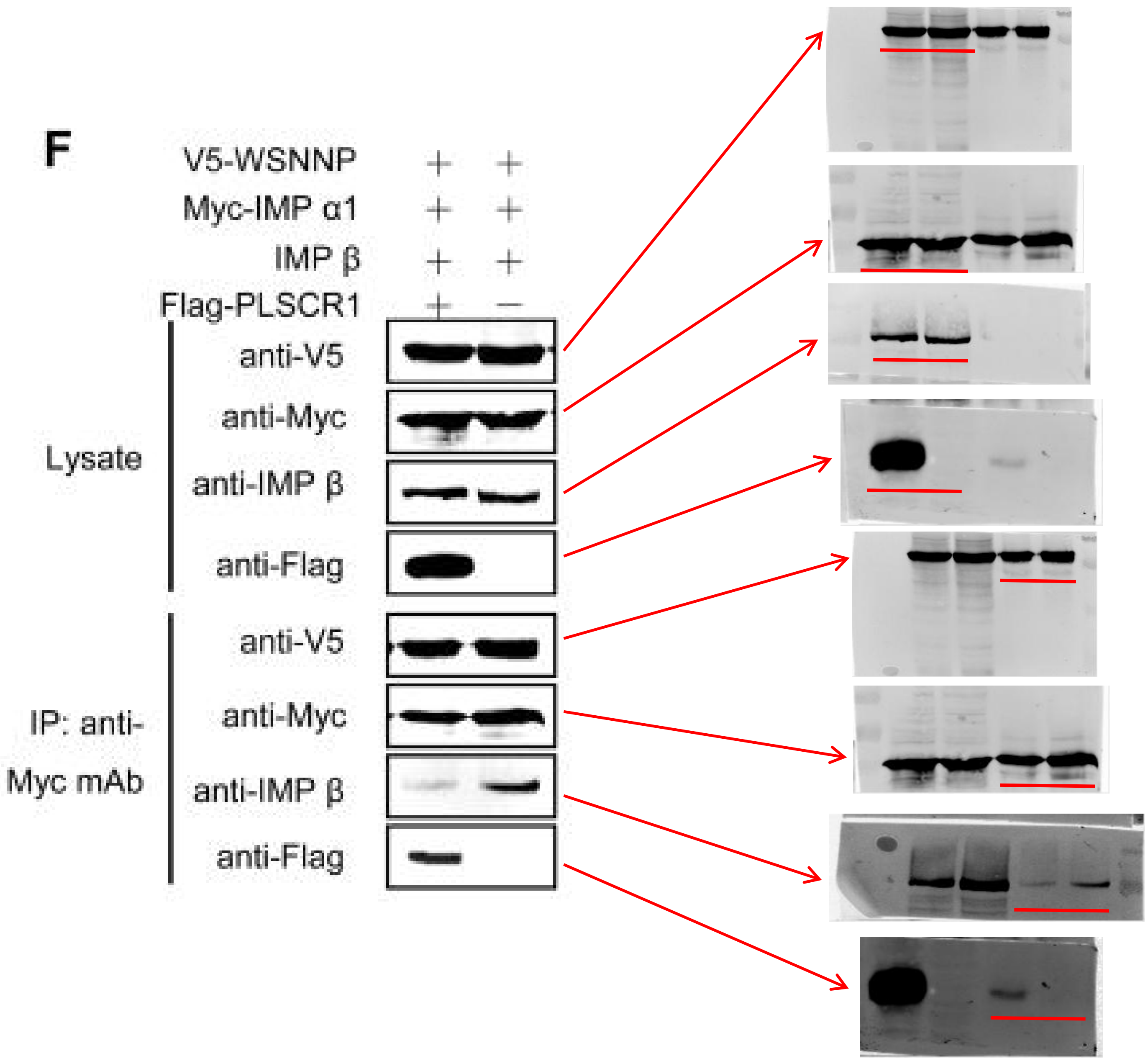
F
V5-WSNNP + +
Myc-IMP α1 + +
IMP β + +
Flag-PLSCR1 + −
Lysate
anti-V5
anti-Myc
anti-IMP β
anti-Flag
IP: anti-Myc mAb
anti-V5
anti-Myc
anti-IMP β
anti-Flag
