# Supplementary figures and images for: Correction: Phospholipid scramblase 1 interacts with influenza A virus NP, impairing its nuclear import and thereby suppressing virus replication
Source: PLoS Pathog. 2024 Feb 23;20(2):e1012035. doi: 10.1371/journal.ppat.1012035 (PMC10889636; doi:10.1371/journal.ppat.1012035)

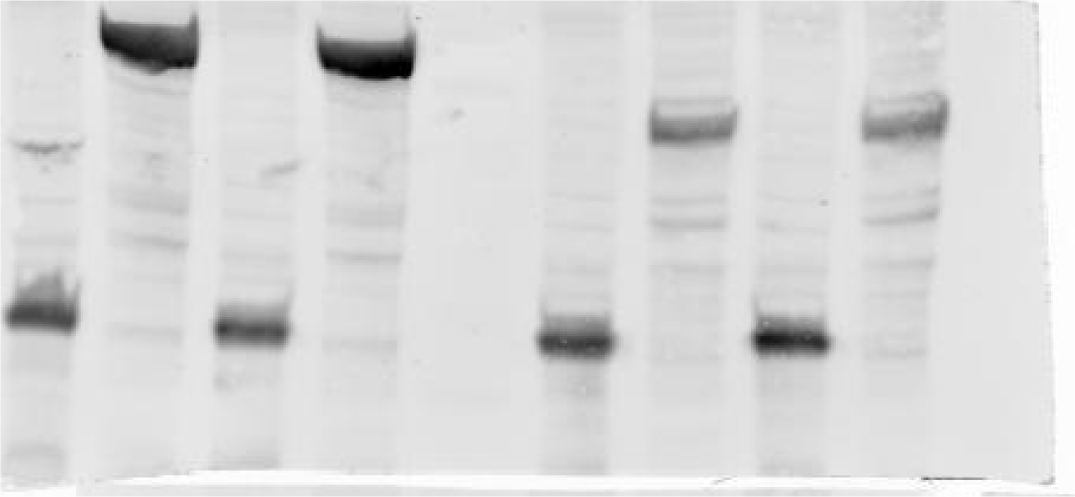

Supplement: S1 File — (ZIP) [file ppat.1012035.s001.zip › S1 File. Original western blot images/Fig 2/Fig 2C Lysate GST.tif]

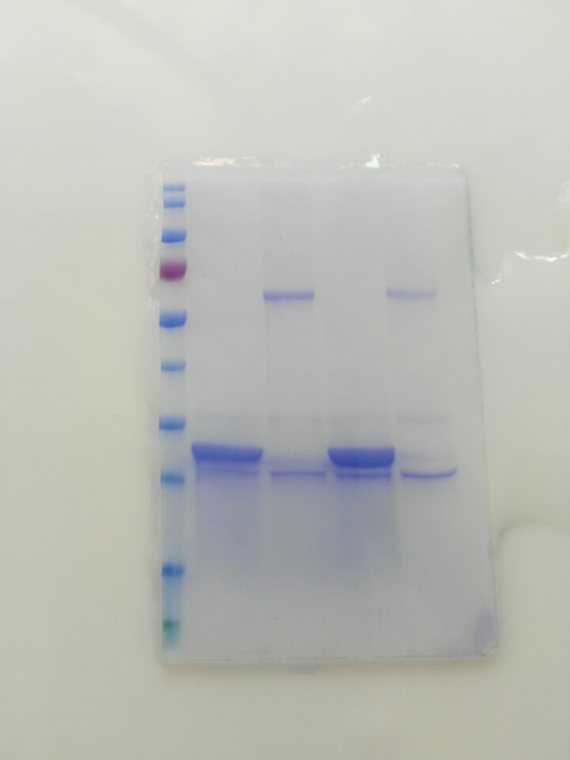

Supplement: S1 File — (ZIP) [file ppat.1012035.s001.zip › S1 File. Original western blot images/Fig 2/Fig 2C Pull-down GST.tif]

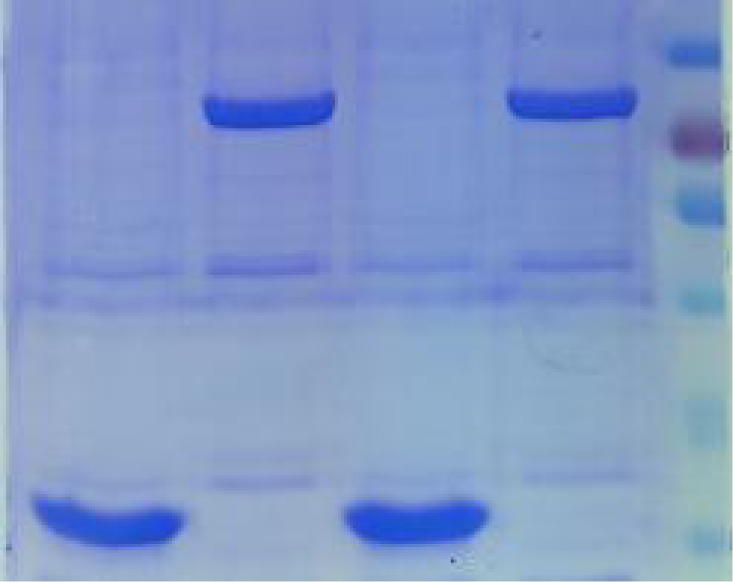

Supplement: S1 File — (ZIP) [file ppat.1012035.s001.zip › S1 File. Original western blot images/Fig 2/Fig 2D Pull-down GST-WSNNP.tif]

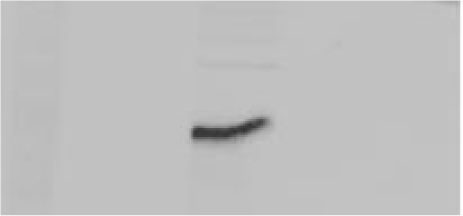

Supplement: S1 File — (ZIP) [file ppat.1012035.s001.zip › S1 File. Original western blot images/Fig 2/Fig 2D Pull-down PLSCR1.tif]

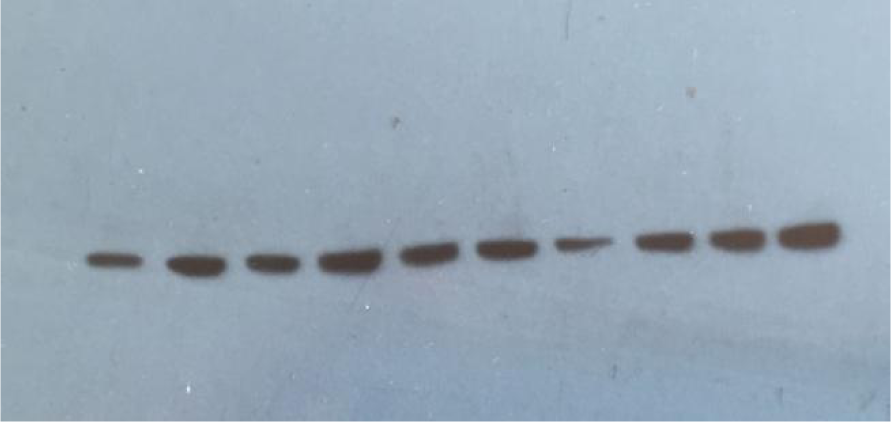

Supplement: S1 File — (ZIP) [file ppat.1012035.s001.zip › S1 File. Original western blot images/Fig 2/Fig 2G Lysate actin.tif]

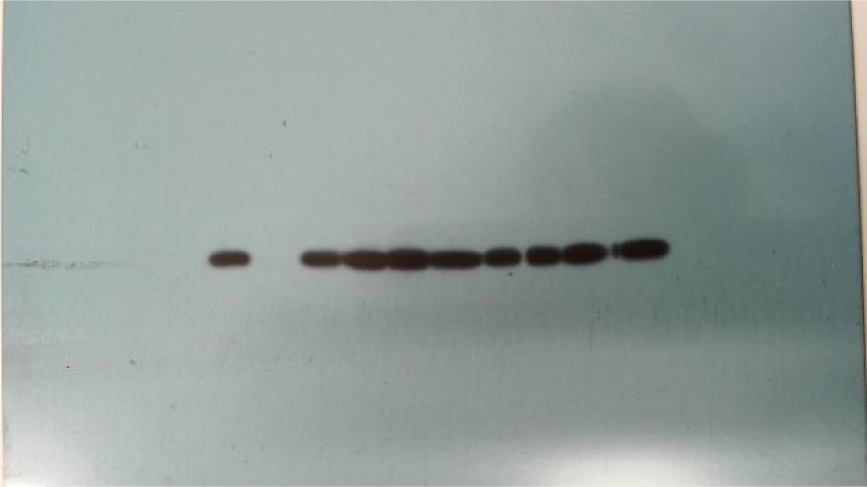

Supplement: S1 File — (ZIP) [file ppat.1012035.s001.zip › S1 File. Original western blot images/Fig 2/Fig 2G Lysate Flag-PLSCR1.tif]

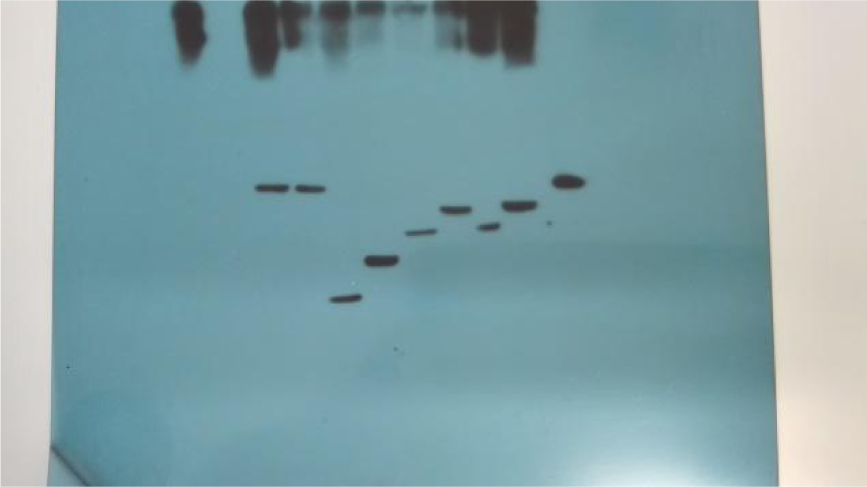

Supplement: S1 File — (ZIP) [file ppat.1012035.s001.zip › S1 File. Original western blot images/Fig 2/Fig 2G Lysate GST.tif]

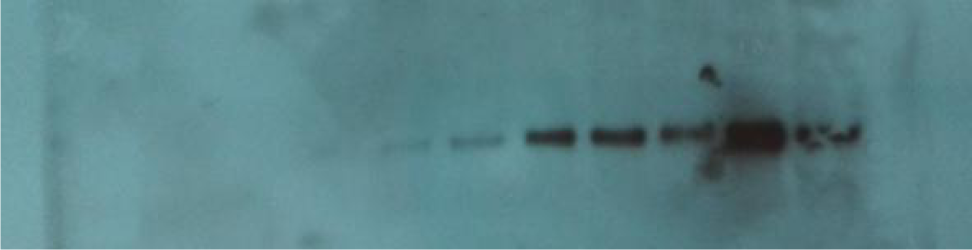

Supplement: S1 File — (ZIP) [file ppat.1012035.s001.zip › S1 File. Original western blot images/Fig 2/Fig 2G Pull-down Flag-PLSCR1.tif]

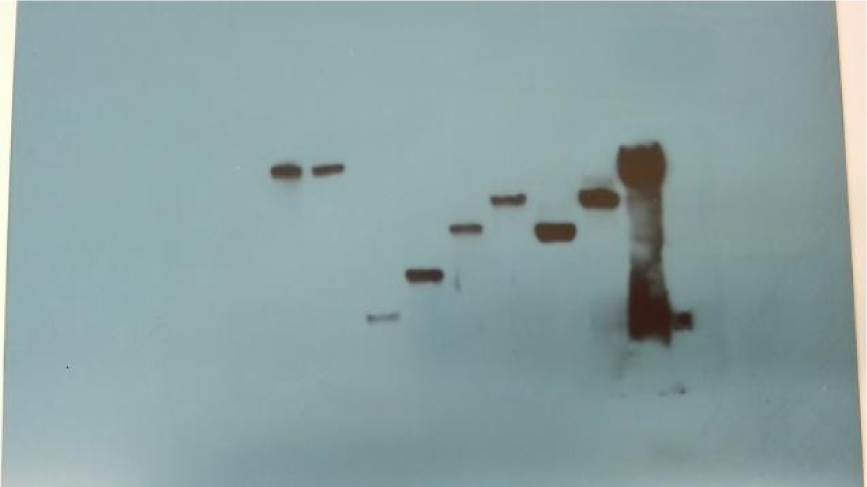

Supplement: S1 File — (ZIP) [file ppat.1012035.s001.zip › S1 File. Original western blot images/Fig 2/Fig 2G Pull-down GST.tif]

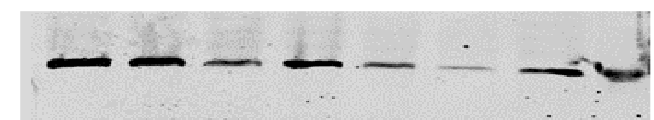

Supplement: S1 File — (ZIP) [file ppat.1012035.s001.zip › S1 File. Original western blot images/Fig 3/Fig 3B actin.tif]

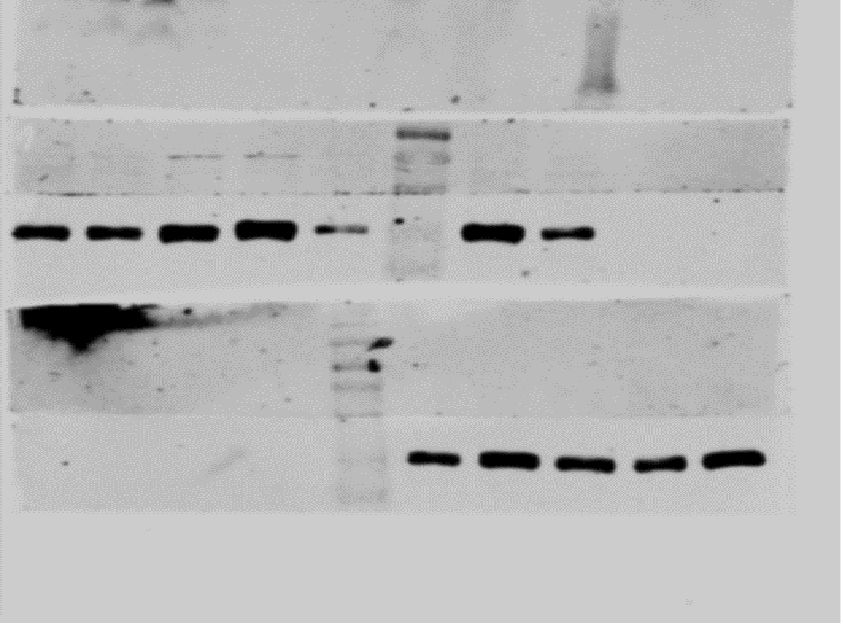

Supplement: S1 File — (ZIP) [file ppat.1012035.s001.zip › S1 File. Original western blot images/Fig 3/Fig 3B PLSCR1.tif]

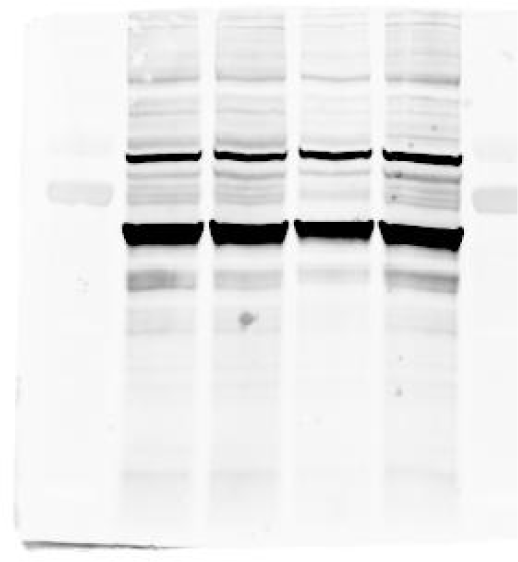

Supplement: S1 File — (ZIP) [file ppat.1012035.s001.zip › S1 File. Original western blot images/Fig 3/Fig 3I actin.tif]

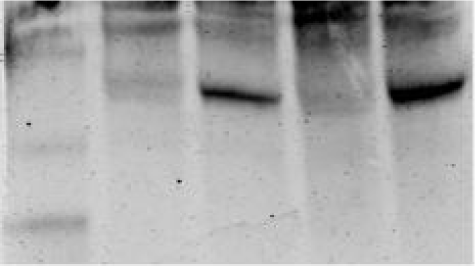

Supplement: S1 File — (ZIP) [file ppat.1012035.s001.zip › S1 File. Original western blot images/Fig 3/Fig 3I PLSCR1.tif]

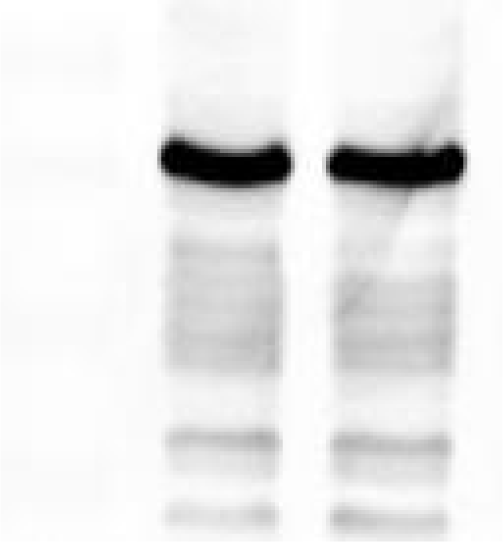

Supplement: S1 File — (ZIP) [file ppat.1012035.s001.zip › S1 File. Original western blot images/Fig 3/Fig 3L actin.tif]

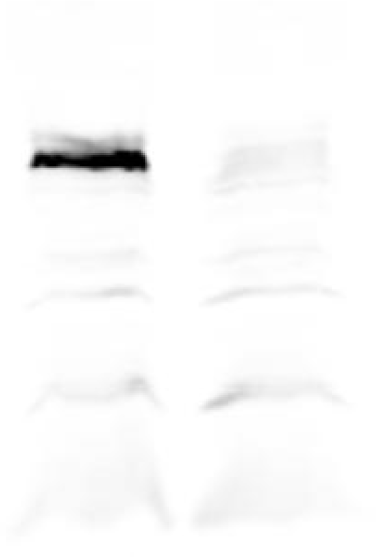

Supplement: S1 File — (ZIP) [file ppat.1012035.s001.zip › S1 File. Original western blot images/Fig 3/Fig 3L PLSCR1.tif]

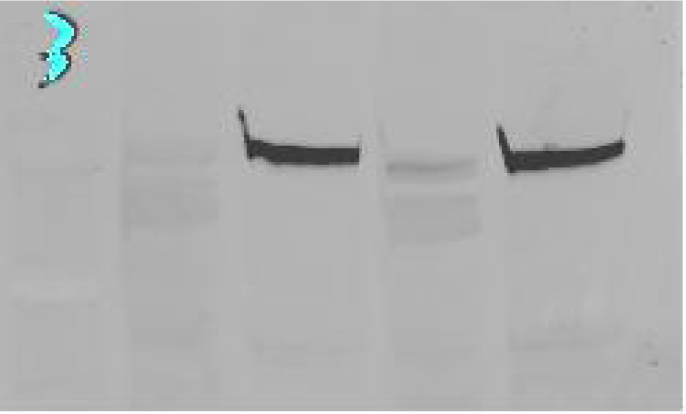

Supplement: S1 File — (ZIP) [file ppat.1012035.s001.zip › S1 File. Original western blot images/Fig 4/Fig 4E LaminB1.tif]

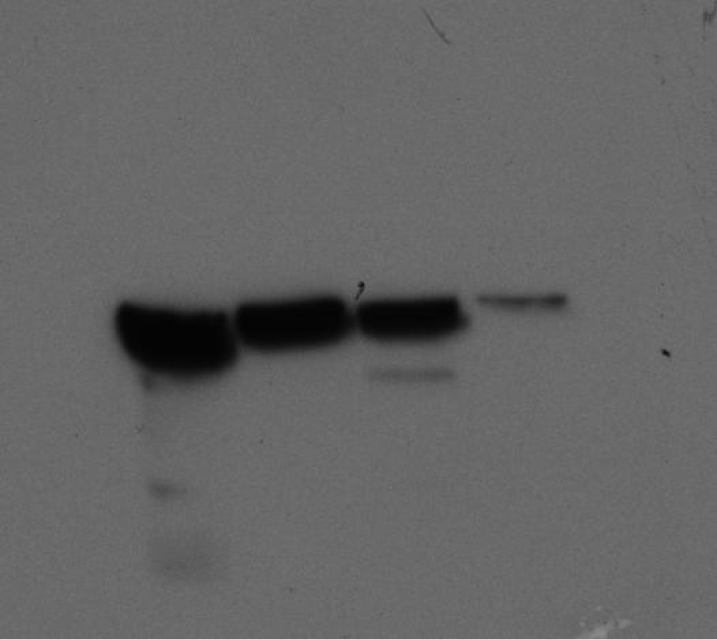

Supplement: S1 File — (ZIP) [file ppat.1012035.s001.zip › S1 File. Original western blot images/Fig 4/Fig 4E NP.tif]

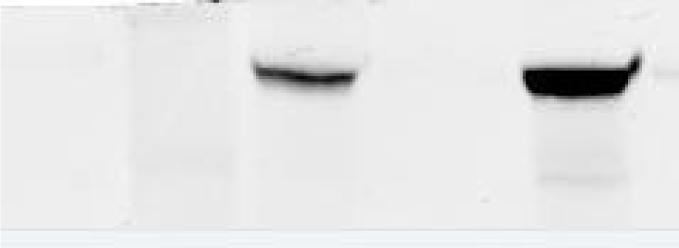

Supplement: S1 File — (ZIP) [file ppat.1012035.s001.zip › S1 File. Original western blot images/Fig 4/Fig 4E PLSCR1.tif]

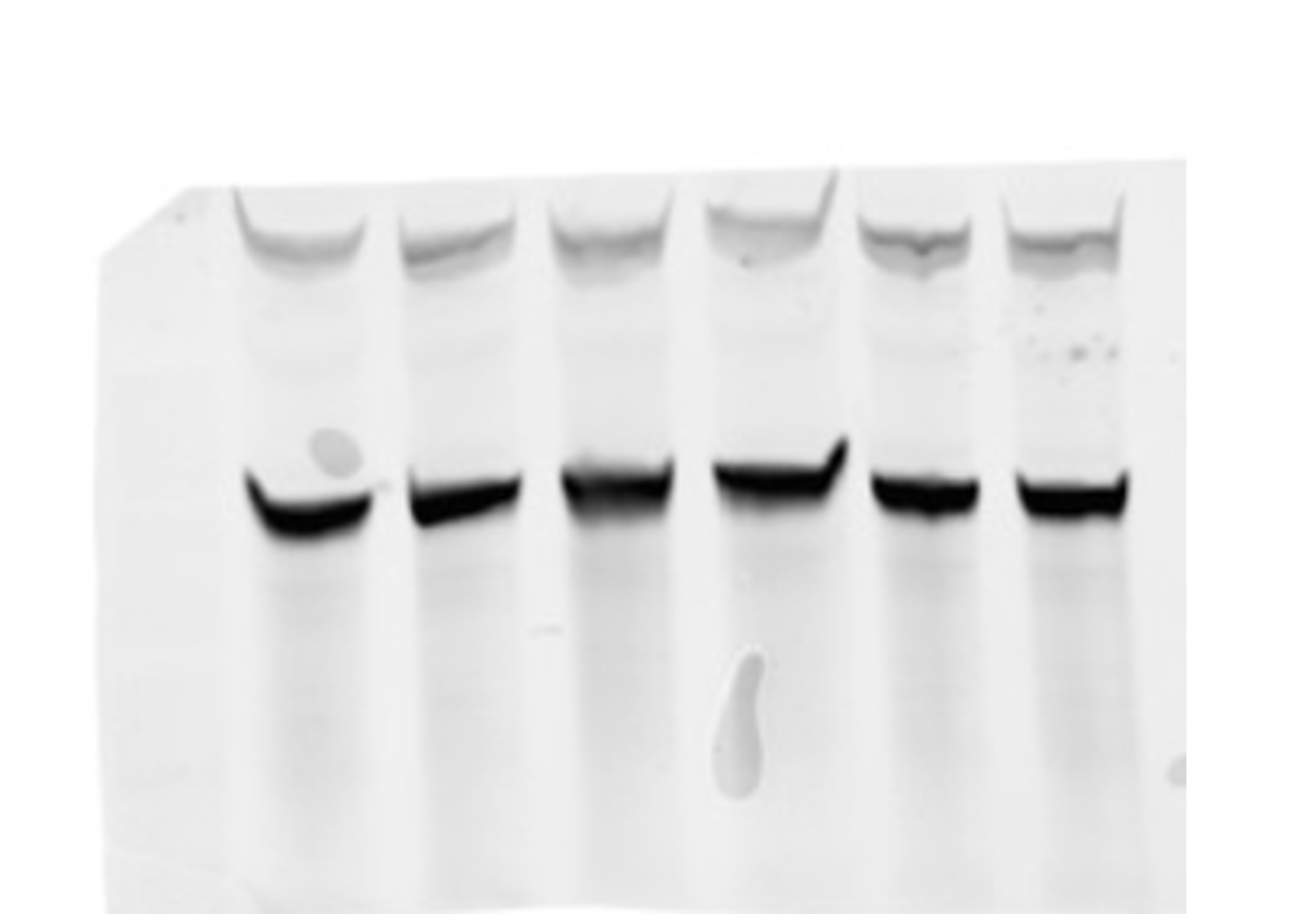

Supplement: S1 File — (ZIP) [file ppat.1012035.s001.zip › S1 File. Original western blot images/Fig 5/Fig 5A actin.tif]

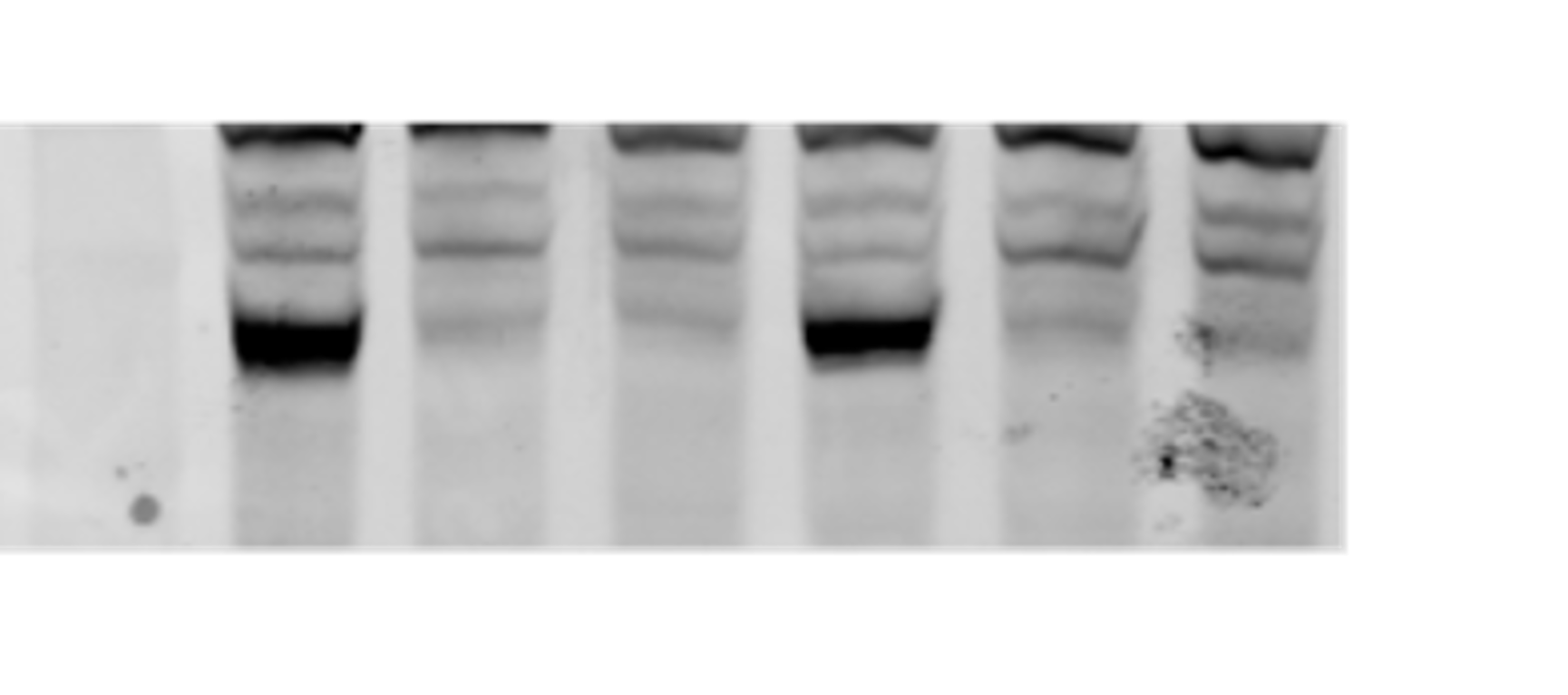

Supplement: S1 File — (ZIP) [file ppat.1012035.s001.zip › S1 File. Original western blot images/Fig 5/Fig 5A PLSCR1.tif]

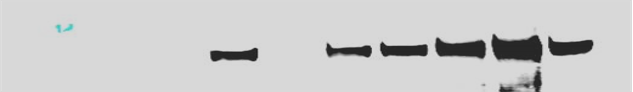

Supplement: S1 File — (ZIP) [file ppat.1012035.s001.zip › S1 File. Original western blot images/Fig 7/Fig 7C Lysate anti-Flag.tif]
